# In Silico Identification of Chikungunya Virus B- and T-Cell Epitopes with High Antigenic Potential for Vaccine Development

**DOI:** 10.3390/v13122360

**Published:** 2021-11-24

**Authors:** Gilma G. Sánchez-Burgos, Nallely M. Montalvo-Marin, Edgar R. Díaz-Rosado, Ernesto Pérez-Rueda

**Affiliations:** 1 Unidad de Investigación Médica Yucatán, Instituto Mexicano del Seguro Social, Mérida 97150, Mexico; nallely.montalvo495@gmail.com (N.M.M.-M.); edgardiaz1995@hotmail.com (E.R.D.-R.); 2 Unidad Académica de Yucatán, Instituto de Investigaciones en Matemáticas Aplicadas y en Sistemas, Universidad Nacional Autónoma de México, Mérida 97302, Mexico; ernesto.perez@iimas.unam.mx

**Keywords:** Chikungunya virus, epitopes, B-cells, T-cells, prediction programs, LBtope

## Abstract

Reverse vaccinology is an outstanding strategy to identify antigens with high potential for vaccine development. Different parameters of five prediction programs were used to assess their sensitivity and specificity to identify B-cell epitopes of Chikungunya virus (CHIKV) strains reported in the IEDB database. The results, based on the use of 15 to 20 mer epitopes and the polyproteins to which they belong, were compared to establish the best parameters to optimize the prediction of antigenic peptides of the Mexican strain CHIKV AJV21562.1. LBtope showed the highest specificity when we used the reported epitopes and polyproteins but the worst sensitivity with polyproteins; ABCpred had similar specificity to LBtope only with the epitopes reported and showed moderate specificity when we used polyproteins for the predictions. Because LBtope was more reliable in predicting true epitopes, it was used as a reference program to predict and select six novel epitopes of the Mexican strain of CHIKV according to prediction frequency, viral genome localization, and non-homology with the human proteome. On the other hand, six bioinformatics programs were used with default parameters to predict T-cell epitopes in the CHIKV strains AJV21562.1 and AJV21561.1. The sequences of the polyproteins were analyzed to predict epitopes present in the more frequent HLA alleles of the Mexican population: DQA1*03011, DQA1*0401, DQA1*0501, DQB1*0201, DQB1*0301, DQB1*0302, and DQB1*0402. Fifteen predicted epitopes in the non-structural and 15 predicted epitopes in the structural polyprotein (9- to 16-mers) with the highest scores of each allele were compared to select epitopes with at least 80% identity. Next, the epitopes predicted with at least two programs were aligned to the human proteome, and 12 sequences without identity with the human proteome were identified as potential antigenic candidates. This strategy would be useful to evaluate vaccine candidates against other viral diseases affecting the countries of the Americas and to increase knowledge about these diseases.

## 1. Introduction

Chikungunya virus (CHIKV) is an enveloped, single-stranded, positive-sense RNA virus belonging to the genus Alphavirus of the family Togaviridae [1,2]. It contains a genome of approximately 12 Kb with two open reading frames with a cap sequence at its 5′ end and a poly(A) at the 3′ end; these two sequences encode two polyproteins cleaved by viral and cellular proteases to generate four non-structural proteins (nsP1, nsP2, nsP3, nsP4) and five structural proteins (C, E3, E2, 6K, E1), respectively [3,4].

Phylogenetic analyses have revealed the presence of three separate lineages of CHIKV strains: West Africa, Asia, and East/South/Central Africa (ESCA), and these lineages are ~92.5–98% identical at the amino acid sequence level [5]. Strains from India and the Indian Ocean are separated into two independent sub-lineages, possibly derived from an East African ancestral genotype [6]. Analyses of complete CHIKV genomes from viruses isolated in 2014 from 14 Caribbean islands, the Bahamas, and two mainland countries in the Americas confirmed that all belonged to the Asian genotype. They clustered together with the other Caribbean and mainland sequences isolated during the American outbreak, forming a monophyletic ‘Asian/American’ lineage that is divided into two well-supported clades [7].

Infections caused by CHIKV generate annual epidemic outbreaks in tropical and subtropical regions in the world where the mosquito species *Aedes aegypti* and *Aedes albopictus* circulate and can transmit this arbovirus. Patients usually have a fever, rash, and joint pain that can persist for months or years, and some patients develop destructive arthropathy/arthritis [8] that can negatively affect their quality of life and their daily usual activities, resulting in significant economic losses to them and challenges to the health systems.

In 2014, more than 1 million suspected cases of chikungunya disease were reported in the Americas. In 2015, in the same region, 693,489 suspected cases and 37,480 confirmed cases of chikungunya fever were reported to the Pan American Health Organization (PAHO) regional office, of which Colombia bore the biggest burden with 356,079 suspected cases. In 2016, a total of 349,936 suspected and 146,914 laboratory-confirmed cases were reported to the PAHO regional office, i.e., half the burden compared to the previous year. Countries reporting the most cases were Brazil (265,000 suspected cases), Bolivia, and Colombia (19,000 suspected cases, in each) [9]. In the case of Mexico, the Ministry of Health reported 11,199 confirmed cases of chikungunya fever in 2015 [10] and 755 cases in 2016 [11]. However, the number of cases may have been higher than reported due to the limitations of the diagnosis. This scenario could easily happen again due to new strains of CHIKV introduced by the waves of migrants that occur annually in Mexico; therefore, we have to find alternatives to diminish the number of cases of chikungunya fever emerging in America.

Vaccination would be of great relevance for the control of this viral infection, and diverse attempts have been made to develop vaccines against CHIKV; however, no approved vaccines are available so far. Thus, it is necessary to develop strategies to design safe and effective vaccines with low cost to apply in countries where CHIKV is endemic.

Since the alphavirus E2 glycoprotein is the major target of the host immune response [12], linear B-cell epitopes are excellent candidates for the development of epitope-based vaccines. In this regard, several approaches to design vaccine candidates against CHIKV have been based on antigenic epitopes predicted with different tools of the IEDB methods, and some of them have been used to construct a multi-epitope vaccine [13,14]). Those epitopes identified in silico were assumed as potentially effective candidates for vaccine development. However, it must be taken into account that some immunoinformatics tools that are designed for B-cell epitopes require refinement to increase their prediction specificity and sensitivity.

In this work, we aimed to establish the best parameters of five computational methods to optimize the prediction of linear B-cell antigenic peptides in the polyprotein of the Mexican CHIKV strain AJV21562.1. In addition, T-cell epitopes of CHIKV with wide allelic distribution in the Mexican population were predicted through six other computational methods. Sequences of 15- to 20-mer B-cell epitopes retrieved from the IEDB database and the polyprotein sequences to which they belong were analyzed using five bioinformatics programs (LBtope, ABCpred, AAP, FBCPred, and BCPred), and the parameters were adjusted to predict a high percentage of the same sequences. The optimized parameters were used to predict B-cell epitopes of the structural polyprotein of the Mexican strain, and the results were compared with the predicted T-cell epitopes in order to identify novel B- and T-cell antigens to develop vaccine candidates.

## 2. Materials and Methods

### 2.1. Selection of Control B-Cell Epitopes

Epitopes from the IEDB database, Version 2.20, were selected as controls based on the following criteria:

1. Epitopes of the alphavirus CHIKV or Sindbis virus structural proteins;
2. Linear B-cell epitopes;
3. Epitopes positive or highly positive by ELISA test using sera from humans infected with CHIKV (positive controls); and
4. Epitopes negative by ELISA or another serological test using sera from humans or primates (negative controls).

### 2.2. Analysis of Specificity and Sensibility of the Control B-Cell Epitopes Prediction

The amino acid sequences of positive and negative controls were analyzed with the programs LBtope [15], ABCpred [16], AAP, FBCPred, and BCPred [17] available on the internet. In each program, the thresholds and the lengths of the amino acids were adjusted according to the length of each control epitope.

ABCpred predicts immunodominant linear B-cell epitopes based on artificial neural networks (machine learning technique) using fixed-length patterns from 10- up to 20-mers [16]. LBtope identifies linear B-cell epitopes based on support vector machines (SVMs) built with more datasets than the ABCpred program. The LBtope program selects epitopes with a percentage of probability of more than 60% for binding to MHC molecules [15]. This program uses the platform of the variable_non_redundant dataset to predict sequences of several sizes. FBCPred predicts epitopes of any length and has been evaluated on unique epitopes without previous homology reduction [17]. The BCPred program predicts B-cell epitopes using the SVM method and was trained on a homology-reduced dataset of linear B-cell epitopes (with more than 80% sequence identity) derived from a dataset previously used to evaluate ABCpred [18]. The length of the epitopes and the threshold can be adjusted by the user. The AAP program is based on an antigenicity scale of paired amino acids (i.e., AAP) from positive epitopes of the BciPep database and negative epitopes of the Swiss-Prot databases [19]. BCPred and AAP allow the user to select between 12 and 22 pairs of amino acids outlets.

The predicted epitopes with more than 80% sequence identity to the control epitopes were used to calculate sensitivity (Sn) and specificity (Sp) according to these formulas:

Sn = TP/(TP + FN)(1)

Sp = TN/(TN + FP)(2)

TP (total true positives) = positives by ELISA and predicted with any program;

TN (total true negatives) = negatives by ELISA and no predicted with any program;

FN (total false negatives) = positives by ELISA and no predicted with any program;

FP (total false positives) = negatives by ELISA and predicted with any program.

### 2.3. Analysis of Specificity and Sensitivity of the Control B-Cell Epitopes Predictions from the Alphavirus Polyproteins

The sequences of the structural polyproteins of the alphavirus PDB ID: 3N42 (DQ462750), PDB ID: 3N44 (DQ462746) [20], PDB ID: 3J2W (AY726732) [21], and PDB ID: 3J0F (JQ771794) [22] were downloaded from the Protein Data Bank. They were analyzed with the aforementioned programs, and we adjusted the thresholds and the number of the amino acids as was done with the control epitopes. The predicted epitopes with >80% similarity to the controls were used to calculate the specificity and sensitivity of each program as previously described. The lowest levels of FP and FN were registered to identify the best thresholds.

### 2.4. Selection of the Best Predictor Programs

The programs LBTope [15], ABCPred [16], and AAP, FBCPred, and BCPred [17]) were used to predict B-cell epitopes which were then compared to select the program with the highest specificity (least amount of FP) as described above, despite having a lower sensitivity (lower amount of TP), because it is preferable to predict fewer true positives than to predict many epitopes including some false positives.

### 2.5. Prediction and Selection of B-Cell Epitopes from a Mexican Strain of CHIKV

LBtope with a threshold of 0.7 was chosen as a reference program to select similar epitopes predicted with the other programs and construct a binary matrix. Briefly, the predicted epitopes in each program were compared with the 20-mer-long epitopes predicted with the LBtope program. Epitopes predicted with at least 60% identity (12 amino acids) with the epitopes predicted by LBtope were labeled with 1; the epitopes not predicted or with less than 60% identity were labeled with 0. The prediction frequency of each epitope was calculated based on the number of programs in which it was predicted.

To select epitopes with the highest antigenic potential, we clustered epitopes with more than 70% overlapping amino acids. Groups containing 10 to 14 overlapping epitopes were divided into two subgroups to homogenize the length of the epitopes in ~20-mers, whereas the epitopes with less than 75% prediction frequency were excluded.

All the epitopes of each group or subgroup were superimposed forming a longer peptide that included all the sequences without repeating them. Finally, amino acids with 100% identity (core) were identified to determine representative antigenic sequences with no identity with the human proteins. To this end, the selected CHIKV sequences were aligned with the human proteome (TaxId: 9606) using the BLAST program, Version 2.10.1, under default conditions.

### 2.6. Prediction and Selection of T-Cell Epitopes from a Mexican Strain of CHIKV

Antigenic T-cell epitopes were identified through a similar approach conducted to identify B-cell epitopes. Briefly, the sequence of the structural and non-structural polyproteins of the CHIKV strains (AJV21562.1 and AJV21561.1, respectively) retrieved from GenBank were analyzed with the default parameters of six computational programs (IEDB Analysis Resource [23], NetMHCIIpan 3.1 [24], RANKPEP [25], PREDIVAC [26], ProPed [27], and EpiTOP 1.0 [28]) to predict epitopes presented by the more frequent HLA alleles in the Mexican population DQA1*03011, DQA1*0401, DQA1*0501, DQB1*0201, DQB1*0301, DQB1*0302, and DQB1*0402.

IEDB includes a variety of binding prediction algorithms (machine learning-based methods (ARB, NN-align, SMM-align), a combinatorial library (PROPED), and a combined consensus approach trained with the complete dataset [23]) to determine the affinity of a panel of peptides to the MHC class II molecules covering 26 allelic variants with high frequency in the human population [23]. NetMHCIIpan 3.1 is based on an ensemble of artificial neural networks trained on quantitative peptide binding data covering multiple MHC class II molecules. The identification of the binding core by neural networks ensembles is improved with the network alignment procedure called “offset correction” (fully automated and unsupervised, which means that no information about the actual location of the binding core is used to define the offset values) [24]. RANKPEP uses position-specific scoring matrices (PSSMs) or profiles for the prediction of peptide-MHC class I and peptide-MHC class II binding. The server determines whether the C terminus of any predicted MHC class I ligand may result from proteasomal cleavage. Predictions are focused on conserved T-cell epitopes to thwart mutation as an immune evasion mechanism [25]. The PREDIVAC predictions are based on the concept of specificity-determining residues applied to the protein phosphorylation site prediction. The method was developed using high-affinity HLA class II peptide binding data because of the correlation with promiscuous CD4+ T-cell recognition and immunodominance. A central finding was the highest specificity delivered by the method in the identification of immunodominant and promiscuous CD4+ T-cell epitopes [26]. ProPed is a graphical tool for predicting MHC class II binding regions. The server implements a matrix-based prediction algorithm, employing an amino acid/position coefficient table deduced from the literature, and might be useful in locating the promiscuous binding regions that can bind to several HLA-DR alleles [27]. The EpiTOP 1.0 server predicts MHC class II binding based on a quantitative structure/activity relationship for ligands binding to 12 HLA-DRB1 alleles. Models derived were based on combinations of different blocks of variables (cross-terms accounting for adjacent positions, for every second position in the peptide, and for peptide/protein interactions). The external predictive ability was tested using a set of 356 HLA-DRB1 binders with an r2 from 0.364 to 0.530. Peptide and protein positions involved in the interactions were analyzed in terms of hydrophobicity, steric bulk, and polarity [28].

Fifteen predicted epitopes in non-structural and 15 predicted epitopes in the structural polyprotein (9- to 16-mers), with the highest scores of each allele, were compared to select epitopes with at least 80% identity. The prediction frequency of each epitope was calculated based on the number of programs in which it was predicted, and the epitopes predicted by fewer than two programs were discarded. Finally, the selected sequences were compared with the human proteome (TaxId: 9606) using the BLAST program, Version 2.10.1, under default conditions.

### 2.7. Structural Localization of the Identified Peptides

The sequences of the new epitopes predicted in this work, localized in the structural polyprotein of CHIKV were mapped in the crystal structure of the protein retrieved from the PDB ID 3N41 by using the PyMOL Molecular Graphics System, Version 2.0 [29].

### 2.8. Antigenicity Prediction

To determine whether the predicted epitopes induce T- and B-cell immune responses, we analyzed the sequence of the CHIKV strain AJV21562.1 with VaxiJen [30]. In this regard, the analysis considered the antigenic prediction to MHC class II molecules by using the IEDB consensus and Vaxitop comparisons. Finally, we evaluated if the epitopes were significantly predicted with both methods.

## 3. Results

### 3.1. Selection of Positive Control and Negative Control B-Cell Epitopes

Twenty-four positive epitopes and 33 negative epitopes were selected from the IEDB database, in which they were registered with positive or highly positive immunoreactivity or negative against CHIKV antibodies in infected human sera (Table 1).

### 3.2. Program Validation Based on Predictions of Control B-Cell Epitopes

We obtained predictions with high Sp values (0.515–0.939) using a threshold of 0.7 to 0.85 (Table 2). All programs predicted more than 50% FP and less than 50% TP, with low Sn values (0.167–0.50).

The programs with the highest Sp were LBtope (Sp 0.939) and ABCpred (Sp 0.909), followed by BCPred and FBCPred (both with Sp 0.727) and AAP (Sp 0.515) (Table 2). In contrast, the LBtope and ABCpred programs predicted the least amount of FP and had the highest Sp, even though they had the lowest Sn (Table 2). The FBCPred and BCPred programs showed an Sn (>0.4) twice the Sn of ABCpred and LBtope (Table 2). Finally, the highest number of FP control epitopes were predicted with AAP; therefore, this program was discarded for the following analysis.

### 3.3. Validation of Programs Based on Control B-Cell Epitopes Predicted in Alphavirus Polyproteins

To confirm the validation of the programs, we retrieved from PDB the alphavirus sequences PDB ID: 3N42 (DQ462750), PDB ID: 3N44 (DQ462746) [20], PDB ID: 3J2W (AY726732) [21], and PDB ID: 3J0F (JQ771794) [22]. These structural polyproteins were analyzed with LBtope, ABCpred, BCPred, and FBCPred, as described in the Materials and Methods section (Section 2). The predicted epitopes were compared with the control epitopes to calculate the amount of TP, FN, TN, and FP, as well as Sn and Sp (Table 3).

The program with the highest Sp was LBtope (Sp 0.909), followed by FBCPred and BCPred (each with Sp 0.636). The ABCpred program showed a slightly lower Sp (0.606) than FBCPred and BCPred (Table 3). Based on polyproteins, FBCPred and BCPred predicted more TP (66.7% and 54.2%, respectively) using polyproteins than ABCpred (45.8%) and LBtope (20.8%) (Table 3). ABCpred, FBCPred, and BCPred predicted more FP (36.4% to 39.4%) than LBtope (9.1%) with the polyproteins (Table 3). FBCPred, BCPred, and ABCpred did not discriminate many positive epitopes from negative epitopes using polyproteins even with a threshold greater than 0.7 (Table 3). Therefore, all programs predicted more than 50% FP and less than 50% TP, observing Sn values between 0.208 and 0.667. Finally, Sn increased in all programs using polyproteins for the prediction of epitopes. The program with the highest Sn was FBCPred (0.667), followed by BCPred (0.542), ABCpred (0.458), and LBtope (0.208).

### 3.4. Prediction and Selection of Novel B-Cell Epitopes of the Mexican CHIKV Strain

LBtope was selected as the best predictor program due to its highest Sp using sequences of positive control epitopes and structural polyproteins of CHIKV. Therefore, this program (dataset variable_non_redundant) with a threshold of 0.7 was used to predict 171 (20-mer-long) epitopes from the CHIKV strain AJV21562.1, which were compared to epitopes predicted with ABCPred, FBCPred, and BCPred with a threshold of 0.8 each. A total of 145 epitopes with more than 60% identity were identified. Fifty-two epitopes (30.40%) were predicted with one program in addition to LBtope, 76 epitopes (44.44%) were predicted with two additional programs, and 17 epitopes (9.94%) were predicted with three or more programs.

After elimination of 17 sequences with more than 80% of identity to the reported sequence in the IEDB database, used as positive and negative controls (Table 1), 13 clusters of 1 to 10 epitopes with more than 70% overlapping amino acids were obtained. Because most of the epitopes included in the clusters were predicted with one or two programs (less than 75% prediction frequency) 70 epitopes were not chosen as a potential antigen according to our criteria. Only seven new epitopes of CHIKV AJV21562.1 predicted with all programs were identified as a potential candidate highly antigenic, three epitopes in the E2 protein and two epitopes of the E1 protein, whereas one epitope was located in the S3 peptidase and one epitope was in a non-coding region (amino acids 61–80).

An epitope with 45% similarity to the human immunoglobulin heavy chain junction human region (ID MOP20971.1 and MOK15443.1) was deleted. Thus, six sequences of 20–26 amino acids were identified as novel antigens potentially immunogenic and without identity to the human proteome, which would avoid cross-reactivity with human antigens (Table 4).

### 3.5. Prediction and Selection of Novel T-Cell Epitopes

A total of 210 T-cell epitopes predicted with bioinformatics programs were selected for the seven DQ alleles, with more frequency in the Mexican population. Ninety-five (45.2%) epitopes were predicted with the highest scores in most of the programs, of which 21% had the same length, 60% shared at least 80% identity, and 19% had >80% identity and the same scores in all programs. Finally, 12 sequences predicted by two or more programs with the highest scores were identified as potential antigenic candidates (Table 5). None of these epitopes showed identity with human proteins. The sequence RPGYSPMVLEMEL in the epitope EC17-RPG was also predicted by LBTope, but it was only included only in Table 5 because it was more frequently predicted with methods for the T-cell epitopes prediction.

### 3.6. Mapping of the Identified Peptides

**Figure 1:**
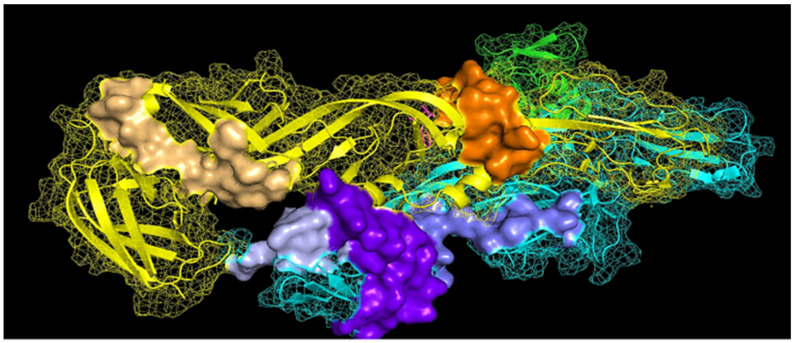
Three-dimensional localization of the predicted B-cell epitopes of CHIKV strain AJV21562.1. The pep25 (blue), pep157 (deep purple), and pep114 (light blue) are in the E2 protein. The pep91 (light orange) and pep107 (orange) are in the E1 protein.

The structural polyprotein from the PDB ID: 3N41 sequence was modeled by using the PyMOL Molecular Graphics System, Version 2.0 [29], to map the linear B-cell epitopes predicted in this work. The epitopes pep25 (blue), pep114 (light blue), and pep157 (deep purple) were together in the E2 protein, whereas pep91 (light orange) was localized in the C-terminal, and pep107 (orange) was in the N-terminal of the E1 protein (Figure 1). The pep157 was completely exposed on the surface of the E2 protein, while the other epitopes were partially embedded in E1 or E2 proteins.

**Figure 2:**
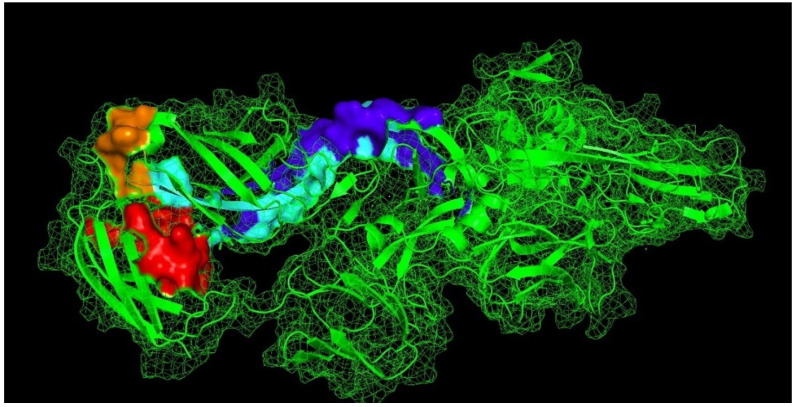
Three-dimensional localization of the predicted T-cell epitopes of Chikungunya virus strain AJV21562.1. The epitopes EC17-RPG (orange), EC38-MTN (red), EC20-MVL (cyan), and EC15-EFA (blue) were localized in the E1 protein.

The T-cell epitopes EC17-RPG (orange) and EC38-MTN (red) were partially exposed, whereas EC20-MVL (cyan) and EC15-EFA (blue) were embedded in the E1 protein (Figure 2).

The epitopes pep2, EC6-CST, EC18-QPL, and EC4-GVG could not be mapped in the 3N41 protein. In addition, we identified two new epitopes of the nsP1 and one epitope in the protein nsP2, nsP3, and nsP4 proteins (data not shown) restricted to the MHC class II complex.

### 3.7. Antigenicity Prediction

The CHIKV strain AJV21562.1 sequence was analyzed with VaxiJen [30]. The predicted epitopes pep25, pep157, pep91, pep107, EC6-CST, EC18-QPL, EC17-RPG, EC20-MVL, EC15-EFA, EC38-MTN, and EC4-GVG, which are associated with Alpha E2 or Alpha E1 proteins, were also identified by the IEDB consensus, whereas the pep2, pep114, EC12-PFM, EC2-LQA (nsP1 protein), EC30-NQL (nsP2), EC31-PSD (nsP3), and EC27-VHT (nsP4) epitopes were not predicted. Vaxitop was only able to predict the epitopes EC6-CST, EC18-QPL, EC17-RPG, and EC15-EFA (Supplementary Information Table S1).

## 4. Discussion

In order to improve the specificity and sensitivity of our prediction of B-cell epitopes, several thresholds in computational programs were used to analyze the prediction of control epitopes retrieved from the IEDB. As we expected, increasing the prediction thresholds in some programs allowed us to discriminate between positive and negative epitopes and to choose true antigenic epitopes. The LBtope and ABCpred programs predicted the least amount of FP and had the highest Sp using control epitopes. Therefore, they were the most reliable programs, even though they had the lowest Sn (Table 2 and Table 3). However, ABCpred predicted more FP than LBtope, with the polyproteins suggesting that ABCpred is a moderately good predictor. In contrast, the LBtope program predicted the lowest amount of FP and TP by analyzing control epitopes (16.7%) as well as polyproteins (20.8%) (Table 2 and Table 3), resulting in the best predictor. Finally, FBCPred and BCPred could not discriminate between positive and negative control epitopes with both strategies. Therefore, FBCPred and BCPred were considered poor predictors.

In general, when alphavirus polyproteins sequences were used to predict CHIKV epitopes, Sn increased and Sp decreased in all programs. This suggests that more TP and more FP could be predicted, as more neighboring amino acids flank the epitopes, since changing an amino acid disrupts the epitope/antibody interaction [33]. In this sense, Chua et al. [21] identified some mutations of neutralizing epitopes that alter the efficiency of cross-neutralization between CHIKV genotypes.

Several of the positive control epitopes have not been predicted as TP, probably due to different algorithms and models of the bioinformatics programs used, since the programs are based on different properties of the proteins or do not take into account the reconfiguration of epitope residues when an antigen is in complex with a specific antibody [34]. However, some TP epitopes were predicted with more than 80% similarity to the positive controls, suggesting that it is necessary to adjust the conditions of the prediction programs (as done in this work) to eliminate many FP and have greater specificity for future predictions.

Most antibodies against CHIKV recognize the region between amino acids 3 and 10 (STKDNFNVYK) of the peptide E2EP3 STKDNFNVYKATRPY [33], which was used as a positive control in our work. The sequence TKDNFNVYK was predicted only with ABCpred in two of our TP epitopes predicted from positive control epitopes and the polyproteins analyzed. We identified the amino acids TKDNFNVYK in 50% and 75% of two similar TP. Additional amino acids may have reduced the predictive power of the other programs.

Narula et al. [14] identified B-cell, Th, and CTL epitopes, using the FBCPred program with a threshold greater than 0.8, similar to our methods. The B-cell epitope VTWGNNEPYKYWPQLSANGT of the CHIKV African strain S27 (UniProt ID: Q8JUX5) was similar in two epitopes predicted in this work. Precisely 65% and 95% of the African strain epitope are shared with two different TP epitopes predicted from alphavirus polyproteins or the positive control epitopes.

In addition, with both strategies, we predicted a TP epitope and one FP epitope containing 30% and 50%, respectively, of the B-cell epitope PLVPRNAELGDRKGKIHIPF [14].

On the other hand, the amino acids LPCST of the CD4+ epitope LPCSTYVQSNAATAEE [14] were identified in one FP (33.3%), and the sequence LPCSTYVQS was observed in 60% of another FP epitope predicted with both strategies.

Based on the above findings, we suggest that the prediction of FP with bioinformatics programs does not depend on sequences used.

The CHIKV sequences used in this work had 80% amino acid conservation, even with other alphaviruses. Therefore, the epitopes predicted and selected with our strategy could be effective to bind to antibodies from patients in different endemic regions.

We consider that our new epitopes of CHIKV identified in silico have high antigenic potential, since they were predicted using a strategy based on a similar prediction of epitopes already validated experimentally, which is completely different from the other in silico strategies used so far. Furthermore, as some of our new epitopes have some percentage of similarity with the epitopes reported by Narula et al. [14], we expect that the overlapped region of CHIKV reported in our work and by Narula and colleagues [14] can mediate a strong antibody response against the viral infection. Therefore, the epitopes from the overlapped region would be more likely to recognize antibodies against CHIKV than epitopes identified by strategies that did not include experimentally validated epitopes.

In addition, to determine the antigenicity of all of the 18 newly identified B and T-cell epitopes, we analyzed the sequence of CHIKV strain AJV21562.1 with VaxiJen [30], considering the antigenic prediction to MHC class II, by using the IEDB consensus and Vaxitop comparisons. We found that 4 out 6 predicted epitopes from Table 4 and 12 epitopes from Table 5 were also predicted by the IEDB consensus, whereas 1 epitope from the E2 protein and 3 epitopes from E1 protein were predicted with Vaxitop (Table S1), probably because no training data are available or were used for conducting the requested prediction, as the server claims. However, we must be careful, because VaxiJen predictions are mainly based on bacterial datasets, and not enough virus genomes have been considered.

## 5. Conclusions

We evaluated different conditions within several computer programs to increase the probability of prediction of antigenically known positive and negative epitopes of alphavirus, and this allowed us to identify six new linear B-cell epitopes of CHIKV with high antigenic potential. Using six computational methods, we improved the identification of 12 new T-cell epitopes to induce cellular immune responses against CHIKV. This work also shows that it is necessary to adjust the threshold of the prediction programs to eliminate many FP and increase the specificity and probability of predicting true antigens by immunoinformatics for the development of safe and effective vaccines regardless of the viral strains. Our strategy would be useful for developing vaccine candidates against other viral diseases.

## Figures and Tables

**Table 1:** Control epitopes used in this work. Epitopes with positive or negative immunoreactivity were selected from the IEDB database [20,21,22], Lum et al., 2013 [31], and Kam et al., 2014 [32]. aa, amino acids.

Epitopes from the IEDB Databases				
No.	Negative Epitopes	Length in aa	Positive Epitopes	Length in aa
1	LAHCPDCGEGHSCHS	15	STKDNFNVYKATRPYLAHC	19
2	DCGEGHSCHSPVALE	15	PTEGLEVTWGNNEPYKYWPQLSTNGT	26
3	HSCHSPVALERIRNE	15	LLSMVGMAAGMCMCARRRCITPYELTPGATVPFL	34
4	PVALERIRNEATDGT	15	TDGTLKIQVSLQIGIKTDDSHDWTKLRYMDNHMPADAERAGL	42
5	RIRNEATDGTLKIQV	15	LTTTDKVINNCKVDQCHA	18
6	LKIQVSLQIGIKTDD	15	LTTTDKVINNCKVDQCHAAVTNHKKW	26
7	SLQIGIKTDDSHDWT	15	PTVTYGKNQVIMLLYPDHPTLLSYRN	26
8	IKTDDSHDWTKLRYM	15	STKDNFNVYKATRPY	15
9	SHDWTKLRYMDNHMP	15	CTITGTMGHFILARC	15
10	KLRYMDNHMPADAER	15	NHKKWQYNSPLVPRN	15
11	DNHMPADAERAGLFV	15	HIPFPLANVTCRVPK	15
12	ADAERAGLFVRTSAP	15	VTYGKNQVIMLLYPD	15
13	AGLFVRTSAPCTITG	15	LEVTWGNNEPYKYWP	15
14	RTSAPCTITGTMGHF	15	GTAHGHPHEIILYYY	15
15	ILARCPKGETLTVGF	15	HPHEIILYYYELYPT	15
16	TMGHFILARCPKGET	15	KDIVTKITPEGAEEW	15
17	PKGETLTVGFTDSRK	15	LLQASLTCSPHRQRR	15
18	LTVGFTDSRKISHSC	15	EITVMSSEVLPSTNQEYI	18
19	ISHSCTHPFHHDPPV	15	HVKGTIDHPVLSKLKFTK	18
20	EVVLTVPTEGLEVTW	15	KPGKRQRMALKLEADRLF	18
21	IGREKFHSRPQHGKE	15	NIPISIDIPNAAFIRTSD	18
22	FHSRPQHGKELPCST	15	PISASFTPFDHKVVIHRG	18
23	QHGKELPCSTYVQST	15	TWNSKGKTIKTTPEGTEE	18
24	LPCSTYVQSTAATTE	15	YNYDFPEYGAMKPGAFGD	18
25	YVQSTAATTEEIEVH	15		
26	MPPDTPDRTLMSQQS	15		
27	PDRTLMSQQSGNVKI	15		
28	MSQQSGNVKITVNGQ	15		
29	TVNGQTVRYKCNCGG	15		
30	TVRYKCNCGGSNEGL	15		
31	SNEGLTTTDKVINNC	15		
32	TTTDKVINNCKVDQC	15		
33	QYNSPLVPRNAELGD	15		

**Table 2:** Thresholds of each program with the highest amount of TP and the lowest amount of TN control epitopes. The analysis of positive and negative control epitopes was made with each program using different thresholds and lengths. The number of predicted TP, TN, FP, and FN sequences were used to calculate sensitivity (Sn) and specificity (Sp).

Program	ABCpred	FBCPred	LBTope	BCPred	AAP
Thresholds	0.85	0.85	0.7	0.8	0.7
Sn	0.208	0.458	0.167	0.417	0.500
Sp	0.909	0.727	0.939	0.727	0.515
TP	5 (20.8%)	11 (45.8%)	4 (16.7%)	10 (41.7%)	12 (50%)
FN	19 (79.2%)	13 (54.2%)	20 (83.3%)	14 (58.3%)	12 (50%)
TN	30 (90.9%)	24 (72.7%)	31 (93.9%)	24 (72.7%)	17 (51.5%)
FP	3 (9.1%)	9 (27.3%)	2 (6.1%)	9 (27.3%)	16 (48.5%)

**Table 3:** Thresholds of each program with the highest amount of TP and the least amount of TN control epitopes predicted from structural polyproteins. The analysis of positive and negative control epitopes was made with each program using different thresholds and lengths. The number of predicted TP, TN, FP, and FN sequences was used to calculate Sn and (Sp).

Program	ABCpred	FBCPred	LBTope	BCPred
Thresholds	0.85	0.85	0.7	0.8
Sn	0.458	0.667	0.208	0.542
Sp	0.606	0.636	0.909	0.636
TP	11 (45.8%)	16 (66.7%)	5 (20.8%)	13 (54,2%)
FN	13 (54.2%)	8 (33.3%)	19 (79.2%)	11 (45.8%)
TN	20 (60.6%)	21 (63.6%)	30 (90.9%)	21 (63.6%)
FP	13 (39.4%)	12 (36.4%)	3 (9.1%)	12 (36.4%)

**Table 4:** Predicted B-cell epitopes of CHIKV Mexican strain. Epitopes of the structural polyprotein AJV21562.1 were predicted with the programs LBtope (dataset variable_non_redundant), ABCPred, FBCPred, and BCPred (see Materials and Methods section (Section 2)).

ID	Protein	Position (Start)	Position (End)	Sequence
pep2	N/A	61	83	PRKNRKNKKQKQKQQAPRNNTNQ
pep25	Alpha E2	451	471	THPFHHDPPVIGREKFHSRPQ
pep157	Alpha E2	618	643	TLLSYRNMGEEPNYQEEWVTHKKEIR
pep91	Alpha E1	815	834	VIPNTVGVPYKTLVNRPGYS
pep107	Alpha E1	999	1018	PPFGAGRPGQFGDIQSRTPE
pep114	Alpha E2	646	665	VPTEGLEVTWGNNEPYKYWPQ

**Table 5:** Predicted T-cell epitopes of CHIKV Mexican strain. Epitopes of the structural AJV21562.1 and non-structural AJV21561.1 polyproteins of CHIKV were predicted with the programs IEDB Analysis Resource, NetMHCIIpan 3.1, RANKPEP, PREDIVAC, ProPed, and EpiTOP 1.0 (see Materials and Methods section (Section 2)).

Epitope ID	Sequence	Position	Protein	Allele
EC12-PFM	PFMYNAMAGAYPSYST	182–197	nsP1	HLA-DQA1*05:01/DQB1*03:01
EC6-CST	CSTYAQSTAATAEEIEVHM	478–496	E2	HLA-DQA1*04:01/DQB1*04:02 HLA-DQA1*03:01/DQB1*03:02
EC2-LQA	LQAAQEDVQVEIDVEQLED	513–531	nsP1	HLA-DQA1*03:01/DQB1*03:02 HLA-DQA1*04:01/DQB1*04:02 HLA-DQA10501-DQB10201
EC18-QPL	QPLFWMQALIPLAAL	763–777	E1	HLA-DQA10501-DQB10201
EC17-RPG	RPGYSPMVLEMELLSVTLE	830–848	E1	HLA-DQA10501-DQB10201
EC20-MVL	MVLEMELLSVTLEPTL	836–850	E1	HLA-DQA10501-DQB10201
EC15-EFA	EFASAYRAHTASASAKLRV	926–944	E1	HLA-DQA1*05:01/DQB1*03:01
EC38-MTN	MTNAVTIREAEIEVE	1142–1156	E1	HLA-DQA1*03:01/DQB1*03:02
EC4-GVG	GVGLVVAVAALILIV	1225–1239	E1	HLA-DQA1*05:01/DQB1*03:01
EC30-NQL	NQLNAAFVGQATRAG	1317–1331	nsP2	HLA-DQA1*05:01/DQB1*03:01
EC31-PSD	PSDLDADAPALEPAL	1689–1704	nsP3	HLA-DQA1*03:01/DQB1*03:02
EC27-VHT	VHTLFDMSAEDFDAI	2200–2216	nsP4	HLA-DQA10501-DQB10201

## Data Availability

Not applicable.

## Supplementary Materials

The following are available online at https://www.mdpi.com/article/10.3390/v13122360/s1, Table S1: Antigenicity prediction of epitopes from the CHIKV Mexican strain.

## References

[1] Higashi N., Matsumoto A., Tabata K., Nagatoma Y. (1967). Electron microscope study of development of Chikungunya virus in green monkey kidney stable (VERO) cells. Virology.

[2] Powers A.M., Brault A.C., Shirako Y., Strauss E.G., Kang W., Strauss J.H., Weaver S.C. (2001). Evolutionary relationships and systematics of the alphaviruses. J. Virol..

[3] Simizu B., Yamamoto K., Hashimoto K., Ogata T. (1984). Structural proteins of Chikungunya virus. J. Virol..

[4] Khan A.H., Morita K., Del Carmen Parquet M., Hasebe F., Mathenge E.G.M., Igarashi A. (2002). Complete nucleotide sequence of chikungunya virus and evidence for an internal polyadenylation site. J. Gen. Virol..

[5] Quiroz J.A., Malonis R.J., Thackray L.B., Cohen C.A., Pallesen J., Jangra R.K., Brown R.S., Hofmann D., Holtsberg F.W., Shulenin S. (2019). Human monoclonal antibodies against chikungunya virus target multiple distinct epitopes in the E1 and E2 glycoproteins. PLoS Pathog..

[6] Grandadam M., Caro V., Plumet S., Thiberge J.M., Souarès Y., Failloux A.B., Tolou H.J., Budelot M., Cosserat D., Leparc-Goffart I. (2011). Chikungunya virus, Southeastern France. Emerg. Infect. Dis..

[7] Sahadeo N.S.D., Allicock O.M., De Salazar P.M., Auguste A.J., Widen S., Olowokure B., Gutierrez C., Valadere A.M., Polson-Edwards K., Weaver S.C. (2017). Understanding the evolution and spread of chikungunya virus in the Americas using complete genome sequences. Virus Evol..

[8] Bouquillard E., Combe B. (2009). Rheumatoid arthritis after Chikungunya fever: A prospective follow-up study of 21 cases. Ann. Rheum. Dis..

[9] World Health Organization Chikungunya 12 April 2017. Disease Outbreaks. https://www.who.int/news-room/fact-sheets/detail/chikungunya.

[10] Secretaría de Salud/SINAVE/DGE Boletín Epidemiológico. Semana 48; 2015, No 48 Volume 32. https://www.gob.mx/salud/acciones-y-programas/informacion-epidemiologica.

[11] Secretaría de Salud/SINAVE/DGE Boletín Epidemiológico. Semana 50; 2016, No 50 Volume 33. https://www.gob.mx/salud/acciones-y-programas/historico-boletin-epidemiologico.

[12] Fong R.H., Banik S.S.R., Mattia K., Barnes T., Tucker D., Liss N., Lu K., Selvarajah S., Srinivasan S., Mabila M. (2014). Exposure of Epitope Residues on the Outer Face of the Chikungunya Virus Envelope Trimer Determines Antibody Neutralizing Efficacy. J. Virol..

[13] Qamar M.T.U., Bari A., Adeel M.M., Maryam A., Ashfaq U.A., Du X., Muneer I., Ahmad H.I., Wang J. (2018). Peptide vaccine against chikungunya virus: Immuno-informatics combined with molecular docking approach. J. Transl. Med..

[14] Narula A., Pandey R.K., Khatoon N., Mishra A., Prajapati V.K. (2018). Excavating chikungunya genome to design B and T cell multiepitope subunit vaccine using comprehensive immunoinformatics approach to control chikungunya infection. Infect. Genet. Evol..

[15] Singh H., Ansari H.R., Raghava G.P.S. (2013). Improved method for linear B-cell epitope prediction using antigen’s primary sequence. PLoS ONE.

[16] Saha S., Raghava G.P.S. (2006). Prediction of continuous B-cell epitopes in an antigen using recurrent neural network. Proteins.

[17] El-Manzalawy Y., Dobbs D., Honavar V. (2008). Predicting linear B-cell epitopes using string kernels. J. Mol. Recognit..

[18] Potocnakova L., Bhide M., Pulzova L.B. (2016). An Introduction to B-Cell Epitope Mapping and In Silico Epitope Prediction. J. Immunol. Res..

[19] Chen J., Liu H., Yang J., Chou K.C. (2007). Prediction of B-cell epitopes using amino acid pair antigenicity scale. Amino Acids.

[20] Kam Y.W., Lee W.W., Simarmata D., Harjanto S., Teng T.S., Tolou H., Chow A., Lin R.T., Leo Y.S., Rénia L. (2012). Longitudinal Analysis of the Human Antibody Response to Chikungunya Virus Infection: Implications for Serodiagnosis and Vaccine Development. J. Virol..

[21] Chua C.L., Sam I.C., Merits A., Chan Y.F. (2016). Antigenic Variation of East/Central/South African and Asian Chikungunya Virus Genotypes in Neutralization by Immune Sera. PLoS Negl. Trop. Dis..

[22] Adouchief S., Smura T., Vapalahti O., Hepojoki J. (2016). Mapping of human B-cell epitopes of Sindbis virus. J. Gen. Virol..

[23] Wang P., Sidney J., Kim Y., Sette A., Lund O., Nielsen M., Peters B. (2010). Peptide binding predictions for HLA DR, DP and DQ molecules. BMC Bioinform..

[24] Andreatta M., Karosiene E., Rasmussen M., Stryhn A., Buus S., Nielsen M. (2015). Accurate pan-specific prediction of peptide-MHC class II binding affinity with improved binding core identification. Immunogenetics.

[25] Reche P.A., Glutting J.P., Zhang H., Reinherz E.L. (2004). Enhancement to the RANKPEP resource for the prediction of peptide binding to MHC molecules using profiles. Immunogenetics.

[26] Oyarzún P., Ellis J.J., Bodén M., Kobe B. (2013). PREDIVAC: CD4+ T-cell epitope prediction for vaccine design that covers 95% of HLA class II DR protein diversity. BMC Bioinform..

[27] Singh H., Raghava G.P.S. (2001). ProPred: Prediction of HLA-DR binding sites. Bioinformatics.

[28] Dimitrov I., Garnev P., Flower D.R., Doytchinova I. (2010). Peptide binding to the HLA-DRB1 supertype: A proteochemometrics analysis. Eur. J. Med. Chem..

[29] PyMOL Molecular Graphics System, Version 2.0 Schrödinger, LLC. https://pymol.org/.

[30] He Y., Xiang Z., Mobley H.L.T. (2010). Vaxign: The first web-based vaccine design program for reverse vaccinology and applications for vaccine development. J. Biomed. Biotechnol..

[31] Lum F.M., Teo T.H., Lee W.W., Kam Y.W., Rénia L., Ng L.F.P. (2013). An essential role of antibodies in the control of Chikungunya virus infection. J. Immunol..

[32] Kam Y.W., Lee W.W., Simarmata D., Le Grand R., Tolou H., Merits A., Roques P., Ng L.F.P. (2014). Unique Epitopes Recognized by Antibodies Induced in Chikungunya Virus-Infected Non-Human Primates: Implications for the Study of Immunopathology and Vaccine Development. PLoS ONE.

[33] Kam Y.W., Lum F.M., Teo T.H., Lee W.W., Simarmata D., Harjanto S., Chua C.L., Chan Y.F., Wee J.K., Chow A. (2012). Early neutralizing IgG response to Chikungunya virus in infected patients targets a dominant linear epitope on the E2 glycoprotein. EMBO Mol. Med..

[34] Zhao L., Wong L., Li J. (2011). Antibody-specified B-cell epitope prediction in line with the principle of context-awareness. IEEE/ACM Trans. Comput. Biol. Bioinform..

