# Supplementary material for: In Silico Identification of Chikungunya Virus B- and T-Cell Epitopes with High Antigenic Potential for Vaccine Development
Source: Viruses. 2021 Nov 24;13(12):2360. doi: 10.3390/v13122360 (PMC8706625; doi:10.3390/v13122360)
Supplement: Supplementary file 1 [file viruses-13-02360-s001.zip › viruses-1305663-supplementary.pdf]

Table S1. Antigenicity prediction of epitopes from the CHIKV Mexican strain. Bold black letters are predicted B-cell epitopes, bold blue letters are predicted T-cell epitopes.

Epitope predicted by IEDB consensus method							Epitope predicted by Vaxitop method						
Index	Epitope	MHC allele	Matching from	Matching to	Percentile	P-value	Index	Epitope	MHC Allele	Matching from	Matching to	Percentile	P-value
1485	MEFIPTQTFYNRRYQ	HLA-DRB1*07:01	1	15	5.95	>0.1	109	PTQTFYNRR	HLA-DRB1*15:01	5	13	0.043	Not significant
1714	MEFIPTQTFYNRRYQ	HLA-DRB1*08:01	1	15	6.87	Not predicted	115	TFYNRRYQP	HLA-DRB1*11:01	8	16	0.0454	Not significant
1764	MEFIPTQTFYNRRYQ	HLA-DRB1*13:01	1	15	6.91	>0.1	155	RPTIQVIRP	HLA-DRB1*11:01	22	30	0.0629	5.4
2428	MEFIPTQTFYNRRYQ	HLA-DRB1*11:01	1	15	9.53	>0.1	160	RPTIQVIRP	HLA-DRB1*03:01	22	30	0.0643	1.28
1715	EFIPTQTFYNRRYQP	HLA-DRB1*08:01	2	16	6.87	Not predicted	205	RPTIQVIRP	HLA-DRB1*07:01	22	30	0.0966	Not significant
2429	EFIPTQTFYNRRYQP	HLA-DRB1*11:01	2	16	9.53	>0.1	147	KAGQLAQLI	HLA-DRB1*15:01	37	45	0.0598	Not significant
1716	FIPTQTFYNRRYQPR	HLA-DRB1*08:01	3	17	6.87	Not predicted	145	AQLISAVNK	HLA-DRB1*03:01	42	50	0.0588	5.24

2430	FIPTQTFYNRRYQPR	HLA-DRB1*11:01	3	17	9.53	>0.1	65	ISAVNKLTM	HLA-DRB1*01:01	45	53	0.0252	Not significant
1220	IPTQTFYNRRYQPRP	HLA-DRB1*08:01	4	18	4.81	Not predicted	104	ISAVNKLTM	HLA-DRB1*07:01	45	53	0.0401	Not significant
1221	PTQTFYNRRYQPRPW	HLA-DRB1*08:01	5	19	4.81	Not predicted	96	AVNKLTMRV	HLA-DRB1*11:01	47	55	0.0366	3.42
1222	TQTFYNRRYQPRPWT	HLA-DRB1*08:01	6	20	4.81	Not predicted	165	KPRKNRKNK	HLA-DRB1*03:01	60	68	0.0702	Not significant
1223	QTFYNRRYQPRPWTP	HLA-DRB1*08:01	7	21	4.81	Not predicted	162	KQKQQAPRN	HLA-DRB1*11:01	71	79	0.0654	Not significant
1224	TFYNRRYQPRPWTPR	HLA-DRB1*08:01	8	22	4.81	Not predicted	61	KPVQKKKKP	HLA-DRB1*11:01	91	99	0.0241	9.04
1225	FYNRRYQPRPWTPRP	HLA-DRB1*08:01	9	23	4.81	Not predicted	159	KPVQKKKKP	HLA-DRB1*07:01	91	99	0.0638	Not significant
1226	YNRRYQPRPWTPRPT	HLA-DRB1*08:01	10	24	4.81	Not predicted	206	KPVQKKKKP	HLA-DRB1*03:01	91	99	0.0976	7.82
680	RYQPRPWTPRPTIQV	HLA-DRB1*08:01	13	27	2.82	Not predicted	140	RRERMCMKI	HLA-DRB1*11:01	101	109	0.0559	Not significant
681	YQPRPWTPRPTIQVI	HLA-DRB1*08:01	14	28	2.82	Not predicted	68	RMCMKIEND	HLA-DRB1*03:01	104	112	0.0253	4.02

682	QPRPWTPRPTIQVIR	HLA-DRB1*08:01	15	29	2.82	Not predicted	121	CMKIENDCI	HLA-DRB1*01:01	106	114	0.0491	Not significant
683	PRPWTPRPTIQVIRP	HLA-DRB1*08:01	16	30	2.82	Not predicted	122	NDCIFEVKH	HLA-DRB1*11:01	111	119	0.0494	Not significant
684	RPWTPRPTIQVIRPR	HLA-DRB1*08:01	17	31	2.82	Not predicted	203	LVGDKVMKP	HLA-DRB1*11:01	129	137	0.0946	5.07
685	PWTPRPTIQVIRPRP	HLA-DRB1*08:01	18	32	2.82	Not predicted	127	KPAHVKGTI	HLA-DRB1*07:01	136	144	0.0509	Not significant
2204	PWTPRPTIQVIRPRP	HLA-DRB1*11:01	18	32	8.58	>0.1	193	KPAHVKGTI	HLA-DRB1*15:01	136	144	0.0892	Not significant
320	WTPRPTIQVIRPRPR	HLA-DRB1*13:01	19	33	1.35	>0.1	83	KGTIDNADL	HLA-DRB1*03:01	141	149	0.0297	Not significant
686	WTPRPTIQVIRPRPR	HLA-DRB1*08:01	19	33	2.82	Not predicted	98	LAKLAFKRS	HLA-DRB1*15:01	149	157	0.0369	Not significant
1355	WTPRPTIQVIRPRPR	HLA-DRB1*03:01	19	33	5.24	>0.1	36	KLAFKRSSK	HLA-DRB1*07:01	151	159	0.0137	2.55
1378	WTPRPTIQVIRPRPR	HLA-DRB1*11:01	19	33	5.43	>0.1	69	KLAFKRSSK	HLA-DRB1*11:01	151	159	0.0253	4.33
321	TPRPTIQVIRPRPRP	HLA-DRB1*13:01	20	34	1.35	>0.1	144	KLAFKRSSK	HLA-DRB1*03:01	151	159	0.0588	3.66

1356	TPRPTIQVIRPRPRP	HLA-DRB1*03:01	20	34	5.24	>0.1	154	SSKYDLECA	HLA-DRB1*01:01	157	165	0.0623	Not significant
1379	TPRPTIQVIRPRPRP	HLA-DRB1*11:01	20	34	5.43	>0.1	32	IPVHMKSDA	HLA-DRB1*03:01	167	175	0.0118	6.58
322	PRPTIQVIRPRPRPQ	HLA-DRB1*13:01	21	35	1.35	>0.1	134	IPVHMKSDA	HLA-DRB1*07:01	167	175	0.0529	Not significant
1357	PRPTIQVIRPRPRPQ	HLA-DRB1*03:01	21	35	5.24	>0.1	67	PEGYYNWHH	HLA-DRB1*11:01	183	191	0.0253	8.49
1380	PRPTIQVIRPRPRPQ	HLA-DRB1*11:01	21	35	5.43	>0.1	63	YYNWHHGAV	HLA-DRB1*07:01	186	194	0.0252	Not significant
1578	PRPTIQVIRPRPRPQ	HLA-DRB1*08:01	21	35	6.39	Not predicted	210	YYNWHHGAV	HLA-DRB1*01:01	186	194	0.0995	Not significant
147	RPTIQVIRPRPRPQR	HLA-DRB1*13:01	22	36	0.82	>0.1	5	KGRVVAIVL	HLA-DRB1*03:01	221	229	0.0027	Not significant
298	RPTIQVIRPRPRPQR	HLA-DRB1*03:01	22	36	1.28	0.0643	34	KGRVVAIVL	HLA-DRB1*15:01	221	229	0.013	Not significant
441	RPTIQVIRPRPRPQR	HLA-DRB1*08:01	22	36	1.96	Not predicted	106	KGRVVAIVL	HLA-DRB1*11:01	221	229	0.0417	7.45
1124	RPTIQVIRPRPRPQR	HLA-DRB1*15:01	22	36	4.53	>0.1	157	KGRVVAIVL	HLA-DRB1*07:01	221	229	0.0638	Not significant

1373	RPTIQVIRPRPRPQR	HLA-DRB1*11:01	22	36	5.4	0.0629	75	KDIVTKITP	HLA-DRB1*07:01	247	255	0.0275	Not significant
148	PTIQVIRPRPRPQRK	HLA-DRB1*13:01	23	37	0.82	>0.1	101	KDIVTKITP	HLA-DRB1*03:01	247	255	0.0383	Not significant
299	PTIQVIRPRPRPQRK	HLA-DRB1*03:01	23	37	1.28	>0.1	164	KDIVTKITP	HLA-DRB1*11:01	247	255	0.068	Not significant
442	PTIQVIRPRPRPQRK	HLA-DRB1*08:01	23	37	1.96	Not predicted	173	KDIVTKITP	HLA-DRB1*15:01	247	255	0.0759	Not significant
1219	PTIQVIRPRPRPQRK	HLA-DRB1*15:01	23	37	4.81	>0.1	185	EWSLAIPVM	HLA-DRB1*07:01	260	268	0.0845	Not significant
1374	PTIQVIRPRPRPQRK	HLA-DRB1*11:01	23	37	5.4	>0.1	142	AIPVMCLLA	HLA-DRB1*15:01	264	272	0.0577	6.96
149	TIQVIRPRPRPQRKA	HLA-DRB1*13:01	24	38	0.82	>0.1	17	IPVMCLLAN	HLA-DRB1*03:01	265	273	0.00749	Not significant
443	TIQVIRPRPRPQRKA	HLA-DRB1*08:01	24	38	1.96	Not predicted	149	IPVMCLLAN	HLA-DRB1*11:01	265	273	0.0605	6.64
510	TIQVIRPRPRPQRKA	HLA-DRB1*03:01	24	38	2.27	>0.1	8	VMCLLANTT	HLA-DRB1*01:01	267	275	0.00416	Not significant
1375	TIQVIRPRPRPQRKA	HLA-DRB1*11:01	24	38	5.4	>0.1	186	NTTFPCSRP	HLA-DRB1*07:01	273	281	0.0845	Not significant

150	IQVIRPRPRPQRKAG	HLA-DRB1*13:01	25	39	0.82	>0.1	207	KEPEKTLRM	HLA-DRB1*15:01	290	298	0.0979	Not significant
444	IQVIRPRPRPQRKAG	HLA-DRB1*08:01	25	39	1.96	Not predicted	54	TLRMLEDNV	HLA-DRB1*03:01	295	303	0.0214	0.93
514	IQVIRPRPRPQRKAG	HLA-DRB1*03:01	25	39	2.31	>0.1	118	PGYYQLLQA	HLA-DRB1*07:01	306	314	0.0471	Not significant
1376	IQVIRPRPRPQRKAG	HLA-DRB1*11:01	25	39	5.4	>0.1	136	PGYYQLLQA	HLA-DRB1*15:01	306	314	0.0537	8.57
151	QVIRPRPRPQRKAGQ	HLA-DRB1*13:01	26	40	0.82	>0.1	3	GYYQLLQAS	HLA-DRB1*11:01	307	315	0.00134	4.37
445	QVIRPRPRPQRKAGQ	HLA-DRB1*08:01	26	40	1.96	Not predicted	95	GYYQLLQAS	HLA-DRB1*03:01	307	315	0.0365	8.06
1377	QVIRPRPRPQRKAGQ	HLA-DRB1*11:01	26	40	5.4	>0.1	53	YYQLLQASL	HLA-DRB1*01:01	308	316	0.0208	0.28
1538	QVIRPRPRPQRKAGQ	HLA-DRB1*03:01	26	40	6.2	>0.1	170	RRSIKDHFN	HLA-DRB1*15:01	324	332	0.0734	Not significant
152	VIRPRPRPQRKAGQL	HLA-DRB1*13:01	27	41	0.82	>0.1	91	KDHFNVYKA	HLA-DRB1*01:01	328	336	0.0333	4.36
446	VIRPRPRPQRKAGQL	HLA-DRB1*08:01	27	41	1.96	Not predicted	29	DHFNVYKAT	HLA-DRB1*07:01	329	337	0.0101	1.83

1490	VIRPRPRPQRKAGQL	HLA-DRB1*11:01	27	41	5.97	>0.1	44	HFNVYKATR	HLA-DRB1*03:01	330	338	0.017	4.35
153	IRPRPRPQRKAGQLA	HLA-DRB1*13:01	28	42	0.82	>0.1	58	FNVYKATRP	HLA-DRB1*11:01	331	339	0.0229	1.88
447	IRPRPRPQRKAGQLA	HLA-DRB1*08:01	28	42	1.96	Not predicted	152	DGTLKIQVS	HLA-DRB1*03:01	368	376	0.0615	1.99
1758	QRKAGQLAQLISAVN	HLA-DRB1*01:01	35	49	6.9	>0.1	182	DGTLKIQVS	HLA-DRB1*07:01	368	376	0.0817	2.03
1759	RKAGQLAQLISAVNK	HLA-DRB1*01:01	36	50	6.9	>0.1	188	LKIQVSLQI	HLA-DRB1*15:01	371	379	0.0864	5.14
1684	KAGQLAQLISAVNKL	HLA-DRB1*04:01	37	51	6.67	>0.1	80	QIGIKTDDS	HLA-DRB1*03:01	378	386	0.0281	9.61
1760	KAGQLAQLISAVNKL	HLA-DRB1*01:01	37	51	6.9	>0.1	79	WTKLRYMDN	HLA-DRB1*15:01	389	397	0.0278	8.88
1418	AGQLAQLISAVNKLT	HLA-DRB1*01:01	38	52	5.5	>0.1	22	KLRYMDNHM	HLA-DRB1*03:01	391	399	0.00856	Not significant
1441	AGQLAQLISAVNKLT	HLA-DRB1*04:01	38	52	5.58	>0.1	62	KLRYMDNHM	HLA-DRB1*01:01	391	399	0.0252	Not significant
2521	AGQLAQLISAVNKLT	HLA-DRB1*11:01	38	52	9.92	>0.1	81	KLRYMDNHM	HLA-DRB1*07:01	391	399	0.0287	Not significant

703	GQLAQLISAVNKLTM	HLA-DRB1*11:01	39	53	2.9	>0.1	131	KLRYMDNHM	HLA-DRB1*11:01	391	399	0.0515	9.74
1358	GQLAQLISAVNKLTM	HLA-DRB1*03:01	39	53	5.24	>0.1	99	RAGLFVRTS	HLA-DRB1*07:01	405	413	0.037	1.5
1761	GQLAQLISAVNKLTM	HLA-DRB1*01:01	39	53	6.9	>0.1	137	RAGLFVRTS	HLA-DRB1*15:01	405	413	0.0537	9.19
1765	GQLAQLISAVNKLTM	HLA-DRB1*13:01	39	53	6.91	>0.1	117	AGLFVRTSA	HLA-DRB1*03:01	406	414	0.0466	3.42
1810	GQLAQLISAVNKLTM	HLA-DRB1*04:01	39	53	6.98	>0.1	125	GLFVRTSAP	HLA-DRB1*11:01	407	415	0.0494	4.68
1982	GQLAQLISAVNKLTM	HLA-DRB1*08:01	39	53	7.82	Not predicted	172	GLFVRTSAP	HLA-DRB1*01:01	407	415	0.0751	4.99
704	QLAQLISAVNKLTMR	HLA-DRB1*11:01	40	54	2.9	>0.1	57	TMGHFILAR	HLA-DRB1*15:01	421	429	0.0225	Not significant
1359	QLAQLISAVNKLTMR	HLA-DRB1*03:01	40	54	5.24	>0.1	141	TMGHFILAR	HLA-DRB1*03:01	421	429	0.0562	5.69
1762	QLAQLISAVNKLTMR	HLA-DRB1*01:01	40	54	6.9	>0.1	27	GHFILARCP	HLA-DRB1*11:01	423	431	0.00936	3.8
1766	QLAQLISAVNKLTMR	HLA-DRB1*13:01	40	54	6.91	>0.1	82	GHFILARCP	HLA-DRB1*01:01	423	431	0.0291	Not significant

1983	QLAQLISAVNKLTMR	HLA-DRB1*08:01	40	54	7.82	Not predicted	158	GHFILARCP	HLA-DRB1*07:01	423	431	0.0638	Not significant
2152	QLAQLISAVNKLTMR	HLA-DRB1*04:01	40	54	8.45	>0.1	150	RKISHSCTH	HLA-DRB1*07:01	444	452	0.0615	3.75
545	LAQLISAVNKLTMRV	HLA-DRB1*11:01	41	55	2.39	>0.1	187	RKISHSCTH	HLA-DRB1*11:01	444	452	0.085	Not significant
1360	LAQLISAVNKLTMRV	HLA-DRB1*03:01	41	55	5.24	>0.1	196	GREKFHSRP	HLA-DRB1*11:01	462	470	0.0913	Not significant
1767	LAQLISAVNKLTMRV	HLA-DRB1*13:01	41	55	6.91	>0.1	211	REKFHSRPQ	HLA-DRB1*07:01	463	471	0.0998	Not significant
1984	LAQLISAVNKLTMRV	HLA-DRB1*08:01	41	55	7.82	Not predicted	130	**CSTYAQSTA**	**HLA-DRB1*01:01**	**478**	**486**	**0.0511**	**6.21**
2153	LAQLISAVNKLTMRV	HLA-DRB1*04:01	41	55	8.45	>0.1	146	CSTYAQSTA	HLA-DRB1*07:01	478	486	0.0592	Not significant
2373	LAQLISAVNKLTMRV	HLA-DRB1*01:01	41	55	9.3	>0.1	78	EEIEVHMPP	HLA-DRB1*15:01	490	498	0.0278	Not significant
705	AQLISAVNKLTMRVV	HLA-DRB1*11:01	42	56	2.9	>0.1	113	SGNVKITVN	HLA-DRB1*03:01	510	518	0.0444	3.62
1361	AQLISAVNKLTMRVV	HLA-DRB1*03:01	42	56	5.24	0.0588	192	SGNVKITVN	HLA-DRB1*11:01	510	518	0.0881	Not significant

1475	AQLISAVNKLTMRVV	HLA-DRB1*13:01	42	56	5.9	>0.1	70	KITVNSQTV	HLA-DRB1*07:01	514	522	0.0263	Not significant
1985	AQLISAVNKLTMRVV	HLA-DRB1*08:01	42	56	7.82	Not predicted	190	KITVNSQTV	HLA-DRB1*01:01	514	522	0.0867	Not significant
2154	AQLISAVNKLTMRVV	HLA-DRB1*04:01	42	56	8.45	>0.1	194	KVINNCKVD	HLA-DRB1*03:01	540	548	0.0901	8.67
706	QLISAVNKLTMRVVP	HLA-DRB1*11:01	43	57	2.9	>0.1	76	NCKVDQCHA	HLA-DRB1*01:01	544	552	0.0277	Not significant
1362	QLISAVNKLTMRVVP	HLA-DRB1*03:01	43	57	5.24	>0.1	195	NCKVDQCHA	HLA-DRB1*07:01	544	552	0.0904	Not significant
1476	QLISAVNKLTMRVVP	HLA-DRB1*13:01	43	57	5.9	>0.1	180	HKKWQYNSP	HLA-DRB1*15:01	557	565	0.081	Not significant
1986	QLISAVNKLTMRVVP	HLA-DRB1*08:01	43	57	7.82	Not predicted	71	KWQYNSPLV	HLA-DRB1*07:01	559	567	0.0263	Not significant
2155	QLISAVNKLTMRVVP	HLA-DRB1*04:01	43	57	8.45	>0.1	77	KWQYNSPLV	HLA-DRB1*01:01	559	567	0.0277	Not significant
707	LISAVNKLTMRVVPQ	HLA-DRB1*11:01	44	58	2.9	>0.1	94	SPLVPRNAE	HLA-DRB1*03:01	564	572	0.0347	Not significant
1363	LISAVNKLTMRVVPQ	HLA-DRB1*03:01	44	58	5.24	>0.1	39	KGKVHIPFP	HLA-DRB1*15:01	577	585	0.0157	Not significant

1477	LISAVNKLTMRVVPQ	HLA-DRB1*13:01	44	58	5.9	>0.1	51	KGKVHIPFP	HLA-DRB1*07:01	577	585	0.0192	Not significant
1987	LISAVNKLTMRVVPQ	HLA-DRB1*08:01	44	58	7.82	Not predicted	52	KGKVHIPFP	HLA-DRB1*11:01	577	585	0.0198	Not significant
2156	LISAVNKLTMRVVPQ	HLA-DRB1*04:01	44	58	8.45	>0.1	174	KGKVHIPFP	HLA-DRB1*03:01	577	585	0.0764	Not significant
60	ISAVNKLTMRVVPQQ	HLA-DRB1*08:01	45	59	0.43	Not predicted	21	KNQVIMLLY	HLA-DRB1*15:01	605	613	0.00835	4.48
708	ISAVNKLTMRVVPQQ	HLA-DRB1*11:01	45	59	2.9	>0.1	15	NQVIMLLYP	HLA-DRB1*11:01	606	614	0.007	3.72
1269	ISAVNKLTMRVVPQQ	HLA-DRB1*13:01	45	59	4.98	>0.1	43	NQVIMLLYP	HLA-DRB1*03:01	606	614	0.017	1.12
61	SAVNKLTMRVVPQQK	HLA-DRB1*08:01	46	60	0.43	Not predicted	45	NQVIMLLYP	HLA-DRB1*01:01	606	614	0.0178	Not significant
851	SAVNKLTMRVVPQQK	HLA-DRB1*11:01	46	60	3.42	>0.1	88	NQVIMLLYP	HLA-DRB1*07:01	606	614	0.0313	9.19
1270	SAVNKLTMRVVPQQK	HLA-DRB1*13:01	46	60	4.98	>0.1	87	HPTLLSYRN	HLA-DRB1*11:01	616	624	0.0305	Not significant
62	AVNKLTMRVVPQQKP	HLA-DRB1*08:01	47	61	0.43	Not predicted	126	PTLLSYRNM	HLA-DRB1*15:01	617	625	0.05	Not significant

852	AVNKLTMRVVPQQKP	HLA-DRB1*11:01	47	61	3.42	0.0366
1271	AVNKLTMRVVPQQKP	HLA-DRB1*13:01	47	61	4.98	>0.1
63	VNKLTMRVVPQQKPR	HLA-DRB1*08:01	48	62	0.43	Not predicted
853	VNKLTMRVVPQQKPR	HLA-DRB1*11:01	48	62	3.42	>0.1
1272	VNKLTMRVVPQQKPR	HLA-DRB1*13:01	48	62	4.98	>0.1
64	NKLTMRVVPQQKPRK	HLA-DRB1*08:01	49	63	0.43	Not predicted
217	NKLTMRVVPQQKPRK	HLA-DRB1*13:01	49	63	1.05	>0.1
1191	NKLTMRVVPQQKPRK	HLA-DRB1*11:01	49	63	4.71	>0.1
2195	NKLTMRVVPQQKPRK	HLA-DRB1*03:01	49	63	8.54	>0.1
65	KLTMRVVPQQKPRKN	HLA-DRB1*08:01	50	64	0.43	Not predicted

204	PTLLSYRNM	HLA-DRB1*01:01	617	625	0.0962	Not significant
**177**	**WVTHKKEIR**	**HLA-DRB1*03:01**	**635**	**643**	**0.0797**	**3.67**
176	KKEIRLTVP	HLA-DRB1*11:01	639	647	0.079	9.53
167	EPYKYWPQL	HLA-DRB1*07:01	659	667	0.071	Not significant
191	EPYKYWPQL	HLA-DRB1*11:01	659	667	0.0881	2.21
40	PYKYWPQLS	HLA-DRB1*01:01	660	668	0.0161	Not significant
2	EIILYYYEL	HLA-DRB1*15:01	679	687	0.000326	1.75
46	ILYYYELYP	HLA-DRB1*11:01	681	689	0.0179	1.08
64	LYYYELYPT	HLA-DRB1*01:01	682	690	0.0252	1.15
7	YYYELYPTM	HLA-DRB1*07:01	683	691	0.004	5.97

218	KLTMRVVPQQKPRKN	HLA-DRB1*13:01	50	64	1.05	>0.1	14	VASFILLSM	HLA-DRB1*01:01	698	706	0.0069	0.28
993	KLTMRVVPQQKPRKN	HLA-DRB1*11:01	50	64	4.04	>0.1	41	VASFILLSM	HLA-DRB1*15:01	698	706	0.0165	1.5
2205	KLTMRVVPQQKPRKN	HLA-DRB1*03:01	50	64	8.59	>0.1	16	ASFILLSMV	HLA-DRB1*07:01	699	707	0.00739	Not significant
66	LTMRVVPQQKPRKNR	HLA-DRB1*08:01	51	65	0.43	Not predicted	38	ASFILLSMV	HLA-DRB1*11:01	699	707	0.0153	4.02
219	LTMRVVPQQKPRKNR	HLA-DRB1*13:01	51	65	1.05	>0.1	35	SFILLSMVG	HLA-DRB1*03:01	700	708	0.0134	0.89
972	LTMRVVPQQKPRKNR	HLA-DRB1*11:01	51	65	3.89	>0.1	97	GMCMCARRR	HLA-DRB1*11:01	712	720	0.0366	4.66
1909	LTMRVVPQQKPRKNR	HLA-DRB1*03:01	51	65	7.49	>0.1	179	GMCMCARRR	HLA-DRB1*01:01	712	720	0.0808	Not significant
220	TMRVVPQQKPRKNRK	HLA-DRB1*13:01	52	66	1.05	>0.1	33	TVPFLLSLI	HLA-DRB1*15:01	732	740	0.0124	Not significant
1368	TMRVVPQQKPRKNRK	HLA-DRB1*11:01	52	66	5.35	>0.1	28	VPFLLSLIC	HLA-DRB1*01:01	733	741	0.00983	Not significant
1840	TMRVVPQQKPRKNRK	HLA-DRB1*03:01	52	66	7.32	>0.1	93	FLLSLICCI	HLA-DRB1*07:01	735	743	0.034	Not significant

221	MRVVPQQKPRKNRKN	HLA-DRB1*13:01	53	67	1.05	>0.1	124	LSLICCIRT	HLA-DRB1*11:01	737	745	0.0494	5.43
1266	MRVVPQQKPRKNRKN	HLA-DRB1*11:01	53	67	4.96	>0.1	201	LICCIRTAK	HLA-DRB1*03:01	739	747	0.0938	4.37
1911	MRVVPQQKPRKNRKN	HLA-DRB1*03:01	53	67	7.5	>0.1	133	QEAAVYLWN	HLA-DRB1*15:01	752	760	0.0518	Not significant
222	RVVPQQKPRKNRKNK	HLA-DRB1*13:01	54	68	1.05	>0.1	47	AAVYLWNEQ	HLA-DRB1*03:01	754	762	0.018	3.9
1938	RVVPQQKPRKNRKNK	HLA-DRB1*11:01	54	68	7.6	>0.1	48	**QPLFWMQAL**	**HLA-DRB1*03:01**	**763**	**771**	**0.018**	**7.21**
223	VVPQQKPRKNRKNKK	HLA-DRB1*13:01	55	69	1.05	>0.1	11	PLFWMQALI	HLA-DRB1*07:01	764	772	0.00532	Not significant
512	VVPQQKPRKNRKNKK	HLA-DRB1*11:01	55	69	2.29	>0.1	56	PLFWMQALI	HLA-DRB1*15:01	764	772	0.0215	Not significant
1685	VVPQQKPRKNRKNKK	HLA-DRB1*03:01	55	69	6.68	>0.1	4	**LFWMQALIP**	**HLA-DRB1*01:01**	**765**	**773**	**0.00195**	**0.6**
2477	VPQQKPRKNRKNKKQ	HLA-DRB1*13:01	56	70	9.66	>0.1	73	**FWMQALIPL**	**HLA-DRB1*11:01**	**766**	**774**	**0.0265**	**0.43**
323	PPKKKPVQKKKKPGR	HLA-DRB1*13:01	87	101	1.35	>0.1	74	ALIVLCNCL	HLA-DRB1*15:01	776	784	0.0267	Not significant

1097	PPKKKPVQKKKKPGR	HLA-DRB1*08:01	87	101	4.47	Not predicted	112	ALIVLCNCL	HLA-DRB1*03:01	776	784	0.0444	2.19
2088	PPKKKPVQKKKKPGR	HLA-DRB1*03:01	87	101	8.07	>0.1	120	ALIVLCNCL	HLA-DRB1*07:01	776	784	0.049	Not significant
2297	PPKKKPVQKKKKPGR	HLA-DRB1*11:01	87	101	9.04	>0.1	107	VLCNCLRLL	HLA-DRB1*11:01	779	787	0.0417	5.25
324	PKKKPVQKKKKPGRR	HLA-DRB1*13:01	88	102	1.35	>0.1	1	CCKMLTFLA	HLA-DRB1*01:01	790	798	0.0000264	2.37
1098	PKKKPVQKKKKPGRR	HLA-DRB1*08:01	88	102	4.47	Not predicted	85	CCKMLTFLA	HLA-DRB1*15:01	790	798	0.0302	2.37
2008	PKKKPVQKKKKPGRR	HLA-DRB1*03:01	88	102	7.87	>0.1	13	KMLTFLAVL	HLA-DRB1*11:01	792	800	0.00659	0.7
2298	PKKKPVQKKKKPGRR	HLA-DRB1*11:01	88	102	9.04	>0.1	9	MLTFLAVLS	HLA-DRB1*07:01	793	801	0.00475	3.22
325	KKKPVQKKKKPGRRE	HLA-DRB1*13:01	89	103	1.35	>0.1	37	MLTFLAVLS	HLA-DRB1*03:01	793	801	0.0151	Not significant
1099	KKKPVQKKKKPGRRE	HLA-DRB1*08:01	89	103	4.47	Not predicted	166	SAYEHVTVI	HLA-DRB1*07:01	808	816	0.071	Not significant
1961	KKKPVQKKKKPGRRE	HLA-DRB1*03:01	89	103	7.81	>0.1	123	**PYKTLVNRP**	**HLA-DRB1*11:01**	**823**	**831**	**0.0494**	**6.04**

2299	KKKPVQKKKKPGRRE	HLA-DRB1*11:01	89	103	9.04	>0.1	30	**SPMVLEMEL**	**HLA-DRB1*03:01**	**834**	**842**	**0.0111**	**6.71**
326	KKPVQKKKKPGRRER	HLA-DRB1*13:01	90	104	1.35	>0.1	92	SPMVLEMEL	HLA-DRB1*01:01	834	842	0.0333	Not significant
1100	KKPVQKKKKPGRRER	HLA-DRB1*08:01	90	104	4.47	Not predicted	108	EMELLSVTL	HLA-DRB1*07:01	839	847	0.0418	Not significant
1946	KKPVQKKKKPGRRER	HLA-DRB1*03:01	90	104	7.66	>0.1	119	EMELLSVTL	HLA-DRB1*11:01	839	847	0.0474	Not significant
2300	KKPVQKKKKPGRRER	HLA-DRB1*11:01	90	104	9.04	>0.1	183	EPTLSLDYI	HLA-DRB1*03:01	848	856	0.0831	2.48
327	KPVQKKKKPGRRERM	HLA-DRB1*13:01	91	105	1.35	>0.1	105	SPYVKCCGT	HLA-DRB1*01:01	866	874	0.0415	Not significant
1101	KPVQKKKKPGRRERM	HLA-DRB1*08:01	91	105	4.47	Not predicted	181	SPYVKCCGT	HLA-DRB1*07:01	866	874	0.0817	Not significant
1980	KPVQKKKKPGRRERM	HLA-DRB1*03:01	91	105	7.82	0.0976	129	SCKVFTGVY	HLA-DRB1*01:01	886	894	0.0511	Not significant
2301	KPVQKKKKPGRRERM	HLA-DRB1*11:01	91	105	9.04	0.0241	171	SCKVFTGVY	HLA-DRB1*07:01	886	894	0.0736	Not significant
328	PVQKKKKPGRRERMC	HLA-DRB1*13:01	92	106	1.35	>0.1	132	TGVYPFMWG	HLA-DRB1*15:01	891	899	0.0518	Not significant

1102	PVQKKKKPGRRERMC	HLA-DRB1*08:01	92	106	4.47	Not predicted	198	YCFCDTENT	HLA-DRB1*01:01	902	910	0.0929	Not significant
2302	PVQKKKKPGRRERMC	HLA-DRB1*11:01	92	106	9.04	>0.1	156	CKTEFASAY	HLA-DRB1*07:01	923	931	0.0638	Not significant
329	VQKKKKPGRRERMCM	HLA-DRB1*13:01	93	107	1.35	>0.1	**169**	**KTEFASAYR**	**HLA-DRB1*11:01**	**924**	**932**	**0.0734**	**1.46**
1103	VQKKKKPGRRERMCM	HLA-DRB1*08:01	93	107	4.47	Not predicted	19	KLRVLYQGN	HLA-DRB1*15:01	941	949	0.00793	2.37
1000	ERMCMKIENDCIFEV	HLA-DRB1*03:01	103	117	4.12	>0.1	55	KLRVLYQGN	HLA-DRB1*03:01	941	949	0.0214	Not significant
987	RMCMKIENDCIFEVK	HLA-DRB1*03:01	104	118	4.02	0.0253	128	KLRVLYQGN	HLA-DRB1*07:01	941	949	0.0509	Not significant
996	MCMKIENDCIFEVKH	HLA-DRB1*03:01	105	119	4.07	>0.1	163	NITVAAYAN	HLA-DRB1*11:01	950	958	0.068	Not significant
1473	CMKIENDCIFEVKHE	HLA-DRB1*03:01	106	120	5.88	>0.1	18	HAVTVKDAK	HLA-DRB1*03:01	961	969	0.00749	4.74
1460	MKIENDCIFEVKHEG	HLA-DRB1*03:01	107	121	5.74	>0.1	199	KDAKFIVGP	HLA-DRB1*07:01	966	974	0.0935	Not significant
2108	IENDCIFEVKHEGKV	HLA-DRB1*08:01	109	123	8.32	Not predicted	102	KFIVGPMSS	HLA-DRB1*15:01	969	977	0.0384	7.16

2109	ENDCIFEVKHEGKVT	HLA-DRB1*08:01	110	124	8.32	Not predicted	175	KFIVGPMSS	HLA-DRB1*01:01	969	977	0.0779	Not significant
1564	NDCIFEVKHEGKVTG	HLA-DRB1*13:01	111	125	6.36	>0.1	151	TPFDNKIVV	HLA-DRB1*07:01	980	988	0.0615	Not significant
2110	NDCIFEVKHEGKVTG	HLA-DRB1*08:01	111	125	8.32	Not predicted	90	DNKIVVYKG	HLA-DRB1*15:01	983	991	0.0327	2.44
1565	DCIFEVKHEGKVTGY	HLA-DRB1*13:01	112	126	6.36	>0.1	25	KIVVYKGDV	HLA-DRB1*03:01	985	993	0.00915	7.94
2111	DCIFEVKHEGKVTGY	HLA-DRB1*08:01	112	126	8.32	Not predicted	208	FGDIQSRTP	HLA-DRB1*11:01	1009	1017	0.0979	Not significant
1566	CIFEVKHEGKVTGYA	HLA-DRB1*13:01	113	127	6.36	>0.1	189	SEDVYANTQ	HLA-DRB1*03:01	1019	1027	0.0865	9.54
2112	CIFEVKHEGKVTGYA	HLA-DRB1*08:01	113	127	8.32	Not predicted	139	NTQLVLQRP	HLA-DRB1*15:01	1025	1033	0.0557	Not significant
1567	IFEVKHEGKVTGYAC	HLA-DRB1*13:01	114	128	6.36	>0.1	49	PSGFKYWLK	HLA-DRB1*15:01	1046	1054	0.018	Not significant
2113	IFEVKHEGKVTGYAC	HLA-DRB1*08:01	114	128	8.32	Not predicted	103	PSGFKYWLK	HLA-DRB1*07:01	1046	1054	0.0401	5.02
1568	FEVKHEGKVTGYACL	HLA-DRB1*13:01	115	129	6.36	>0.1	135	GFKYWLKER	HLA-DRB1*03:01	1048	1056	0.0537	Not significant

2114	FEVKHEGKVTGYACL	HLA-DRB1*08:01	115	129	8.32	Not predicted	148	GFKYWLKER	HLA-DRB1*01:01	1048	1056	0.0599	8.28
1569	EVKHEGKVTGYACLV	HLA-DRB1*13:01	116	130	6.36	>0.1	12	FKYWLKERG	HLA-DRB1*11:01	1049	1057	0.00621	2.63
1227	VKHEGKVTGYACLVG	HLA-DRB1*08:01	117	131	4.81	Not predicted	200	PFGCQIATN	HLA-DRB1*07:01	1065	1073	0.0935	Not significant
1570	VKHEGKVTGYACLVG	HLA-DRB1*13:01	117	131	6.36	>0.1	202	FGCQIATNP	HLA-DRB1*11:01	1066	1074	0.0946	Not significant
1228	KHEGKVTGYACLVGD	HLA-DRB1*08:01	118	132	4.81	Not predicted	197	TNPVRAMNC	HLA-DRB1*15:01	1072	1080	0.092	Not significant
2025	KHEGKVTGYACLVGD	HLA-DRB1*13:01	118	132	7.95	>0.1	184	NCAVGNMPI	HLA-DRB1*01:01	1079	1087	0.0837	Not significant
1229	HEGKVTGYACLVGDK	HLA-DRB1*08:01	119	133	4.81	Not predicted	168	PISIDIPDA	HLA-DRB1*03:01	1086	1094	0.0732	1.77
2026	HEGKVTGYACLVGDK	HLA-DRB1*13:01	119	133	7.95	>0.1	209	SCEVSACTH	HLA-DRB1*01:01	1109	1117	0.0995	Not significant
1230	EGKVTGYACLVGDKV	HLA-DRB1*08:01	120	134	4.81	Not predicted	60	GVAIIKYAA	HLA-DRB1*11:01	1123	1131	0.0241	5.43
2027	EGKVTGYACLVGDKV	HLA-DRB1*13:01	120	134	7.95	>0.1	153	GVAIIKYAA	HLA-DRB1*01:01	1123	1131	0.0623	Not significant

1231	GKVTGYACLVGDKVM	HLA-DRB1*08:01	121	135	4.81	Not predicted	10	VAIIKYAAS	HLA-DRB1*15:01	1124	1132	0.00491	2.71
2028	GKVTGYACLVGDKVM	HLA-DRB1*13:01	121	135	7.95	>0.1	59	VAIIKYAAS	HLA-DRB1*03:01	1124	1132	0.0239	8.34
1232	KVTGYACLVGDKVMK	HLA-DRB1*08:01	122	136	4.81	Not predicted	66	VAIIKYAAS	HLA-DRB1*07:01	1124	1132	0.0252	9.21
2029	KVTGYACLVGDKVMK	HLA-DRB1*13:01	122	136	7.95	>0.1	23	KCAVHSMTN	HLA-DRB1*07:01	1136	1144	0.00867	5.61
119	VTGYACLVGDKVMKP	HLA-DRB1*03:01	123	137	0.69	>0.1	26	KCAVHSMTN	HLA-DRB1*11:01	1136	1144	0.00936	Not significant
1233	VTGYACLVGDKVMKP	HLA-DRB1*08:01	123	137	4.81	Not predicted	31	KCAVHSMTN	HLA-DRB1*03:01	1136	1144	0.0118	Not significant
1298	VTGYACLVGDKVMKP	HLA-DRB1*11:01	123	137	5.07	>0.1	100	KCAVHSMTN	HLA-DRB1*01:01	1136	1144	0.0381	3.45
1478	VTGYACLVGDKVMKP	HLA-DRB1*13:01	123	137	5.9	>0.1	116	KCAVHSMTN	HLA-DRB1*15:01	1136	1144	0.0464	7.71
2355	VTGYACLVGDKVMKP	HLA-DRB1*04:01	123	137	9.1	>0.1	84	NSQLQISFS	HLA-DRB1*15:01	1158	1166	0.0302	6.64
93	TGYACLVGDKVMKPA	HLA-DRB1*03:01	124	138	0.54	>0.1	86	NSQLQISFS	HLA-DRB1*01:01	1158	1166	0.0304	8.36

1299	TGYACLVGDKVMKPA	HLA-DRB1*11:01	124	138	5.07	>0.1	178	NSQLQISFS	HLA-DRB1*03:01	1158	1166	0.0797	2.42
1479	TGYACLVGDKVMKPA	HLA-DRB1*13:01	124	138	5.9	>0.1	20	QLQISFSTA	HLA-DRB1*07:01	1160	1168	0.00823	1.98
2263	TGYACLVGDKVMKPA	HLA-DRB1*04:01	124	138	8.95	>0.1	72	LQISFSTAL	HLA-DRB1*11:01	1161	1169	0.0265	4.33
53	GYACLVGDKVMKPAH	HLA-DRB1*03:01	125	139	0.29	>0.1	143	FRVQVCSTQ	HLA-DRB1*03:01	1174	1182	0.0588	Not significant
1300	GYACLVGDKVMKPAH	HLA-DRB1*11:01	125	139	5.07	>0.1	110	PKDHIVNYP	HLA-DRB1*15:01	1192	1200	0.043	1.4
1480	GYACLVGDKVMKPAH	HLA-DRB1*13:01	125	139	5.9	>0.1	138	KDHIVNYPA	HLA-DRB1*01:01	1193	1201	0.0554	Not significant
17	YACLVGDKVMKPAHV	HLA-DRB1*03:01	126	140	0.17	>0.1	111	DHIVNYPAS	HLA-DRB1*07:01	1194	1202	0.0435	Not significant
1301	YACLVGDKVMKPAHV	HLA-DRB1*11:01	126	140	5.07	>0.1	161	LGVQDISAT	HLA-DRB1*03:01	1206	1214	0.0643	Not significant
1481	YACLVGDKVMKPAHV	HLA-DRB1*13:01	126	140	5.9	>0.1	114	MSWVQKITG	HLA-DRB1*01:01	1216	1224	0.0452	Not significant
1641	YACLVGDKVMKPAHV	HLA-DRB1*15:01	126	140	6.54	>0.1	24	**AALILIVVL**	**HLA-DRB1*15:01**	**1233**	**1241**	**0.00879**	**1.77**

2216	YACLVGDKVMKPAHV	HLA-DRB1*04:01	126	140	8.73	>0.1
55	ACLVGDKVMKPAHVK	HLA-DRB1*03:01	127	141	0.4	>0.1
1302	ACLVGDKVMKPAHVK	HLA-DRB1*11:01	127	141	5.07	>0.1
1482	ACLVGDKVMKPAHVK	HLA-DRB1*13:01	127	141	5.9	>0.1
1642	ACLVGDKVMKPAHVK	HLA-DRB1*15:01	127	141	6.54	>0.1
2261	ACLVGDKVMKPAHVK	HLA-DRB1*04:01	127	141	8.94	>0.1
191	CLVGDKVMKPAHVKG	HLA-DRB1*03:01	128	142	0.92	>0.1
1303	CLVGDKVMKPAHVKG	HLA-DRB1*11:01	128	142	5.07	>0.1
1483	CLVGDKVMKPAHVKG	HLA-DRB1*13:01	128	142	5.9	>0.1
1643	CLVGDKVMKPAHVKG	HLA-DRB1*15:01	128	142	6.54	>0.1

89	**AALILIVVL**	**HLA-DRB1*11:01**	**1233**	**1241**	**0.0319**	**0.4**
42	LILIVVLCV	HLA-DRB1*01:01	1235	1243	0.0169	Not significant
6	ILIVVLCVS	HLA-DRB1*03:01	1236	1244	0.00341	Not significant
50	ILIVVLCVS	HLA-DRB1*07:01	1236	1244	0.0183	Not significant

595	LVGDKVMKPAHVKGT	HLA-DRB1*03:01	129	143	2.57	>0.1
1133	LVGDKVMKPAHVKGT	HLA-DRB1*13:01	129	143	4.62	>0.1
1304	LVGDKVMKPAHVKGT	HLA-DRB1*11:01	129	143	5.07	0.0946
1644	LVGDKVMKPAHVKGT	HLA-DRB1*15:01	129	143	6.54	>0.1
2234	LVGDKVMKPAHVKGT	HLA-DRB1*08:01	129	143	8.83	Not predicted
1134	VGDKVMKPAHVKGTI	HLA-DRB1*13:01	130	144	4.62	>0.1
1919	VGDKVMKPAHVKGTI	HLA-DRB1*11:01	130	144	7.59	>0.1
2235	VGDKVMKPAHVKGTI	HLA-DRB1*08:01	130	144	8.83	Not predicted
1135	GDKVMKPAHVKGTID	HLA-DRB1*13:01	131	145	4.62	>0.1
1920	GDKVMKPAHVKGTID	HLA-DRB1*11:01	131	145	7.59	>0.1

2236	GDKVMKPAHVKGTID	HLA-DRB1*08:01	131	145	8.83	Not predicted
1136	DKVMKPAHVKGTIDN	HLA-DRB1*13:01	132	146	4.62	>0.1
1921	DKVMKPAHVKGTIDN	HLA-DRB1*11:01	132	146	7.59	>0.1
2237	DKVMKPAHVKGTIDN	HLA-DRB1*08:01	132	146	8.83	Not predicted
1137	KVMKPAHVKGTIDNA	HLA-DRB1*13:01	133	147	4.62	>0.1
2238	KVMKPAHVKGTIDNA	HLA-DRB1*08:01	133	147	8.83	Not predicted
1138	VMKPAHVKGTIDNAD	HLA-DRB1*13:01	134	148	4.62	>0.1
2239	VMKPAHVKGTIDNAD	HLA-DRB1*08:01	134	148	8.83	Not predicted
1139	MKPAHVKGTIDNADL	HLA-DRB1*13:01	135	149	4.62	>0.1
2240	MKPAHVKGTIDNADL	HLA-DRB1*08:01	135	149	8.83	Not predicted

1918	IDNADLAKLAFKRSS	HLA-DRB1*11:01	144	158	7.56	>0.1
997	DNADLAKLAFKRSSK	HLA-DRB1*11:01	145	159	4.07	>0.1
506	NADLAKLAFKRSSKY	HLA-DRB1*11:01	146	160	2.26	>0.1
1659	NADLAKLAFKRSSKY	HLA-DRB1*07:01	146	160	6.59	>0.1
1962	NADLAKLAFKRSSKY	HLA-DRB1*08:01	146	160	7.81	Not predicted
600	ADLAKLAFKRSSKYD	HLA-DRB1*11:01	147	161	2.57	>0.1
1699	ADLAKLAFKRSSKYD	HLA-DRB1*07:01	147	161	6.73	>0.1
1963	ADLAKLAFKRSSKYD	HLA-DRB1*08:01	147	161	7.81	Not predicted
517	DLAKLAFKRSSKYDL	HLA-DRB1*07:01	148	162	2.32	>0.1
849	DLAKLAFKRSSKYDL	HLA-DRB1*11:01	148	162	3.41	>0.1

897	DLAKLAFKRSSKYDL	HLA-DRB1*03:01	148	162	3.66	>0.1
1579	DLAKLAFKRSSKYDL	HLA-DRB1*08:01	148	162	6.39	Not predicted
2281	DLAKLAFKRSSKYDL	HLA-DRB1*13:01	148	162	9.03	>0.1
592	LAKLAFKRSSKYDLE	HLA-DRB1*07:01	149	163	2.55	>0.1
898	LAKLAFKRSSKYDLE	HLA-DRB1*03:01	149	163	3.66	>0.1
1040	LAKLAFKRSSKYDLE	HLA-DRB1*11:01	149	163	4.24	>0.1
1580	LAKLAFKRSSKYDLE	HLA-DRB1*08:01	149	163	6.39	Not predicted
2282	LAKLAFKRSSKYDLE	HLA-DRB1*13:01	149	163	9.03	>0.1
593	AKLAFKRSSKYDLEC	HLA-DRB1*07:01	150	164	2.55	>0.1
899	AKLAFKRSSKYDLEC	HLA-DRB1*03:01	150	164	3.66	>0.1

1056	AKLAFKRSSKYDLEC	HLA-DRB1*11:01	150	164	4.33	>0.1
1581	AKLAFKRSSKYDLEC	HLA-DRB1*08:01	150	164	6.39	Not predicted
2283	AKLAFKRSSKYDLEC	HLA-DRB1*13:01	150	164	9.03	>0.1
594	KLAFKRSSKYDLECA	HLA-DRB1*07:01	151	165	2.55	0.0137
900	KLAFKRSSKYDLECA	HLA-DRB1*03:01	151	165	3.66	0.0588
1057	KLAFKRSSKYDLECA	HLA-DRB1*11:01	151	165	4.33	0.0253
1582	KLAFKRSSKYDLECA	HLA-DRB1*08:01	151	165	6.39	Not predicted
2284	KLAFKRSSKYDLECA	HLA-DRB1*13:01	151	165	9.03	>0.1
901	LAFKRSSKYDLECAQ	HLA-DRB1*03:01	152	166	3.66	>0.1
1058	LAFKRSSKYDLECAQ	HLA-DRB1*11:01	152	166	4.33	>0.1

1559	LAFKRSSKYDLECAQ	HLA-DRB1*07:01	152	166	6.33	>0.1
1583	LAFKRSSKYDLECAQ	HLA-DRB1*08:01	152	166	6.39	Not predicted
2285	LAFKRSSKYDLECAQ	HLA-DRB1*13:01	152	166	9.03	>0.1
1059	AFKRSSKYDLECAQI	HLA-DRB1*11:01	153	167	4.33	>0.1
1584	AFKRSSKYDLECAQI	HLA-DRB1*08:01	153	167	6.39	Not predicted
1635	AFKRSSKYDLECAQI	HLA-DRB1*03:01	153	167	6.51	>0.1
2286	AFKRSSKYDLECAQI	HLA-DRB1*13:01	153	167	9.03	>0.1
1060	FKRSSKYDLECAQIP	HLA-DRB1*11:01	154	168	4.33	>0.1
1558	FKRSSKYDLECAQIP	HLA-DRB1*03:01	154	168	6.33	>0.1
1585	FKRSSKYDLECAQIP	HLA-DRB1*08:01	154	168	6.39	Not predicted

2287	FKRSSKYDLECAQIP	HLA-DRB1*13:01	154	168	9.03	>0.1
1394	DLECAQIPVHMKSDA	HLA-DRB1*13:01	161	175	5.45	>0.1
1395	LECAQIPVHMKSDAS	HLA-DRB1*13:01	162	176	5.45	>0.1
2431	LECAQIPVHMKSDAS	HLA-DRB1*11:01	162	176	9.53	>0.1
1396	ECAQIPVHMKSDASK	HLA-DRB1*13:01	163	177	5.45	>0.1
1397	CAQIPVHMKSDASKF	HLA-DRB1*13:01	164	178	5.45	>0.1
1009	AQIPVHMKSDASKFT	HLA-DRB1*04:01	165	179	4.16	>0.1
1398	AQIPVHMKSDASKFT	HLA-DRB1*13:01	165	179	5.45	>0.1
1648	AQIPVHMKSDASKFT	HLA-DRB1*03:01	165	179	6.58	>0.1
1010	QIPVHMKSDASKFTH	HLA-DRB1*04:01	166	180	4.16	>0.1

1399	QIPVHMKSDASKFTH	HLA-DRB1*13:01	166	180	5.45	>0.1
1649	QIPVHMKSDASKFTH	HLA-DRB1*03:01	166	180	6.58	>0.1
1011	IPVHMKSDASKFTHE	HLA-DRB1*04:01	167	181	4.16	>0.1
1400	IPVHMKSDASKFTHE	HLA-DRB1*13:01	167	181	5.45	>0.1
1650	IPVHMKSDASKFTHE	HLA-DRB1*03:01	167	181	6.58	0.0118
1012	PVHMKSDASKFTHEK	HLA-DRB1*04:01	168	182	4.16	>0.1
1651	PVHMKSDASKFTHEK	HLA-DRB1*03:01	168	182	6.58	>0.1
1013	VHMKSDASKFTHEKP	HLA-DRB1*04:01	169	183	4.16	>0.1
1652	VHMKSDASKFTHEKP	HLA-DRB1*03:01	169	183	6.58	>0.1
1695	HMKSDASKFTHEKPE	HLA-DRB1*03:01	170	184	6.72	>0.1

2503	HMKSDASKFTHEKPE	HLA-DRB1*04:01	170	184	9.83	>0.1
2011	MKSDASKFTHEKPEG	HLA-DRB1*03:01	171	185	7.9	>0.1
1957	ASKFTHEKPEGYYNW	HLA-DRB1*03:01	175	189	7.76	>0.1
2511	HEKPEGYYNWHHGAV	HLA-DRB1*08:01	180	194	9.89	Not predicted
2388	EKPEGYYNWHHGAVQ	HLA-DRB1*08:01	181	195	9.35	Not predicted
2389	KPEGYYNWHHGAVQY	HLA-DRB1*08:01	182	196	9.35	Not predicted
1988	PEGYYNWHHGAVQYS	HLA-DRB1*08:01	183	197	7.82	Not predicted
2167	PEGYYNWHHGAVQYS	HLA-DRB1*11:01	183	197	8.49	0.0253
1989	EGYYNWHHGAVQYSG	HLA-DRB1*08:01	184	198	7.82	Not predicted
2168	EGYYNWHHGAVQYSG	HLA-DRB1*11:01	184	198	8.49	>0.1

1990	GYYNWHHGAVQYSGG	HLA-DRB1*08:01	185	199	7.82	Not predicted
2207	GYYNWHHGAVQYSGG	HLA-DRB1*11:01	185	199	8.64	>0.1
1991	YYNWHHGAVQYSGGR	HLA-DRB1*08:01	186	200	7.82	Not predicted
1992	YNWHHGAVQYSGGRF	HLA-DRB1*08:01	187	201	7.82	Not predicted
1806	NWHHGAVQYSGGRFT	HLA-DRB1*07:01	188	202	6.96	>0.1
1993	NWHHGAVQYSGGRFT	HLA-DRB1*08:01	188	202	7.82	Not predicted
2030	NWHHGAVQYSGGRFT	HLA-DRB1*13:01	188	202	7.95	>0.1
2169	NWHHGAVQYSGGRFT	HLA-DRB1*11:01	188	202	8.49	>0.1
1448	WHHGAVQYSGGRFTI	HLA-DRB1*07:01	189	203	5.63	>0.1
1994	WHHGAVQYSGGRFTI	HLA-DRB1*08:01	189	203	7.82	Not predicted

2031	WHHGAVQYSGGRFTI	HLA-DRB1*13:01	189	203	7.95	>0.1
2170	WHHGAVQYSGGRFTI	HLA-DRB1*11:01	189	203	8.49	>0.1
1629	HHGAVQYSGGRFTIP	HLA-DRB1*07:01	190	204	6.45	>0.1
2032	HHGAVQYSGGRFTIP	HLA-DRB1*13:01	190	204	7.95	>0.1
1821	HGAVQYSGGRFTIPT	HLA-DRB1*07:01	191	205	7.12	>0.1
2033	HGAVQYSGGRFTIPT	HLA-DRB1*13:01	191	205	7.95	>0.1
2034	GAVQYSGGRFTIPTG	HLA-DRB1*13:01	192	206	7.95	>0.1
2410	GAVQYSGGRFTIPTG	HLA-DRB1*07:01	192	206	9.43	>0.1
2035	AVQYSGGRFTIPTGA	HLA-DRB1*13:01	193	207	7.95	>0.1
2036	VQYSGGRFTIPTGAG	HLA-DRB1*13:01	194	208	7.95	>0.1

1234	DSGRPIFDNKGRVVA	HLA-DRB1*08:01	212	226	4.81	Not predicted
1235	SGRPIFDNKGRVVAI	HLA-DRB1*08:01	213	227	4.81	Not predicted
1236	GRPIFDNKGRVVAIV	HLA-DRB1*08:01	214	228	4.81	Not predicted
1237	RPIFDNKGRVVAIVL	HLA-DRB1*08:01	215	229	4.81	Not predicted
1238	PIFDNKGRVVAIVLG	HLA-DRB1*08:01	216	230	4.81	Not predicted
1239	IFDNKGRVVAIVLGG	HLA-DRB1*08:01	217	231	4.81	Not predicted
1240	FDNKGRVVAIVLGGA	HLA-DRB1*08:01	218	232	4.81	Not predicted
2092	FDNKGRVVAIVLGGA	HLA-DRB1*11:01	218	232	8.12	>0.1
1492	DNKGRVVAIVLGGAN	HLA-DRB1*08:01	219	233	5.97	Not predicted
1905	DNKGRVVAIVLGGAN	HLA-DRB1*11:01	219	233	7.45	>0.1

1493	NKGRVVAIVLGGANE	HLA-DRB1*08:01	220	234	5.97	Not predicted
1948	NKGRVVAIVLGGANE	HLA-DRB1*11:01	220	234	7.66	>0.1
1140	KGRVVAIVLGGANEG	HLA-DRB1*13:01	221	235	4.62	>0.1
1494	KGRVVAIVLGGANEG	HLA-DRB1*08:01	221	235	5.97	Not predicted
1906	KGRVVAIVLGGANEG	HLA-DRB1*11:01	221	235	7.45	0.0417
1141	GRVVAIVLGGANEGA	HLA-DRB1*13:01	222	236	4.62	>0.1
1495	GRVVAIVLGGANEGA	HLA-DRB1*08:01	222	236	5.97	Not predicted
2019	GRVVAIVLGGANEGA	HLA-DRB1*11:01	222	236	7.91	>0.1
1142	RVVAIVLGGANEGAR	HLA-DRB1*13:01	223	237	4.62	>0.1
1496	RVVAIVLGGANEGAR	HLA-DRB1*08:01	223	237	5.97	Not predicted

2227	RVVAIVLGGANEGAR	HLA-DRB1*11:01	223	237	8.82	>0.1
1143	VVAIVLGGANEGART	HLA-DRB1*13:01	224	238	4.62	>0.1
1497	VVAIVLGGANEGART	HLA-DRB1*08:01	224	238	5.97	Not predicted
1144	VAIVLGGANEGARTA	HLA-DRB1*13:01	225	239	4.62	>0.1
1145	AIVLGGANEGARTAL	HLA-DRB1*13:01	226	240	4.62	>0.1
1146	IVLGGANEGARTALS	HLA-DRB1*13:01	227	241	4.62	>0.1
530	ARTALSVVTWNKDIV	HLA-DRB1*13:01	236	250	2.37	>0.1
531	RTALSVVTWNKDIVT	HLA-DRB1*13:01	237	251	2.37	>0.1
1534	RTALSVVTWNKDIVT	HLA-DRB1*03:01	237	251	6.18	>0.1
532	TALSVVTWNKDIVTK	HLA-DRB1*13:01	238	252	2.37	>0.1

1535	TALSVVTWNKDIVTK	HLA-DRB1*03:01	238	252	6.18	>0.1
252	ALSVVTWNKDIVTKI	HLA-DRB1*03:01	239	253	1.14	>0.1
533	ALSVVTWNKDIVTKI	HLA-DRB1*13:01	239	253	2.37	>0.1
2495	ALSVVTWNKDIVTKI	HLA-DRB1*04:01	239	253	9.73	>0.1
253	LSVVTWNKDIVTKIT	HLA-DRB1*03:01	240	254	1.14	>0.1
534	LSVVTWNKDIVTKIT	HLA-DRB1*13:01	240	254	2.37	>0.1
2448	LSVVTWNKDIVTKIT	HLA-DRB1*04:01	240	254	9.6	>0.1
254	SVVTWNKDIVTKITP	HLA-DRB1*03:01	241	255	1.14	>0.1
535	SVVTWNKDIVTKITP	HLA-DRB1*13:01	241	255	2.37	>0.1
2500	SVVTWNKDIVTKITP	HLA-DRB1*04:01	241	255	9.81	>0.1

255	VVTWNKDIVTKITPE	HLA-DRB1*03:01	242	256	1.14	>0.1
536	VVTWNKDIVTKITPE	HLA-DRB1*13:01	242	256	2.37	>0.1
176	VTWNKDIVTKITPEG	HLA-DRB1*08:01	243	257	0.89	Not predicted
198	VTWNKDIVTKITPEG	HLA-DRB1*13:01	243	257	0.94	>0.1
256	VTWNKDIVTKITPEG	HLA-DRB1*03:01	243	257	1.14	>0.1
1908	VTWNKDIVTKITPEG	HLA-DRB1*11:01	243	257	7.46	>0.1
2010	VTWNKDIVTKITPEG	HLA-DRB1*04:01	243	257	7.89	>0.1
177	TWNKDIVTKITPEGA	HLA-DRB1*08:01	244	258	0.89	Not predicted
199	TWNKDIVTKITPEGA	HLA-DRB1*13:01	244	258	0.94	>0.1
519	TWNKDIVTKITPEGA	HLA-DRB1*03:01	244	258	2.34	>0.1

2099	TWNKDIVTKITPEGA	HLA-DRB1*11:01	244	258	8.22	>0.1
178	WNKDIVTKITPEGAE	HLA-DRB1*08:01	245	259	0.89	Not predicted
200	WNKDIVTKITPEGAE	HLA-DRB1*13:01	245	259	0.94	>0.1
520	WNKDIVTKITPEGAE	HLA-DRB1*03:01	245	259	2.35	>0.1
2151	WNKDIVTKITPEGAE	HLA-DRB1*11:01	245	259	8.42	>0.1
179	NKDIVTKITPEGAEE	HLA-DRB1*08:01	246	260	0.89	Not predicted
201	NKDIVTKITPEGAEE	HLA-DRB1*13:01	246	260	0.94	>0.1
180	KDIVTKITPEGAEEW	HLA-DRB1*08:01	247	261	0.89	Not predicted
202	KDIVTKITPEGAEEW	HLA-DRB1*13:01	247	261	0.94	>0.1
181	DIVTKITPEGAEEWS	HLA-DRB1*08:01	248	262	0.89	Not predicted

203	DIVTKITPEGAEEWS	HLA-DRB1*13:01	248	262	0.94	>0.1
182	IVTKITPEGAEEWSL	HLA-DRB1*08:01	249	263	0.89	Not predicted
204	IVTKITPEGAEEWSL	HLA-DRB1*13:01	249	263	0.94	>0.1
1841	EEWSLAIPVMCLLAN	HLA-DRB1*08:01	259	273	7.32	Not predicted
2369	EEWSLAIPVMCLLAN	HLA-DRB1*11:01	259	273	9.22	>0.1
1842	EWSLAIPVMCLLANT	HLA-DRB1*08:01	260	274	7.32	Not predicted
1466	WSLAIPVMCLLANTT	HLA-DRB1*11:01	261	275	5.82	>0.1
1471	WSLAIPVMCLLANTT	HLA-DRB1*04:01	261	275	5.83	>0.1
1843	WSLAIPVMCLLANTT	HLA-DRB1*08:01	261	275	7.32	Not predicted
2484	WSLAIPVMCLLANTT	HLA-DRB1*13:01	261	275	9.71	>0.1

978	SLAIPVMCLLANTTF	HLA-DRB1*04:01	262	276	3.94	>0.1
1817	SLAIPVMCLLANTTF	HLA-DRB1*11:01	262	276	7.1	>0.1
1844	SLAIPVMCLLANTTF	HLA-DRB1*08:01	262	276	7.32	Not predicted
1884	SLAIPVMCLLANTTF	HLA-DRB1*15:01	262	276	7.41	>0.1
2020	SLAIPVMCLLANTTF	HLA-DRB1*01:01	262	276	7.92	>0.1
2485	SLAIPVMCLLANTTF	HLA-DRB1*13:01	262	276	9.71	>0.1
835	LAIPVMCLLANTTFP	HLA-DRB1*04:01	263	277	3.35	>0.1
1818	LAIPVMCLLANTTFP	HLA-DRB1*11:01	263	277	7.1	>0.1
1845	LAIPVMCLLANTTFP	HLA-DRB1*08:01	263	277	7.32	Not predicted
1885	LAIPVMCLLANTTFP	HLA-DRB1*15:01	263	277	7.41	>0.1

2257	LAIPVMCLLANTTFP	HLA-DRB1*01:01	263	277	8.9	>0.1
2486	LAIPVMCLLANTTFP	HLA-DRB1*13:01	263	277	9.71	>0.1
666	AIPVMCLLANTTFPC	HLA-DRB1*04:01	264	278	2.76	>0.1
771	AIPVMCLLANTTFPC	HLA-DRB1*13:01	264	278	3.23	>0.1
1671	AIPVMCLLANTTFPC	HLA-DRB1*11:01	264	278	6.64	>0.1
1808	AIPVMCLLANTTFPC	HLA-DRB1*15:01	264	278	6.96	0.0577
1846	AIPVMCLLANTTFPC	HLA-DRB1*08:01	264	278	7.32	Not predicted
2007	AIPVMCLLANTTFPC	HLA-DRB1*01:01	264	278	7.86	>0.1
667	IPVMCLLANTTFPCS	HLA-DRB1*04:01	265	279	2.76	>0.1
772	IPVMCLLANTTFPCS	HLA-DRB1*13:01	265	279	3.23	>0.1

1672	IPVMCLLANTTFPCS	HLA-DRB1*11:01	265	279	6.64	0.0605
1809	IPVMCLLANTTFPCS	HLA-DRB1*15:01	265	279	6.96	>0.1
1847	IPVMCLLANTTFPCS	HLA-DRB1*08:01	265	279	7.32	Not predicted
2146	IPVMCLLANTTFPCS	HLA-DRB1*01:01	265	279	8.36	>0.1
668	PVMCLLANTTFPCSR	HLA-DRB1*04:01	266	280	2.76	>0.1
773	PVMCLLANTTFPCSR	HLA-DRB1*13:01	266	280	3.23	>0.1
1673	PVMCLLANTTFPCSR	HLA-DRB1*11:01	266	280	6.64	>0.1
2444	PVMCLLANTTFPCSR	HLA-DRB1*03:01	266	280	9.55	>0.1
774	VMCLLANTTFPCSRP	HLA-DRB1*13:01	267	281	3.23	>0.1
999	VMCLLANTTFPCSRP	HLA-DRB1*04:01	267	281	4.11	>0.1

2255	VMCLLANTTFPCSRP	HLA-DRB1*11:01	267	281	8.87	>0.1
775	MCLLANTTFPCSRPP	HLA-DRB1*13:01	268	282	3.23	>0.1
1562	MCLLANTTFPCSRPP	HLA-DRB1*04:01	268	282	6.35	>0.1
776	CLLANTTFPCSRPPC	HLA-DRB1*13:01	269	283	3.23	>0.1
777	LLANTTFPCSRPPCT	HLA-DRB1*13:01	270	284	3.23	>0.1
548	KEPEKTLRMLEDNVM	HLA-DRB1*13:01	290	304	2.4	>0.1
1195	KEPEKTLRMLEDNVM	HLA-DRB1*04:01	290	304	4.74	>0.1
2376	KEPEKTLRMLEDNVM	HLA-DRB1*11:01	290	304	9.34	>0.1
2446	KEPEKTLRMLEDNVM	HLA-DRB1*03:01	290	304	9.57	>0.1
404	EPEKTLRMLEDNVMS	HLA-DRB1*04:01	291	305	1.72	>0.1

549	EPEKTLRMLEDNVMS	HLA-DRB1*13:01	291	305	2.4	>0.1
2497	EPEKTLRMLEDNVMS	HLA-DRB1*03:01	291	305	9.77	>0.1
165	PEKTLRMLEDNVMSP	HLA-DRB1*03:01	292	306	0.87	>0.1
317	PEKTLRMLEDNVMSP	HLA-DRB1*04:01	292	306	1.35	>0.1
550	PEKTLRMLEDNVMSP	HLA-DRB1*13:01	292	306	2.4	>0.1
212	EKTLRMLEDNVMSPG	HLA-DRB1*03:01	293	307	0.95	>0.1
318	EKTLRMLEDNVMSPG	HLA-DRB1*04:01	293	307	1.35	>0.1
551	EKTLRMLEDNVMSPG	HLA-DRB1*13:01	293	307	2.4	>0.1
193	KTLRMLEDNVMSPGY	HLA-DRB1*03:01	294	308	0.93	>0.1
319	KTLRMLEDNVMSPGY	HLA-DRB1*04:01	294	308	1.35	>0.1

552	KTLRMLEDNVMSPGY	HLA-DRB1*13:01	294	308	2.4	>0.1
194	TLRMLEDNVMSPGYY	HLA-DRB1*03:01	295	309	0.93	0.0214
553	TLRMLEDNVMSPGYY	HLA-DRB1*13:01	295	309	2.4	>0.1
590	TLRMLEDNVMSPGYY	HLA-DRB1*04:01	295	309	2.54	>0.1
196	LRMLEDNVMSPGYYQ	HLA-DRB1*03:01	296	310	0.94	>0.1
554	LRMLEDNVMSPGYYQ	HLA-DRB1*13:01	296	310	2.4	>0.1
1554	LRMLEDNVMSPGYYQ	HLA-DRB1*04:01	296	310	6.29	>0.1
984	RMLEDNVMSPGYYQL	HLA-DRB1*03:01	297	311	3.97	>0.1
998	MLEDNVMSPGYYQLL	HLA-DRB1*03:01	298	312	4.08	>0.1
330	NVMSPGYYQLLQASL	HLA-DRB1*01:01	302	316	1.36	>0.1

1322	NVMSPGYYQLLQASL	HLA-DRB1*08:01	302	316	5.17	Not predicted
1381	NVMSPGYYQLLQASL	HLA-DRB1*11:01	302	316	5.43	>0.1
2192	NVMSPGYYQLLQASL	HLA-DRB1*04:01	302	316	8.52	>0.1
1	VMSPGYYQLLQASLT	HLA-DRB1*01:01	303	317	0.01	>0.1
737	VMSPGYYQLLQASLT	HLA-DRB1*04:01	303	317	3.03	>0.1
1305	VMSPGYYQLLQASLT	HLA-DRB1*11:01	303	317	5.07	>0.1
1314	VMSPGYYQLLQASLT	HLA-DRB1*15:01	303	317	5.12	>0.1
1323	VMSPGYYQLLQASLT	HLA-DRB1*08:01	303	317	5.17	Not predicted
1472	VMSPGYYQLLQASLT	HLA-DRB1*07:01	303	317	5.85	>0.1
2	MSPGYYQLLQASLTC	HLA-DRB1*01:01	304	318	0.01	>0.1

467	MSPGYYQLLQASLTC	HLA-DRB1*04:01	304	318	2.15	>0.1
1084	MSPGYYQLLQASLTC	HLA-DRB1*11:01	304	318	4.36	>0.1
1324	MSPGYYQLLQASLTC	HLA-DRB1*08:01	304	318	5.17	Not predicted
1668	MSPGYYQLLQASLTC	HLA-DRB1*07:01	304	318	6.62	>0.1
2197	MSPGYYQLLQASLTC	HLA-DRB1*15:01	304	318	8.57	>0.1
3	SPGYYQLLQASLTCS	HLA-DRB1*01:01	305	319	0.01	>0.1
378	SPGYYQLLQASLTCS	HLA-DRB1*04:01	305	319	1.57	>0.1
511	SPGYYQLLQASLTCS	HLA-DRB1*11:01	305	319	2.28	>0.1
1325	SPGYYQLLQASLTCS	HLA-DRB1*08:01	305	319	5.17	Not predicted
2037	SPGYYQLLQASLTCS	HLA-DRB1*13:01	305	319	7.95	>0.1

2107	SPGYYQLLQASLTCS	HLA-DRB1*07:01	305	319	8.3	>0.1
2198	SPGYYQLLQASLTCS	HLA-DRB1*15:01	305	319	8.57	>0.1
4	PGYYQLLQASLTCSP	HLA-DRB1*01:01	306	320	0.01	>0.1
457	PGYYQLLQASLTCSP	HLA-DRB1*04:01	306	320	2.05	>0.1
601	PGYYQLLQASLTCSP	HLA-DRB1*11:01	306	320	2.57	>0.1
1326	PGYYQLLQASLTCSP	HLA-DRB1*08:01	306	320	5.17	Not predicted
2038	PGYYQLLQASLTCSP	HLA-DRB1*13:01	306	320	7.95	>0.1
2102	PGYYQLLQASLTCSP	HLA-DRB1*03:01	306	320	8.25	>0.1
2199	PGYYQLLQASLTCSP	HLA-DRB1*15:01	306	320	8.57	0.0537
5	GYYQLLQASLTCSPR	HLA-DRB1*01:01	307	321	0.04	>0.1

802	GYYQLLQASLTCSPR	HLA-DRB1*04:01	307	321	3.26	>0.1
1090	GYYQLLQASLTCSPR	HLA-DRB1*11:01	307	321	4.37	0.00134
1327	GYYQLLQASLTCSPR	HLA-DRB1*08:01	307	321	5.17	Not predicted
2039	GYYQLLQASLTCSPR	HLA-DRB1*13:01	307	321	7.95	>0.1
2085	GYYQLLQASLTCSPR	HLA-DRB1*03:01	307	321	8.06	0.0365
2200	GYYQLLQASLTCSPR	HLA-DRB1*15:01	307	321	8.57	>0.1
49	YYQLLQASLTCSPRR	HLA-DRB1*01:01	308	322	0.28	0.0208
803	YYQLLQASLTCSPRR	HLA-DRB1*04:01	308	322	3.26	>0.1
1306	YYQLLQASLTCSPRR	HLA-DRB1*11:01	308	322	5.07	>0.1
1328	YYQLLQASLTCSPRR	HLA-DRB1*08:01	308	322	5.17	Not predicted

2040	YYQLLQASLTCSPRR	HLA-DRB1*13:01	308	322	7.95	>0.1
2213	YYQLLQASLTCSPRR	HLA-DRB1*03:01	308	322	8.68	>0.1
570	YQLLQASLTCSPRRQ	HLA-DRB1*01:01	309	323	2.46	>0.1
840	YQLLQASLTCSPRRQ	HLA-DRB1*04:01	309	323	3.36	>0.1
1307	YQLLQASLTCSPRRQ	HLA-DRB1*11:01	309	323	5.07	>0.1
1329	YQLLQASLTCSPRRQ	HLA-DRB1*08:01	309	323	5.17	Not predicted
2041	YQLLQASLTCSPRRQ	HLA-DRB1*13:01	309	323	7.95	>0.1
2208	YQLLQASLTCSPRRQ	HLA-DRB1*03:01	309	323	8.65	>0.1
1147	QLLQASLTCSPRRQR	HLA-DRB1*13:01	310	324	4.62	>0.1
1204	QLLQASLTCSPRRQR	HLA-DRB1*03:01	310	324	4.77	>0.1

1148	LLQASLTCSPRRQRR	HLA-DRB1*13:01	311	325	4.62	>0.1
1205	LLQASLTCSPRRQRR	HLA-DRB1*03:01	311	325	4.77	>0.1
1149	LQASLTCSPRRQRRS	HLA-DRB1*13:01	312	326	4.62	>0.1
1206	LQASLTCSPRRQRRS	HLA-DRB1*03:01	312	326	4.77	>0.1
1150	QASLTCSPRRQRRSI	HLA-DRB1*13:01	313	327	4.62	>0.1
1151	ASLTCSPRRQRRSIK	HLA-DRB1*13:01	314	328	4.62	>0.1
1152	SLTCSPRRQRRSIKD	HLA-DRB1*13:01	315	329	4.62	>0.1
1153	LTCSPRRQRRSIKDH	HLA-DRB1*13:01	316	330	4.62	>0.1
1833	RSIKDHFNVYKATRP	HLA-DRB1*04:01	325	339	7.22	>0.1
2022	RSIKDHFNVYKATRP	HLA-DRB1*07:01	325	339	7.93	>0.1

1632	SIKDHFNVYKATRPY	HLA-DRB1*07:01	326	340	6.47	>0.1
1949	SIKDHFNVYKATRPY	HLA-DRB1*04:01	326	340	7.67	>0.1
1055	IKDHFNVYKATRPYL	HLA-DRB1*07:01	327	341	4.33	>0.1
1076	IKDHFNVYKATRPYL	HLA-DRB1*03:01	327	341	4.35	>0.1
1656	IKDHFNVYKATRPYL	HLA-DRB1*15:01	327	341	6.58	>0.1
1820	IKDHFNVYKATRPYL	HLA-DRB1*04:01	327	341	7.11	>0.1
2171	IKDHFNVYKATRPYL	HLA-DRB1*13:01	327	341	8.49	>0.1
2432	IKDHFNVYKATRPYL	HLA-DRB1*11:01	327	341	9.53	>0.1
332	KDHFNVYKATRPYLA	HLA-DRB1*07:01	328	342	1.36	>0.1
912	KDHFNVYKATRPYLA	HLA-DRB1*04:01	328	342	3.71	>0.1

1077	KDHFNVYKATRPYLA	HLA-DRB1*03:01	328	342	4.35	>0.1
1082	KDHFNVYKATRPYLA	HLA-DRB1*01:01	328	342	4.36	0.0333
1467	KDHFNVYKATRPYLA	HLA-DRB1*11:01	328	342	5.82	>0.1
1555	KDHFNVYKATRPYLA	HLA-DRB1*15:01	328	342	6.3	>0.1
2172	KDHFNVYKATRPYLA	HLA-DRB1*13:01	328	342	8.49	>0.1
433	DHFNVYKATRPYLAH	HLA-DRB1*07:01	329	343	1.83	0.0101
843	DHFNVYKATRPYLAH	HLA-DRB1*11:01	329	343	3.37	>0.1
860	DHFNVYKATRPYLAH	HLA-DRB1*04:01	329	343	3.43	>0.1
1078	DHFNVYKATRPYLAH	HLA-DRB1*03:01	329	343	4.35	>0.1
1083	DHFNVYKATRPYLAH	HLA-DRB1*01:01	329	343	4.36	>0.1

1474	DHFNVYKATRPYLAH	HLA-DRB1*15:01	329	343	5.9	>0.1
2173	DHFNVYKATRPYLAH	HLA-DRB1*13:01	329	343	8.49	>0.1
503	HFNVYKATRPYLAHC	HLA-DRB1*07:01	330	344	2.22	>0.1
507	HFNVYKATRPYLAHC	HLA-DRB1*11:01	330	344	2.26	>0.1
566	HFNVYKATRPYLAHC	HLA-DRB1*04:01	330	344	2.44	>0.1
1079	HFNVYKATRPYLAHC	HLA-DRB1*03:01	330	344	4.35	0.017
1127	HFNVYKATRPYLAHC	HLA-DRB1*01:01	330	344	4.57	>0.1
1647	HFNVYKATRPYLAHC	HLA-DRB1*15:01	330	344	6.57	>0.1
2174	HFNVYKATRPYLAHC	HLA-DRB1*13:01	330	344	8.49	>0.1
438	FNVYKATRPYLAHCP	HLA-DRB1*11:01	331	345	1.88	0.0229

577	FNVYKATRPYLAHCP	HLA-DRB1*04:01	331	345	2.49	>0.1
834	FNVYKATRPYLAHCP	HLA-DRB1*07:01	331	345	3.33	>0.1
1080	FNVYKATRPYLAHCP	HLA-DRB1*03:01	331	345	4.35	>0.1
1273	FNVYKATRPYLAHCP	HLA-DRB1*01:01	331	345	4.99	>0.1
2175	FNVYKATRPYLAHCP	HLA-DRB1*13:01	331	345	8.49	>0.1
670	NVYKATRPYLAHCPD	HLA-DRB1*04:01	332	346	2.78	>0.1
709	NVYKATRPYLAHCPD	HLA-DRB1*11:01	332	346	2.9	>0.1
1081	NVYKATRPYLAHCPD	HLA-DRB1*03:01	332	346	4.35	>0.1
1350	NVYKATRPYLAHCPD	HLA-DRB1*01:01	332	346	5.2	>0.1
2023	NVYKATRPYLAHCPD	HLA-DRB1*07:01	332	346	7.93	>0.1

2176	NVYKATRPYLAHCPD	HLA-DRB1*13:01	332	346	8.49	>0.1
1276	VYKATRPYLAHCPDC	HLA-DRB1*11:01	333	347	5.01	>0.1
1545	VYKATRPYLAHCPDC	HLA-DRB1*04:01	333	347	6.24	>0.1
2177	VYKATRPYLAHCPDC	HLA-DRB1*13:01	333	347	8.49	>0.1
2221	VYKATRPYLAHCPDC	HLA-DRB1*03:01	333	347	8.78	>0.1
1468	YKATRPYLAHCPDCG	HLA-DRB1*11:01	334	348	5.82	>0.1
2277	YKATRPYLAHCPDCG	HLA-DRB1*04:01	334	348	8.98	>0.1
2012	HSCHSPVALERIRNE	HLA-DRB1*13:01	351	365	7.9	>0.1
2390	HSCHSPVALERIRNE	HLA-DRB1*08:01	351	365	9.35	Not predicted
2013	SCHSPVALERIRNEA	HLA-DRB1*13:01	352	366	7.9	>0.1

2391	SCHSPVALERIRNEA	HLA-DRB1*08:01	352	366	9.35	Not predicted
1317	CHSPVALERIRNEAT	HLA-DRB1*04:01	353	367	5.14	>0.1
2014	CHSPVALERIRNEAT	HLA-DRB1*13:01	353	367	7.9	>0.1
2392	CHSPVALERIRNEAT	HLA-DRB1*08:01	353	367	9.35	Not predicted
1318	HSPVALERIRNEATD	HLA-DRB1*04:01	354	368	5.14	>0.1
2015	HSPVALERIRNEATD	HLA-DRB1*13:01	354	368	7.9	>0.1
2393	HSPVALERIRNEATD	HLA-DRB1*08:01	354	368	9.35	Not predicted
1319	SPVALERIRNEATDG	HLA-DRB1*04:01	355	369	5.14	>0.1
2016	SPVALERIRNEATDG	HLA-DRB1*13:01	355	369	7.9	>0.1
2394	SPVALERIRNEATDG	HLA-DRB1*08:01	355	369	9.35	Not predicted

922	PVALERIRNEATDGT	HLA-DRB1*04:01	356	370	3.77	>0.1
2017	PVALERIRNEATDGT	HLA-DRB1*13:01	356	370	7.9	>0.1
2395	PVALERIRNEATDGT	HLA-DRB1*08:01	356	370	9.35	Not predicted
913	VALERIRNEATDGTL	HLA-DRB1*04:01	357	371	3.71	>0.1
2018	VALERIRNEATDGTL	HLA-DRB1*13:01	357	371	7.9	>0.1
2396	VALERIRNEATDGTL	HLA-DRB1*08:01	357	371	9.35	Not predicted
2278	ALERIRNEATDGTLK	HLA-DRB1*04:01	358	372	8.99	>0.1
2190	LERIRNEATDGTLKI	HLA-DRB1*04:01	359	373	8.5	>0.1
69	EATDGTLKIQVSLQI	HLA-DRB1*13:01	365	379	0.45	>0.1
393	EATDGTLKIQVSLQI	HLA-DRB1*15:01	365	379	1.67	>0.1

440	EATDGTLKIQVSLQI	HLA-DRB1*11:01	365	379	1.94	>0.1
694	EATDGTLKIQVSLQI	HLA-DRB1*04:01	365	379	2.83	>0.1
985	EATDGTLKIQVSLQI	HLA-DRB1*07:01	365	379	3.99	>0.1
1825	EATDGTLKIQVSLQI	HLA-DRB1*03:01	365	379	7.16	>0.1
70	ATDGTLKIQVSLQIG	HLA-DRB1*13:01	366	380	0.45	>0.1
413	ATDGTLKIQVSLQIG	HLA-DRB1*15:01	366	380	1.73	>0.1
454	ATDGTLKIQVSLQIG	HLA-DRB1*11:01	366	380	2.01	>0.1
669	ATDGTLKIQVSLQIG	HLA-DRB1*04:01	366	380	2.76	>0.1
1297	ATDGTLKIQVSLQIG	HLA-DRB1*07:01	366	380	5.07	>0.1
2073	ATDGTLKIQVSLQIG	HLA-DRB1*03:01	366	380	7.98	>0.1

71	TDGTLKIQVSLQIGI	HLA-DRB1*13:01	367	381	0.45	>0.1
381	TDGTLKIQVSLQIGI	HLA-DRB1*15:01	367	381	1.57	>0.1
392	TDGTLKIQVSLQIGI	HLA-DRB1*07:01	367	381	1.67	>0.1
395	TDGTLKIQVSLQIGI	HLA-DRB1*11:01	367	381	1.7	>0.1
449	TDGTLKIQVSLQIGI	HLA-DRB1*03:01	367	381	1.99	>0.1
629	TDGTLKIQVSLQIGI	HLA-DRB1*04:01	367	381	2.64	>0.1
72	DGTLKIQVSLQIGIK	HLA-DRB1*13:01	368	382	0.45	>0.1
364	DGTLKIQVSLQIGIK	HLA-DRB1*15:01	368	382	1.52	>0.1
383	DGTLKIQVSLQIGIK	HLA-DRB1*11:01	368	382	1.61	>0.1
450	DGTLKIQVSLQIGIK	HLA-DRB1*03:01	368	382	1.99	0.0615

455	DGTLKIQVSLQIGIK	HLA-DRB1*07:01	368	382	2.03	0.0817
598	DGTLKIQVSLQIGIK	HLA-DRB1*04:01	368	382	2.57	>0.1
73	GTLKIQVSLQIGIKT	HLA-DRB1*13:01	369	383	0.45	>0.1
341	GTLKIQVSLQIGIKT	HLA-DRB1*11:01	369	383	1.42	>0.1
346	GTLKIQVSLQIGIKT	HLA-DRB1*15:01	369	383	1.45	>0.1
451	GTLKIQVSLQIGIKT	HLA-DRB1*03:01	369	383	1.99	>0.1
572	GTLKIQVSLQIGIKT	HLA-DRB1*07:01	369	383	2.46	>0.1
731	GTLKIQVSLQIGIKT	HLA-DRB1*04:01	369	383	2.94	>0.1
74	TLKIQVSLQIGIKTD	HLA-DRB1*13:01	370	384	0.45	>0.1
452	TLKIQVSLQIGIKTD	HLA-DRB1*03:01	370	384	1.99	>0.1

761	TLKIQVSLQIGIKTD	HLA-DRB1*11:01	370	384	3.2	>0.1
800	TLKIQVSLQIGIKTD	HLA-DRB1*07:01	370	384	3.25	>0.1
1033	TLKIQVSLQIGIKTD	HLA-DRB1*15:01	370	384	4.17	>0.1
1823	TLKIQVSLQIGIKTD	HLA-DRB1*04:01	370	384	7.13	>0.1
75	LKIQVSLQIGIKTDD	HLA-DRB1*13:01	371	385	0.45	>0.1
302	LKIQVSLQIGIKTDD	HLA-DRB1*03:01	371	385	1.33	>0.1
798	LKIQVSLQIGIKTDD	HLA-DRB1*11:01	371	385	3.24	>0.1
1249	LKIQVSLQIGIKTDD	HLA-DRB1*07:01	371	385	4.85	>0.1
1320	LKIQVSLQIGIKTDD	HLA-DRB1*15:01	371	385	5.14	0.0864
2100	LKIQVSLQIGIKTDD	HLA-DRB1*04:01	371	385	8.23	>0.1

2115	LKIQVSLQIGIKTDD	HLA-DRB1*08:01	371	385	8.32	Not predicted
1258	KIQVSLQIGIKTDDS	HLA-DRB1*03:01	372	386	4.93	>0.1
1922	KIQVSLQIGIKTDDS	HLA-DRB1*11:01	372	386	7.59	>0.1
2116	KIQVSLQIGIKTDDS	HLA-DRB1*08:01	372	386	8.32	Not predicted
1131	IQVSLQIGIKTDDSH	HLA-DRB1*03:01	373	387	4.62	>0.1
1923	IQVSLQIGIKTDDSH	HLA-DRB1*11:01	373	387	7.59	>0.1
2117	IQVSLQIGIKTDDSH	HLA-DRB1*08:01	373	387	8.32	Not predicted
2118	QVSLQIGIKTDDSHD	HLA-DRB1*08:01	374	388	8.32	Not predicted
736	VSLQIGIKTDDSHDW	HLA-DRB1*03:01	375	389	3.03	>0.1
2119	VSLQIGIKTDDSHDW	HLA-DRB1*08:01	375	389	8.32	Not predicted

756	SLQIGIKTDDSHDWT	HLA-DRB1*03:01	376	390	3.12	>0.1
2120	SLQIGIKTDDSHDWT	HLA-DRB1*08:01	376	390	8.32	Not predicted
749	LQIGIKTDDSHDWTK	HLA-DRB1*03:01	377	391	3.07	>0.1
2121	LQIGIKTDDSHDWTK	HLA-DRB1*08:01	377	391	8.32	Not predicted
2449	QIGIKTDDSHDWTKL	HLA-DRB1*03:01	378	392	9.61	0.0281
2450	IGIKTDDSHDWTKLR	HLA-DRB1*03:01	379	393	9.61	>0.1
2451	GIKTDDSHDWTKLRY	HLA-DRB1*03:01	380	394	9.61	>0.1
2452	IKTDDSHDWTKLRYM	HLA-DRB1*03:01	381	395	9.61	>0.1
397	SHDWTKLRYMDNHMP	HLA-DRB1*13:01	386	400	1.71	>0.1
1291	SHDWTKLRYMDNHMP	HLA-DRB1*11:01	386	400	5.04	>0.1

1364	SHDWTKLRYMDNHMP	HLA-DRB1*04:01	386	400	5.24	>0.1
1697	SHDWTKLRYMDNHMP	HLA-DRB1*15:01	386	400	6.72	>0.1
1717	SHDWTKLRYMDNHMP	HLA-DRB1*08:01	386	400	6.87	Not predicted
398	HDWTKLRYMDNHMPA	HLA-DRB1*13:01	387	401	1.71	>0.1
1253	HDWTKLRYMDNHMPA	HLA-DRB1*04:01	387	401	4.88	>0.1
1292	HDWTKLRYMDNHMPA	HLA-DRB1*11:01	387	401	5.04	>0.1
1486	HDWTKLRYMDNHMPA	HLA-DRB1*15:01	387	401	5.95	>0.1
1718	HDWTKLRYMDNHMPA	HLA-DRB1*08:01	387	401	6.87	Not predicted
399	DWTKLRYMDNHMPAD	HLA-DRB1*13:01	388	402	1.71	>0.1
1268	DWTKLRYMDNHMPAD	HLA-DRB1*04:01	388	402	4.98	>0.1

1293	DWTKLRYMDNHMPAD	HLA-DRB1*11:01	388	402	5.04	>0.1
1712	DWTKLRYMDNHMPAD	HLA-DRB1*15:01	388	402	6.83	>0.1
1719	DWTKLRYMDNHMPAD	HLA-DRB1*08:01	388	402	6.87	Not predicted
400	WTKLRYMDNHMPADA	HLA-DRB1*13:01	389	403	1.71	>0.1
1217	WTKLRYMDNHMPADA	HLA-DRB1*04:01	389	403	4.81	>0.1
1294	WTKLRYMDNHMPADA	HLA-DRB1*11:01	389	403	5.04	>0.1
1720	WTKLRYMDNHMPADA	HLA-DRB1*08:01	389	403	6.87	Not predicted
2256	WTKLRYMDNHMPADA	HLA-DRB1*15:01	389	403	8.88	0.0278
401	TKLRYMDNHMPADAE	HLA-DRB1*13:01	390	404	1.71	>0.1
1262	TKLRYMDNHMPADAE	HLA-DRB1*04:01	390	404	4.96	>0.1

1295	TKLRYMDNHMPADAE	HLA-DRB1*11:01	390	404	5.04	>0.1
1721	TKLRYMDNHMPADAE	HLA-DRB1*08:01	390	404	6.87	Not predicted
402	KLRYMDNHMPADAER	HLA-DRB1*13:01	391	405	1.71	>0.1
1722	KLRYMDNHMPADAER	HLA-DRB1*08:01	391	405	6.87	Not predicted
2496	KLRYMDNHMPADAER	HLA-DRB1*11:01	391	405	9.74	0.0515
403	LRYMDNHMPADAERA	HLA-DRB1*13:01	392	406	1.71	>0.1
1723	LRYMDNHMPADAERA	HLA-DRB1*08:01	392	406	6.87	Not predicted
2322	RYMDNHMPADAERAG	HLA-DRB1*13:01	393	407	9.09	>0.1
2323	YMDNHMPADAERAGL	HLA-DRB1*13:01	394	408	9.09	>0.1
2324	MDNHMPADAERAGLF	HLA-DRB1*13:01	395	409	9.09	>0.1

476	DAERAGLFVRTSAPC	HLA-DRB1*08:01	402	416	2.17	Not predicted
2042	DAERAGLFVRTSAPC	HLA-DRB1*13:01	402	416	7.95	>0.1
390	AERAGLFVRTSAPCT	HLA-DRB1*07:01	403	417	1.64	>0.1
477	AERAGLFVRTSAPCT	HLA-DRB1*08:01	403	417	2.17	Not predicted
870	AERAGLFVRTSAPCT	HLA-DRB1*04:01	403	417	3.53	>0.1
1183	AERAGLFVRTSAPCT	HLA-DRB1*11:01	403	417	4.68	>0.1
1449	AERAGLFVRTSAPCT	HLA-DRB1*01:01	403	417	5.65	>0.1
2043	AERAGLFVRTSAPCT	HLA-DRB1*13:01	403	417	7.95	>0.1
2523	AERAGLFVRTSAPCT	HLA-DRB1*15:01	403	417	9.92	>0.1
261	ERAGLFVRTSAPCTI	HLA-DRB1*07:01	404	418	1.17	>0.1

478	ERAGLFVRTSAPCTI	HLA-DRB1*08:01	404	418	2.17	Not predicted
635	ERAGLFVRTSAPCTI	HLA-DRB1*13:01	404	418	2.65	>0.1
770	ERAGLFVRTSAPCTI	HLA-DRB1*03:01	404	418	3.23	>0.1
841	ERAGLFVRTSAPCTI	HLA-DRB1*04:01	404	418	3.37	>0.1
1184	ERAGLFVRTSAPCTI	HLA-DRB1*11:01	404	418	4.68	>0.1
1200	ERAGLFVRTSAPCTI	HLA-DRB1*01:01	404	418	4.77	>0.1
2371	ERAGLFVRTSAPCTI	HLA-DRB1*15:01	404	418	9.26	>0.1
362	RAGLFVRTSAPCTIT	HLA-DRB1*07:01	405	419	1.5	0.037
479	RAGLFVRTSAPCTIT	HLA-DRB1*08:01	405	419	2.17	Not predicted
636	RAGLFVRTSAPCTIT	HLA-DRB1*13:01	405	419	2.65	>0.1

760	RAGLFVRTSAPCTIT	HLA-DRB1*03:01	405	419	3.19	>0.1
836	RAGLFVRTSAPCTIT	HLA-DRB1*04:01	405	419	3.35	>0.1
1185	RAGLFVRTSAPCTIT	HLA-DRB1*11:01	405	419	4.68	>0.1
1201	RAGLFVRTSAPCTIT	HLA-DRB1*01:01	405	419	4.77	>0.1
2363	RAGLFVRTSAPCTIT	HLA-DRB1*15:01	405	419	9.19	0.0537
480	AGLFVRTSAPCTITG	HLA-DRB1*08:01	406	420	2.17	Not predicted
637	AGLFVRTSAPCTITG	HLA-DRB1*13:01	406	420	2.65	>0.1
660	AGLFVRTSAPCTITG	HLA-DRB1*07:01	406	420	2.72	>0.1
848	AGLFVRTSAPCTITG	HLA-DRB1*04:01	406	420	3.4	>0.1
850	AGLFVRTSAPCTITG	HLA-DRB1*03:01	406	420	3.42	0.0466

1123	AGLFVRTSAPCTITG	HLA-DRB1*11:01	406	420	4.53	>0.1
1202	AGLFVRTSAPCTITG	HLA-DRB1*01:01	406	420	4.77	>0.1
2411	AGLFVRTSAPCTITG	HLA-DRB1*15:01	406	420	9.43	>0.1
481	GLFVRTSAPCTITGT	HLA-DRB1*08:01	407	421	2.17	Not predicted
638	GLFVRTSAPCTITGT	HLA-DRB1*13:01	407	421	2.65	>0.1
801	GLFVRTSAPCTITGT	HLA-DRB1*07:01	407	421	3.25	>0.1
845	GLFVRTSAPCTITGT	HLA-DRB1*03:01	407	421	3.38	>0.1
917	GLFVRTSAPCTITGT	HLA-DRB1*04:01	407	421	3.73	>0.1
1186	GLFVRTSAPCTITGT	HLA-DRB1*11:01	407	421	4.68	0.0494
1274	GLFVRTSAPCTITGT	HLA-DRB1*01:01	407	421	4.99	0.0751

482	LFVRTSAPCTITGTM	HLA-DRB1*08:01	408	422	2.17	Not predicted
639	LFVRTSAPCTITGTM	HLA-DRB1*13:01	408	422	2.65	>0.1
846	LFVRTSAPCTITGTM	HLA-DRB1*07:01	408	422	3.39	>0.1
1038	LFVRTSAPCTITGTM	HLA-DRB1*03:01	408	422	4.23	>0.1
1187	LFVRTSAPCTITGTM	HLA-DRB1*11:01	408	422	4.68	>0.1
640	FVRTSAPCTITGTMG	HLA-DRB1*13:01	409	423	2.65	>0.1
1188	FVRTSAPCTITGTMG	HLA-DRB1*11:01	409	423	4.68	>0.1
1444	FVRTSAPCTITGTMG	HLA-DRB1*07:01	409	423	5.61	>0.1
2122	FVRTSAPCTITGTMG	HLA-DRB1*08:01	409	423	8.32	Not predicted
641	VRTSAPCTITGTMGH	HLA-DRB1*13:01	410	424	2.65	>0.1

2123	VRTSAPCTITGTMGH	HLA-DRB1*08:01	410	424	8.32	Not predicted
2101	TSAPCTITGTMGHFI	HLA-DRB1*07:01	412	426	8.24	>0.1
2098	SAPCTITGTMGHFIL	HLA-DRB1*07:01	413	427	8.22	>0.1
1600	CTITGTMGHFILARC	HLA-DRB1*13:01	416	430	6.41	>0.1
2397	CTITGTMGHFILARC	HLA-DRB1*08:01	416	430	9.35	Not predicted
1601	TITGTMGHFILARCP	HLA-DRB1*13:01	417	431	6.41	>0.1
2398	TITGTMGHFILARCP	HLA-DRB1*08:01	417	431	9.35	Not predicted
2479	TITGTMGHFILARCP	HLA-DRB1*11:01	417	431	9.69	>0.1
1602	ITGTMGHFILARCPK	HLA-DRB1*13:01	418	432	6.41	>0.1
1819	ITGTMGHFILARCPK	HLA-DRB1*11:01	418	432	7.1	>0.1

2364	ITGTMGHFILARCPK	HLA-DRB1*07:01	418	432	9.21	>0.1
2399	ITGTMGHFILARCPK	HLA-DRB1*08:01	418	432	9.35	Not predicted
1052	TGTMGHFILARCPKG	HLA-DRB1*11:01	419	433	4.3	>0.1
1422	TGTMGHFILARCPKG	HLA-DRB1*08:01	419	433	5.55	Not predicted
1452	TGTMGHFILARCPKG	HLA-DRB1*03:01	419	433	5.69	>0.1
1603	TGTMGHFILARCPKG	HLA-DRB1*13:01	419	433	6.41	>0.1
183	GTMGHFILARCPKGE	HLA-DRB1*08:01	420	434	0.89	Not predicted
1003	GTMGHFILARCPKGE	HLA-DRB1*11:01	420	434	4.13	>0.1
1453	GTMGHFILARCPKGE	HLA-DRB1*03:01	420	434	5.69	>0.1
1604	GTMGHFILARCPKGE	HLA-DRB1*13:01	420	434	6.41	>0.1

184	TMGHFILARCPKGET	HLA-DRB1*08:01	421	435	0.89	Not predicted
700	TMGHFILARCPKGET	HLA-DRB1*11:01	421	435	2.88	>0.1
1454	TMGHFILARCPKGET	HLA-DRB1*03:01	421	435	5.69	0.0562
1605	TMGHFILARCPKGET	HLA-DRB1*13:01	421	435	6.41	>0.1
185	MGHFILARCPKGETL	HLA-DRB1*08:01	422	436	0.89	Not predicted
515	MGHFILARCPKGETL	HLA-DRB1*11:01	422	436	2.31	>0.1
1455	MGHFILARCPKGETL	HLA-DRB1*03:01	422	436	5.69	>0.1
1606	MGHFILARCPKGETL	HLA-DRB1*13:01	422	436	6.41	>0.1
186	GHFILARCPKGETLT	HLA-DRB1*08:01	423	437	0.89	Not predicted
926	GHFILARCPKGETLT	HLA-DRB1*11:01	423	437	3.8	0.00936

1456	GHFILARCPKGETLT	HLA-DRB1*03:01	423	437	5.69	>0.1
2044	GHFILARCPKGETLT	HLA-DRB1*13:01	423	437	7.95	>0.1
187	HFILARCPKGETLTV	HLA-DRB1*08:01	424	438	0.89	Not predicted
1053	HFILARCPKGETLTV	HLA-DRB1*11:01	424	438	4.3	>0.1
2004	HFILARCPKGETLTV	HLA-DRB1*03:01	424	438	7.84	>0.1
2045	HFILARCPKGETLTV	HLA-DRB1*13:01	424	438	7.95	>0.1
188	FILARCPKGETLTVG	HLA-DRB1*08:01	425	439	0.89	Not predicted
1054	FILARCPKGETLTVG	HLA-DRB1*11:01	425	439	4.3	>0.1
2046	FILARCPKGETLTVG	HLA-DRB1*13:01	425	439	7.95	>0.1
2086	FILARCPKGETLTVG	HLA-DRB1*03:01	425	439	8.06	>0.1

189	ILARCPKGETLTVGF	HLA-DRB1*08:01	426	440	0.89	Not predicted
2047	ILARCPKGETLTVGF	HLA-DRB1*13:01	426	440	7.95	>0.1
1264	KGETLTVGFTDGRKI	HLA-DRB1*07:01	432	446	4.96	>0.1
2288	KGETLTVGFTDGRKI	HLA-DRB1*13:01	432	446	9.03	>0.1
1371	GETLTVGFTDGRKIS	HLA-DRB1*07:01	433	447	5.38	>0.1
2135	GETLTVGFTDGRKIS	HLA-DRB1*03:01	433	447	8.34	>0.1
2289	GETLTVGFTDGRKIS	HLA-DRB1*13:01	433	447	9.03	>0.1
1539	ETLTVGFTDGRKISH	HLA-DRB1*07:01	434	448	6.2	>0.1
2136	ETLTVGFTDGRKISH	HLA-DRB1*03:01	434	448	8.34	>0.1
2290	ETLTVGFTDGRKISH	HLA-DRB1*13:01	434	448	9.03	>0.1

1837	TLTVGFTDGRKISHS	HLA-DRB1*07:01	435	449	7.29	>0.1
2137	TLTVGFTDGRKISHS	HLA-DRB1*03:01	435	449	8.34	>0.1
2291	TLTVGFTDGRKISHS	HLA-DRB1*13:01	435	449	9.03	>0.1
2138	LTVGFTDGRKISHSC	HLA-DRB1*03:01	436	450	8.34	>0.1
2292	LTVGFTDGRKISHSC	HLA-DRB1*13:01	436	450	9.03	>0.1
2375	LTVGFTDGRKISHSC	HLA-DRB1*07:01	436	450	9.34	>0.1
2293	TVGFTDGRKISHSCT	HLA-DRB1*13:01	437	451	9.03	>0.1
2294	VGFTDGRKISHSCTH	HLA-DRB1*13:01	438	452	9.03	>0.1
316	FTDGRKISHSCTHPF	HLA-DRB1*07:01	440	454	1.34	>0.1
2178	FTDGRKISHSCTHPF	HLA-DRB1*13:01	440	454	8.49	>0.1

2453	FTDGRKISHSCTHPF	HLA-DRB1*03:01	440	454	9.61	>0.1
405	TDGRKISHSCTHPFH	HLA-DRB1*07:01	441	455	1.72	>0.1
2179	TDGRKISHSCTHPFH	HLA-DRB1*13:01	441	455	8.49	>0.1
2454	TDGRKISHSCTHPFH	HLA-DRB1*03:01	441	455	9.61	>0.1
504	DGRKISHSCTHPFHH	HLA-DRB1*07:01	442	456	2.22	>0.1
2180	DGRKISHSCTHPFHH	HLA-DRB1*13:01	442	456	8.49	>0.1
2455	DGRKISHSCTHPFHH	HLA-DRB1*03:01	442	456	9.61	>0.1
847	GRKISHSCTHPFHHD	HLA-DRB1*07:01	443	457	3.39	>0.1
2181	GRKISHSCTHPFHHD	HLA-DRB1*13:01	443	457	8.49	>0.1
2456	GRKISHSCTHPFHHD	HLA-DRB1*03:01	443	457	9.61	>0.1

921	RKISHSCTHPFHHDP	HLA-DRB1*07:01	444	458	3.75	0.0615
2182	RKISHSCTHPFHHDP	HLA-DRB1*13:01	444	458	8.49	>0.1
2457	RKISHSCTHPFHHDP	HLA-DRB1*03:01	444	458	9.61	>0.1
1457	KISHSCTHPFHHDPP	HLA-DRB1*07:01	445	459	5.7	>0.1
2183	KISHSCTHPFHHDPP	HLA-DRB1*13:01	445	459	8.49	>0.1
1960	ISHSCTHPFHHDPPV	HLA-DRB1*07:01	446	460	7.8	>0.1
2184	ISHSCTHPFHHDPPV	HLA-DRB1*13:01	446	460	8.49	>0.1
596	HSCTHPFHHDPPVIG	HLA-DRB1*03:01	448	462	2.57	>0.1
1816	HSCTHPFHHDPPVIG	HLA-DRB1*04:01	448	462	7.1	>0.1
583	SCTHPFHHDPPVIGR	HLA-DRB1*03:01	449	463	2.51	>0.1

1763	SCTHPFHHDPPVIGR	HLA-DRB1*04:01	449	463	6.9	>0.1
579	CTHPFHHDPPVIGRE	HLA-DRB1*03:01	450	464	2.5	>0.1
1713	CTHPFHHDPPVIGRE	HLA-DRB1*04:01	450	464	6.84	>0.1
**565**	**THPFHHDPPVIGREK**	**HLA-DRB1*03:01**	**451**	**465**	2.43	**>0.1**
1670	**THPFHHDPPVIGREK**	**HLA-DRB1*04:01**	**451**	**465**	6.63	**>0.1**
569	**HPFHHDPPVIGREKF**	**HLA-DRB1*03:01**	**452**	**466**	2.45	**>0.1**
1805	**HPFHHDPPVIGREKF**	**HLA-DRB1*04:01**	**452**	**466**	6.96	**>0.1**
2209	**PFHHDPPVIGREKFH**	**HLA-DRB1*03:01**	**453**	**467**	8.65	**>0.1**
25	**FHHDPPVIGREKFHS**	**HLA-DRB1*13:01**	**454**	**468**	0.23	**>0.1**
95	FHHDPPVIGREKFHS	HLA-DRB1*08:01	454	468	0.55	Not predicted

1640	**FHHDPPVIGREKFHS**	**HLA-DRB1*11:01**	**454**	**468**	6.54	**>0.1**
2149	**FHHDPPVIGREKFHS**	**HLA-DRB1*03:01**	**454**	**468**	8.38	**>0.1**
26	**HHDPPVIGREKFHSR**	**HLA-DRB1*13:01**	**455**	**469**	0.23	**>0.1**
96	HHDPPVIGREKFHSR	HLA-DRB1*08:01	455	469	0.55	Not predicted
27	**HDPPVIGREKFHSRP**	**HLA-DRB1*13:01**	**456**	**470**	0.23	**>0.1**
97	HDPPVIGREKFHSRP	HLA-DRB1*08:01	456	470	0.55	Not predicted
28	**DPPVIGREKFHSRPQ**	**HLA-DRB1*13:01**	**457**	**471**	0.23	**>0.1**
98	DPPVIGREKFHSRPQ	HLA-DRB1*08:01	457	471	0.55	Not predicted
1461	**DPPVIGREKFHSRPQ**	**HLA-DRB1*11:01**	**457**	**471**	5.76	**>0.1**
29	PPVIGREKFHSRPQH	HLA-DRB1*13:01	458	472	0.23	>0.1

99	PPVIGREKFHSRPQH	HLA-DRB1*08:01	458	472	0.55	Not predicted
1352	PPVIGREKFHSRPQH	HLA-DRB1*11:01	458	472	5.22	>0.1
30	PVIGREKFHSRPQHG	HLA-DRB1*13:01	459	473	0.23	>0.1
100	PVIGREKFHSRPQHG	HLA-DRB1*08:01	459	473	0.55	Not predicted
1491	PVIGREKFHSRPQHG	HLA-DRB1*11:01	459	473	5.97	>0.1
31	VIGREKFHSRPQHGR	HLA-DRB1*13:01	460	474	0.23	>0.1
101	VIGREKFHSRPQHGR	HLA-DRB1*08:01	460	474	0.55	Not predicted
928	VIGREKFHSRPQHGR	HLA-DRB1*11:01	460	474	3.83	>0.1
1498	IGREKFHSRPQHGRE	HLA-DRB1*08:01	461	475	5.97	Not predicted
1499	GREKFHSRPQHGREL	HLA-DRB1*08:01	462	476	5.97	Not predicted

1500	REKFHSRPQHGRELP	HLA-DRB1*08:01	463	477	5.97	Not predicted
1501	EKFHSRPQHGRELPC	HLA-DRB1*08:01	464	478	5.97	Not predicted
1502	KFHSRPQHGRELPCS	HLA-DRB1*08:01	465	479	5.97	Not predicted
1503	FHSRPQHGRELPCST	HLA-DRB1*08:01	466	480	5.97	Not predicted
571	ELPCSTYAQSTAATA	HLA-DRB1*04:01	475	489	2.46	>0.1
350	LPCSTYAQSTAATAE	HLA-DRB1*04:01	476	490	1.47	>0.1
195	PCSTYAQSTAATAEE	HLA-DRB1*04:01	477	491	0.93	>0.1
161	**CSTYAQSTAATAEEI**	**HLA-DRB1*04:01**	**478**	**492**	0.83	**>0.1**
1540	CSTYAQSTAATAEEI	HLA-DRB1*01:01	478	492	6.21	0.0511
297	**STYAQSTAATAEEIE**	**HLA-DRB1*04:01**	**479**	**493**	1.25	**>0.1**

1945	**STYAQSTAATAEEIE**	**HLA-DRB1*01:01**	**479**	**493**	7.66	**>0.1**
662	**TYAQSTAATAEEIEV**	**HLA-DRB1*04:01**	**480**	**494**	2.73	**>0.1**
1417	**YAQSTAATAEEIEVH**	**HLA-DRB1*04:01**	**481**	**495**	5.48	**>0.1**
1768	AATAEEIEVHMPPDT	HLA-DRB1*13:01	486	500	6.91	>0.1
1769	ATAEEIEVHMPPDTP	HLA-DRB1*13:01	487	501	6.91	>0.1
1770	TAEEIEVHMPPDTPD	HLA-DRB1*13:01	488	502	6.91	>0.1
1771	AEEIEVHMPPDTPDR	HLA-DRB1*13:01	489	503	6.91	>0.1
1772	EEIEVHMPPDTPDRT	HLA-DRB1*13:01	490	504	6.91	>0.1
1773	EIEVHMPPDTPDRTL	HLA-DRB1*13:01	491	505	6.91	>0.1
1774	IEVHMPPDTPDRTLM	HLA-DRB1*13:01	492	506	6.91	>0.1

2157	DTPDRTLMSQQSGNV	HLA-DRB1*04:01	499	513	8.45	>0.1
2474	DTPDRTLMSQQSGNV	HLA-DRB1*03:01	499	513	9.65	>0.1
2158	TPDRTLMSQQSGNVK	HLA-DRB1*04:01	500	514	8.45	>0.1
1556	PDRTLMSQQSGNVKI	HLA-DRB1*07:01	501	515	6.31	>0.1
2159	PDRTLMSQQSGNVKI	HLA-DRB1*04:01	501	515	8.45	>0.1
1636	DRTLMSQQSGNVKIT	HLA-DRB1*07:01	502	516	6.51	>0.1
2160	DRTLMSQQSGNVKIT	HLA-DRB1*04:01	502	516	8.45	>0.1
1458	RTLMSQQSGNVKITV	HLA-DRB1*07:01	503	517	5.7	>0.1
2161	RTLMSQQSGNVKITV	HLA-DRB1*04:01	503	517	8.45	>0.1
1824	TLMSQQSGNVKITVN	HLA-DRB1*07:01	504	518	7.14	>0.1

2525	TLMSQQSGNVKITVN	HLA-DRB1*04:01	504	518	9.93	>0.1
2414	LMSQQSGNVKITVNS	HLA-DRB1*07:01	505	519	9.46	>0.1
1915	QQSGNVKITVNSQTV	HLA-DRB1*07:01	508	522	7.52	>0.1
2139	QSGNVKITVNSQTVR	HLA-DRB1*03:01	509	523	8.34	>0.1
2443	QSGNVKITVNSQTVR	HLA-DRB1*07:01	509	523	9.54	>0.1
895	SGNVKITVNSQTVRY	HLA-DRB1*03:01	510	524	3.62	0.0444
2478	SGNVKITVNSQTVRY	HLA-DRB1*04:01	510	524	9.69	>0.1
127	GNVKITVNSQTVRYK	HLA-DRB1*13:01	511	525	0.71	>0.1
423	GNVKITVNSQTVRYK	HLA-DRB1*03:01	511	525	1.78	>0.1
2077	GNVKITVNSQTVRYK	HLA-DRB1*11:01	511	525	8.05	>0.1

128	NVKITVNSQTVRYKC	HLA-DRB1*13:01	512	526	0.71	>0.1
214	NVKITVNSQTVRYKC	HLA-DRB1*03:01	512	526	0.98	>0.1
2078	NVKITVNSQTVRYKC	HLA-DRB1*11:01	512	526	8.05	>0.1
129	VKITVNSQTVRYKCN	HLA-DRB1*13:01	513	527	0.71	>0.1
436	VKITVNSQTVRYKCN	HLA-DRB1*03:01	513	527	1.87	>0.1
2196	VKITVNSQTVRYKCN	HLA-DRB1*11:01	513	527	8.56	>0.1
130	KITVNSQTVRYKCNC	HLA-DRB1*13:01	514	528	0.71	>0.1
868	KITVNSQTVRYKCNC	HLA-DRB1*03:01	514	528	3.5	>0.1
131	ITVNSQTVRYKCNCG	HLA-DRB1*13:01	515	529	0.71	>0.1
1907	ITVNSQTVRYKCNCG	HLA-DRB1*03:01	515	529	7.46	>0.1

18	TVNSQTVRYKC NCGD	HLA- DRB1*08:0 1	516	530	0.18	Not predicte d
13 2	TVNSQTVRYKC NCGD	HLA- DRB1*13:0 1	516	530	0.71	>0.1
19	VNSQTVRYKCN CGDS	HLA- DRB1*08:0 1	517	531	0.18	Not predicte d
13 3	VNSQTVRYKCN CGDS	HLA- DRB1*13:0 1	517	531	0.71	>0.1
20	NSQTVRYKCNC GDSS	HLA- DRB1*08:0 1	518	532	0.18	Not predicte d
76 4	NSQTVRYKCNC GDSS	HLA- DRB1*13:0 1	518	532	3.21	>0.1
21	SQTVRYKCNCG DSSE	HLA- DRB1*08:0 1	519	533	0.18	Not predicte d
76 5	SQTVRYKCNCG DSSE	HLA- DRB1*13:0 1	519	533	3.21	>0.1
22	QTVRYKCNCG DSSEG	HLA- DRB1*08:0 1	520	534	0.18	Not predicte d
76 6	QTVRYKCNCG DSSEG	HLA- DRB1*13:0 1	520	534	3.21	>0.1

23	TVRYKCNCGDSSEGL	HLA-DRB1*08:01	521	535	0.18	Not predicted
767	TVRYKCNCGDSSEGL	HLA-DRB1*13:01	521	535	3.21	>0.1
24	VRYKCNCGDSSEGLT	HLA-DRB1*08:01	522	536	0.18	Not predicted
768	VRYKCNCGDSSEGLT	HLA-DRB1*13:01	522	536	3.21	>0.1
687	LTTTDKVINNCKVDQ	HLA-DRB1*08:01	535	549	2.82	Not predicted
696	LTTTDKVINNCKVDQ	HLA-DRB1*03:01	535	549	2.87	>0.1
712	LTTTDKVINNCKVDQ	HLA-DRB1*13:01	535	549	2.91	>0.1
1904	LTTTDKVINNCKVDQ	HLA-DRB1*11:01	535	549	7.44	>0.1
688	TTTDKVINNCKVDQC	HLA-DRB1*08:01	536	550	2.82	Not predicted
710	TTTDKVINNCKVDQC	HLA-DRB1*03:01	536	550	2.91	>0.1

713	TTTDKVINNCKVDQC	HLA-DRB1*13:01	536	550	2.91	>0.1
2134	TTTDKVINNCKVDQC	HLA-DRB1*11:01	536	550	8.33	>0.1
689	TTDKVINNCKVDQCH	HLA-DRB1*08:01	537	551	2.82	Not predicted
714	TTDKVINNCKVDQCH	HLA-DRB1*13:01	537	551	2.91	>0.1
721	TTDKVINNCKVDQCH	HLA-DRB1*03:01	537	551	2.93	>0.1
2258	TTDKVINNCKVDQCH	HLA-DRB1*11:01	537	551	8.9	>0.1
690	TDKVINNCKVDQCHA	HLA-DRB1*08:01	538	552	2.82	Not predicted
715	TDKVINNCKVDQCHA	HLA-DRB1*13:01	538	552	2.91	>0.1
719	TDKVINNCKVDQCHA	HLA-DRB1*03:01	538	552	2.92	>0.1
691	DKVINNCKVDQCHAA	HLA-DRB1*08:01	539	553	2.82	Not predicted

699	DKVINNCKVDQCHAA	HLA-DRB1*03:01	539	553	2.88	>0.1
716	DKVINNCKVDQCHAA	HLA-DRB1*13:01	539	553	2.91	>0.1
692	KVINNCKVDQCHAAV	HLA-DRB1*08:01	540	554	2.82	Not predicted
717	KVINNCKVDQCHAAV	HLA-DRB1*13:01	540	554	2.91	>0.1
2211	KVINNCKVDQCHAAV	HLA-DRB1*03:01	540	554	8.67	0.0901
693	VINNCKVDQCHAAVT	HLA-DRB1*08:01	541	555	2.82	Not predicted
718	VINNCKVDQCHAAVT	HLA-DRB1*13:01	541	555	2.91	>0.1
2105	VINNCKVDQCHAAVT	HLA-DRB1*03:01	541	555	8.3	>0.1
110	DQCHAAVTNHKKWQY	HLA-DRB1*13:01	548	562	0.6	>0.1
2458	DQCHAAVTNHKKWQY	HLA-DRB1*03:01	548	562	9.61	>0.1

111	QCHAAVTNHKKWQYN	HLA-DRB1*13:01	549	563	0.6	>0.1
2459	QCHAAVTNHKKWQYN	HLA-DRB1*03:01	549	563	9.61	>0.1
112	CHAAVTNHKKWQYNS	HLA-DRB1*13:01	550	564	0.6	>0.1
2460	CHAAVTNHKKWQYNS	HLA-DRB1*03:01	550	564	9.61	>0.1
113	HAAVTNHKKWQYNSP	HLA-DRB1*13:01	551	565	0.6	>0.1
2461	HAAVTNHKKWQYNSP	HLA-DRB1*03:01	551	565	9.61	>0.1
114	AAVTNHKKWQYNSPL	HLA-DRB1*13:01	552	566	0.6	>0.1
2462	AAVTNHKKWQYNSPL	HLA-DRB1*03:01	552	566	9.61	>0.1
115	AVTNHKKWQYNSPLV	HLA-DRB1*13:01	553	567	0.6	>0.1
2215	AVTNHKKWQYNSPLV	HLA-DRB1*15:01	553	567	8.71	>0.1

116	VTNHKKWQYNSPLVP	HLA-DRB1*13:01	554	568	0.6	>0.1
1330	VTNHKKWQYNSPLVP	HLA-DRB1*08:01	554	568	5.17	Not predicted
1639	VTNHKKWQYNSPLVP	HLA-DRB1*11:01	554	568	6.53	>0.1
2259	VTNHKKWQYNSPLVP	HLA-DRB1*15:01	554	568	8.91	>0.1
2420	VTNHKKWQYNSPLVP	HLA-DRB1*03:01	554	568	9.47	>0.1
1331	TNHKKWQYNSPLVPR	HLA-DRB1*08:01	555	569	5.17	Not predicted
1607	TNHKKWQYNSPLVPR	HLA-DRB1*13:01	555	569	6.41	>0.1
1660	TNHKKWQYNSPLVPR	HLA-DRB1*11:01	555	569	6.59	>0.1
2303	TNHKKWQYNSPLVPR	HLA-DRB1*15:01	555	569	9.04	>0.1
2421	TNHKKWQYNSPLVPR	HLA-DRB1*03:01	555	569	9.47	>0.1

1332	NHKKWQYNSPLVPRN	HLA-DRB1*08:01	556	570	5.17	Not predicted
1522	NHKKWQYNSPLVPRN	HLA-DRB1*11:01	556	570	6.05	>0.1
1608	NHKKWQYNSPLVPRN	HLA-DRB1*13:01	556	570	6.41	>0.1
2222	NHKKWQYNSPLVPRN	HLA-DRB1*04:01	556	570	8.78	>0.1
2358	NHKKWQYNSPLVPRN	HLA-DRB1*15:01	556	570	9.15	>0.1
2422	NHKKWQYNSPLVPRN	HLA-DRB1*03:01	556	570	9.47	>0.1
1333	HKKWQYNSPLVPRNA	HLA-DRB1*08:01	557	571	5.17	Not predicted
1451	HKKWQYNSPLVPRNA	HLA-DRB1*11:01	557	571	5.68	>0.1
1609	HKKWQYNSPLVPRNA	HLA-DRB1*13:01	557	571	6.41	>0.1
2423	HKKWQYNSPLVPRNA	HLA-DRB1*03:01	557	571	9.47	>0.1

2498	HKKWQYNSPLVPRNA	HLA-DRB1*04:01	557	571	9.78	>0.1
1334	KKWQYNSPLVPRNAE	HLA-DRB1*08:01	558	572	5.17	Not predicted
1416	KKWQYNSPLVPRNAE	HLA-DRB1*11:01	558	572	5.47	>0.1
1610	KKWQYNSPLVPRNAE	HLA-DRB1*13:01	558	572	6.41	>0.1
2424	KKWQYNSPLVPRNAE	HLA-DRB1*03:01	558	572	9.47	>0.1
1335	KWQYNSPLVPRNAEF	HLA-DRB1*08:01	559	573	5.17	Not predicted
1611	KWQYNSPLVPRNAEF	HLA-DRB1*13:01	559	573	6.41	>0.1
1014	WQYNSPLVPRNAEFG	HLA-DRB1*08:01	560	574	4.16	Not predicted
1283	WQYNSPLVPRNAEFG	HLA-DRB1*13:01	560	574	5.02	>0.1
1015	QYNSPLVPRNAEFGD	HLA-DRB1*08:01	561	575	4.16	Not predicted

1284	QYNSPLVPRNAEFGD	HLA-DRB1*13:01	561	575	5.02	>0.1
1016	YNSPLVPRNAEFGDR	HLA-DRB1*08:01	562	576	4.16	Not predicted
1285	YNSPLVPRNAEFGDR	HLA-DRB1*13:01	562	576	5.02	>0.1
1017	NSPLVPRNAEFGDRK	HLA-DRB1*08:01	563	577	4.16	Not predicted
1286	NSPLVPRNAEFGDRK	HLA-DRB1*13:01	563	577	5.02	>0.1
1018	SPLVPRNAEFGDRKG	HLA-DRB1*08:01	564	578	4.16	Not predicted
1287	SPLVPRNAEFGDRKG	HLA-DRB1*13:01	564	578	5.02	>0.1
1019	PLVPRNAEFGDRKGK	HLA-DRB1*08:01	565	579	4.16	Not predicted
1288	PLVPRNAEFGDRKGK	HLA-DRB1*13:01	565	579	5.02	>0.1
1020	LVPRNAEFGDRKGKV	HLA-DRB1*08:01	566	580	4.16	Not predicted

1289	LVPRNAEFGDRKGKV	HLA-DRB1*13:01	566	580	5.02	>0.1
2271	LVPRNAEFGDRKGKV	HLA-DRB1*03:01	566	580	8.98	>0.1
1423	VPRNAEFGDRKGKVH	HLA-DRB1*08:01	567	581	5.55	Not predicted
1424	PRNAEFGDRKGKVHI	HLA-DRB1*08:01	568	582	5.55	Not predicted
1425	RNAEFGDRKGKVHIP	HLA-DRB1*08:01	569	583	5.55	Not predicted
1426	NAEFGDRKGKVHIPF	HLA-DRB1*08:01	570	584	5.55	Not predicted
1427	AEFGDRKGKVHIPFP	HLA-DRB1*08:01	571	585	5.55	Not predicted
1428	EFGDRKGKVHIPFPL	HLA-DRB1*08:01	572	586	5.55	Not predicted
1429	FGDRKGKVHIPFPLA	HLA-DRB1*08:01	573	587	5.55	Not predicted
1504	GDRKGKVHIPFPLAN	HLA-DRB1*08:01	574	588	5.97	Not predicted

1505	DRKGKVHIPFPLANV	HLA-DRB1*08:01	575	589	5.97	Not predicted
1506	RKGKVHIPFPLANVT	HLA-DRB1*08:01	576	590	5.97	Not predicted
1507	KGKVHIPFPLANVTC	HLA-DRB1*08:01	577	591	5.97	Not predicted
1508	GKVHIPFPLANVTCR	HLA-DRB1*08:01	578	592	5.97	Not predicted
1509	KVHIPFPLANVTCRV	HLA-DRB1*08:01	579	593	5.97	Not predicted
1510	VHIPFPLANVTCRVP	HLA-DRB1*08:01	580	594	5.97	Not predicted
2325	PFPLANVTCRVPKAR	HLA-DRB1*13:01	583	597	9.09	>0.1
2326	FPLANVTCRVPKARN	HLA-DRB1*13:01	584	598	9.09	>0.1
2327	PLANVTCRVPKARNP	HLA-DRB1*13:01	585	599	9.09	>0.1
2328	LANVTCRVPKARNPT	HLA-DRB1*13:01	586	600	9.09	>0.1

2329	ANVTCRVPKARNPTV	HLA-DRB1*13:01	587	601	9.09	>0.1
2330	NVTCRVPKARNPTVT	HLA-DRB1*13:01	588	602	9.09	>0.1
2331	VTCRVPKARNPTVTY	HLA-DRB1*13:01	589	603	9.09	>0.1
1807	KARNPTVTYGKNQVI	HLA-DRB1*07:01	595	609	6.96	>0.1
1704	ARNPTVTYGKNQVIM	HLA-DRB1*07:01	596	610	6.77	>0.1
986	RNPTVTYGKNQVIML	HLA-DRB1*11:01	597	611	3.99	>0.1
1531	RNPTVTYGKNQVIML	HLA-DRB1*07:01	597	611	6.16	>0.1
1848	RNPTVTYGKNQVIML	HLA-DRB1*08:01	597	611	7.32	Not predicted
919	NPTVTYGKNQVIMLL	HLA-DRB1*11:01	598	612	3.73	>0.1
1519	NPTVTYGKNQVIMLL	HLA-DRB1*07:01	598	612	6.01	>0.1

1849	NPTVTYGKNQVIMLL	HLA-DRB1*08:01	598	612	7.32	Not predicted
983	PTVTYGKNQVIMLLY	HLA-DRB1*11:01	599	613	3.96	>0.1
1296	PTVTYGKNQVIMLLY	HLA-DRB1*07:01	599	613	5.05	>0.1
1850	PTVTYGKNQVIMLLY	HLA-DRB1*08:01	599	613	7.32	Not predicted
1312	TVTYGKNQVIMLLYP	HLA-DRB1*11:01	600	614	5.12	>0.1
1700	TVTYGKNQVIMLLYP	HLA-DRB1*07:01	600	614	6.73	>0.1
1851	TVTYGKNQVIMLLYP	HLA-DRB1*08:01	600	614	7.32	Not predicted
1321	VTYGKNQVIMLLYPD	HLA-DRB1*11:01	601	615	5.16	>0.1
1852	VTYGKNQVIMLLYPD	HLA-DRB1*08:01	601	615	7.32	Not predicted
1724	TYGKNQVIMLLYPDH	HLA-DRB1*08:01	602	616	6.87	Not predicted

2306	TYGKNQVIMLLYPDH	HLA-DRB1*11:01	602	616	9.05	>0.1
977	YGKNQVIMLLYPDHP	HLA-DRB1*11:01	603	617	3.93	>0.1
1725	YGKNQVIMLLYPDHP	HLA-DRB1*08:01	603	617	6.87	Not predicted
2224	YGKNQVIMLLYPDHP	HLA-DRB1*03:01	603	617	8.8	>0.1
2233	YGKNQVIMLLYPDHP	HLA-DRB1*15:01	603	617	8.83	>0.1
2332	YGKNQVIMLLYPDHP	HLA-DRB1*13:01	603	617	9.09	>0.1
1006	GKNQVIMLLYPDHPT	HLA-DRB1*11:01	604	618	4.14	>0.1
1267	GKNQVIMLLYPDHPT	HLA-DRB1*15:01	604	618	4.96	>0.1
1726	GKNQVIMLLYPDHPT	HLA-DRB1*08:01	604	618	6.87	Not predicted
2333	GKNQVIMLLYPDHPT	HLA-DRB1*13:01	604	618	9.09	>0.1

2475	GKNQVIMLLYPDHPT	HLA-DRB1*04:01	604	618	9.65	>0.1
924	KNQVIMLLYPDHPTL	HLA-DRB1*11:01	605	619	3.78	>0.1
1118	KNQVIMLLYPDHPTL	HLA-DRB1*15:01	605	619	4.48	0.00835
1727	KNQVIMLLYPDHPTL	HLA-DRB1*08:01	605	619	6.87	Not predicted
1913	KNQVIMLLYPDHPTL	HLA-DRB1*04:01	605	619	7.51	>0.1
2334	KNQVIMLLYPDHPTL	HLA-DRB1*13:01	605	619	9.09	>0.1
239	NQVIMLLYPDHPTLL	HLA-DRB1*03:01	606	620	1.12	0.017
916	NQVIMLLYPDHPTLL	HLA-DRB1*11:01	606	620	3.72	0.007
927	NQVIMLLYPDHPTLL	HLA-DRB1*15:01	606	620	3.81	>0.1
1628	NQVIMLLYPDHPTLL	HLA-DRB1*04:01	606	620	6.45	>0.1

1728	NQVIMLLYPDHPTLL	HLA-DRB1*08:01	606	620	6.87	Not predicted
2335	NQVIMLLYPDHPTLL	HLA-DRB1*13:01	606	620	9.09	>0.1
2361	NQVIMLLYPDHPTLL	HLA-DRB1*07:01	606	620	9.19	0.0313
240	QVIMLLYPDHPTLLS	HLA-DRB1*03:01	607	621	1.12	>0.1
930	QVIMLLYPDHPTLLS	HLA-DRB1*15:01	607	621	3.86	>0.1
981	QVIMLLYPDHPTLLS	HLA-DRB1*04:01	607	621	3.96	>0.1
1367	QVIMLLYPDHPTLLS	HLA-DRB1*11:01	607	621	5.29	>0.1
1729	QVIMLLYPDHPTLLS	HLA-DRB1*08:01	607	621	6.87	Not predicted
2336	QVIMLLYPDHPTLLS	HLA-DRB1*13:01	607	621	9.09	>0.1
241	VIMLLYPDHPTLLSY	HLA-DRB1*03:01	608	622	1.12	>0.1

1121	VIMLLYPDHPTLLSY	HLA-DRB1*15:01	608	622	4.5	>0.1
1261	VIMLLYPDHPTLLSY	HLA-DRB1*04:01	608	622	4.95	>0.1
1730	VIMLLYPDHPTLLSY	HLA-DRB1*08:01	608	622	6.87	Not predicted
2337	VIMLLYPDHPTLLSY	HLA-DRB1*13:01	608	622	9.09	>0.1
2372	VIMLLYPDHPTLLSY	HLA-DRB1*11:01	608	622	9.29	>0.1
146	IMLLYPDHPTLLSYR	HLA-DRB1*03:01	609	623	0.82	>0.1
1528	IMLLYPDHPTLLSYR	HLA-DRB1*04:01	609	623	6.11	>0.1
2096	IMLLYPDHPTLLSYR	HLA-DRB1*15:01	609	623	8.18	>0.1
2338	IMLLYPDHPTLLSYR	HLA-DRB1*13:01	609	623	9.09	>0.1
242	MLLYPDHPTLLSYRN	HLA-DRB1*03:01	610	624	1.12	>0.1

1630	MLLYPDHPTLLSYRN	HLA-DRB1*04:01	610	624	6.47	>0.1
243	LLYPDHPTLLSYRNM	HLA-DRB1*03:01	611	625	1.12	>0.1
2426	LLYPDHPTLLSYRNM	HLA-DRB1*04:01	611	625	9.5	>0.1
244	LYPDHPTLLSYRNMG	HLA-DRB1*03:01	612	626	1.12	>0.1
1469	LYPDHPTLLSYRNMG	HLA-DRB1*11:01	612	626	5.82	>0.1
351	YPDHPTLLSYRNMGE	HLA-DRB1*08:01	613	627	1.48	Not predicted
1218	YPDHPTLLSYRNMGE	HLA-DRB1*04:01	613	627	4.81	>0.1
1470	YPDHPTLLSYRNMGE	HLA-DRB1*11:01	613	627	5.82	>0.1
352	PDHPTLLSYRNMGEE	HLA-DRB1*08:01	614	628	1.48	Not predicted
353	DHPTLLSYRNMGEEP	HLA-DRB1*08:01	615	629	1.48	Not predicted

354	HPTLLSYRNMGEEPN	HLA-DRB1*08:01	616	630	1.48	Not predicted
2264	HPTLLSYRNMGEEPN	HLA-DRB1*04:01	616	630	8.96	>0.1
355	PTLLSYRNMGEEPNY	HLA-DRB1*08:01	617	631	1.48	Not predicted
2265	PTLLSYRNMGEEPNY	HLA-DRB1*04:01	617	631	8.96	>0.1
356	TLLSYRNMGEEPNYQ	HLA-DRB1*08:01	618	632	1.48	Not predicted
2266	**TLLSYRNMGEEPNYQ**	**HLA-DRB1*04:01**	**618**	**632**	**8.96**	**>0.1**
357	LLSYRNMGEEPNYQE	HLA-DRB1*08:01	619	633	1.48	Not predicted
2267	**LLSYRNMGEEPNYQE**	**HLA-DRB1*04:01**	**619**	**633**	**8.96**	**>0.1**
1021	LSYRNMGEEPNYQEE	HLA-DRB1*08:01	620	634	4.16	Not predicted
2268	**LSYRNMGEEPNYQEE**	**HLA-DRB1*04:01**	**620**	**634**	**8.96**	**>0.1**

1853	SYRNMGEEPNYQEEW	HLA-DRB1*08:01	621	635	7.32	Not predicted
1854	YRNMGEEPNYQEEWV	HLA-DRB1*08:01	622	636	7.32	Not predicted
**270**	**PNYQEEWVTHKKEIR**	**HLA-DRB1*13:01**	**629**	**643**	**1.2**	**>0.1**
**902**	**PNYQEEWVTHKKEIR**	**HLA-DRB1*03:01**	**629**	**643**	**3.67**	**>0.1**
**1661**	**PNYQEEWVTHKKEIR**	**HLA-DRB1*11:01**	**629**	**643**	**6.6**	**>0.1**
271	NYQEEWVTHKKEIRL	HLA-DRB1*13:01	630	644	1.2	>0.1
903	NYQEEWVTHKKEIRL	HLA-DRB1*03:01	630	644	3.67	>0.1
1662	NYQEEWVTHKKEIRL	HLA-DRB1*11:01	630	644	6.6	>0.1
272	YQEEWVTHKKEIRLT	HLA-DRB1*13:01	631	645	1.2	>0.1
904	YQEEWVTHKKEIRLT	HLA-DRB1*03:01	631	645	3.67	>0.1

1663	YQEEWVTHKKEIRLT	HLA-DRB1*11:01	631	645	6.6	>0.1
273	QEEWVTHKKEIRLTV	HLA-DRB1*13:01	632	646	1.2	>0.1
905	QEEWVTHKKEIRLTV	HLA-DRB1*03:01	632	646	3.67	>0.1
1664	QEEWVTHKKEIRLTV	HLA-DRB1*11:01	632	646	6.6	>0.1
274	EEWVTHKKEIRLTVP	HLA-DRB1*13:01	633	647	1.2	>0.1
906	EEWVTHKKEIRLTVP	HLA-DRB1*03:01	633	647	3.67	>0.1
1665	EEWVTHKKEIRLTVP	HLA-DRB1*11:01	633	647	6.6	>0.1
275	EWVTHKKEIRLTVPT	HLA-DRB1*13:01	634	648	1.2	>0.1
907	EWVTHKKEIRLTVPT	HLA-DRB1*03:01	634	648	3.67	>0.1
1666	EWVTHKKEIRLTVPT	HLA-DRB1*11:01	634	648	6.6	>0.1

276	WVTHKKEIRLTVPTE	HLA-DRB1*13:01	635	649	1.2	>0.1
908	WVTHKKEIRLTVPTE	HLA-DRB1*03:01	635	649	3.67	0.0797
1667	WVTHKKEIRLTVPTE	HLA-DRB1*11:01	635	649	6.6	>0.1
1154	VTHKKEIRLTVPTEG	HLA-DRB1*13:01	636	650	4.62	>0.1
1215	VTHKKEIRLTVPTEG	HLA-DRB1*03:01	636	650	4.79	>0.1
2433	VTHKKEIRLTVPTEG	HLA-DRB1*11:01	636	650	9.53	>0.1
1155	THKKEIRLTVPTEGL	HLA-DRB1*13:01	637	651	4.62	>0.1
1255	THKKEIRLTVPTEGL	HLA-DRB1*03:01	637	651	4.9	>0.1
2434	THKKEIRLTVPTEGL	HLA-DRB1*11:01	637	651	9.53	>0.1
1156	HKKEIRLTVPTEGLE	HLA-DRB1*13:01	638	652	4.62	>0.1

1182	HKKEIRLTVPTEGLE	HLA-DRB1*03:01	638	652	4.68	>0.1
2435	HKKEIRLTVPTEGLE	HLA-DRB1*11:01	638	652	9.53	>0.1
1095	KKEIRLTVPTEGLEV	HLA-DRB1*03:01	639	653	4.47	>0.1
1157	KKEIRLTVPTEGLEV	HLA-DRB1*13:01	639	653	4.62	>0.1
2436	KKEIRLTVPTEGLEV	HLA-DRB1*11:01	639	653	9.53	0.079
1128	KEIRLTVPTEGLEVT	HLA-DRB1*03:01	640	654	4.57	>0.1
1158	KEIRLTVPTEGLEVT	HLA-DRB1*13:01	640	654	4.62	>0.1
1159	EIRLTVPTEGLEVTW	HLA-DRB1*13:01	641	655	4.62	>0.1
1160	IRLTVPTEGLEVTWG	HLA-DRB1*13:01	642	656	4.62	>0.1
2378	EGLEVTWGNNEPYKY	HLA-DRB1*08:01	649	663	9.34	Not predicted

2379	GLEVTWGNNEPYKYW	HLA-DRB1*08:01	650	664	9.34	Not predicted
2380	LEVTWGNNEPYKYWP	HLA-DRB1*08:01	651	665	9.34	Not predicted
2381	EVTWGNNEPYKYWPQ	HLA-DRB1*08:01	652	666	9.34	Not predicted
2382	VTWGNNEPYKYWPQL	HLA-DRB1*08:01	653	667	9.34	Not predicted
2383	TWGNNEPYKYWPQLS	HLA-DRB1*08:01	654	668	9.34	Not predicted
490	WGNNEPYKYWPQLST	HLA-DRB1*11:01	655	669	2.21	>0.1
1586	WGNNEPYKYWPQLST	HLA-DRB1*08:01	655	669	6.39	Not predicted
2339	WGNNEPYKYWPQLST	HLA-DRB1*13:01	655	669	9.09	>0.1
491	GNNEPYKYWPQLSTN	HLA-DRB1*11:01	656	670	2.21	>0.1
1587	GNNEPYKYWPQLSTN	HLA-DRB1*08:01	656	670	6.39	Not predicted

2340	GNNEPYKYWPQLSTN	HLA-DRB1*13:01	656	670	9.09	>0.1
492	NNEPYKYWPQLSTNG	HLA-DRB1*11:01	657	671	2.21	>0.1
1588	NNEPYKYWPQLSTNG	HLA-DRB1*08:01	657	671	6.39	Not predicted
1838	NNEPYKYWPQLSTNG	HLA-DRB1*04:01	657	671	7.3	>0.1
2341	NNEPYKYWPQLSTNG	HLA-DRB1*13:01	657	671	9.09	>0.1
493	NEPYKYWPQLSTNGT	HLA-DRB1*11:01	658	672	2.21	>0.1
1259	NEPYKYWPQLSTNGT	HLA-DRB1*04:01	658	672	4.93	>0.1
1589	NEPYKYWPQLSTNGT	HLA-DRB1*08:01	658	672	6.39	Not predicted
2342	NEPYKYWPQLSTNGT	HLA-DRB1*13:01	658	672	9.09	>0.1
494	EPYKYWPQLSTNGTA	HLA-DRB1*11:01	659	673	2.21	0.0881

1198	EPYKYWPQLSTNGTA	HLA-DRB1*04:01	659	673	4.76	>0.1
1590	EPYKYWPQLSTNGTA	HLA-DRB1*08:01	659	673	6.39	Not predicted
2343	EPYKYWPQLSTNGTA	HLA-DRB1*13:01	659	673	9.09	>0.1
762	PYKYWPQLSTNGTAH	HLA-DRB1*11:01	660	674	3.2	>0.1
1196	PYKYWPQLSTNGTAH	HLA-DRB1*04:01	660	674	4.74	>0.1
1591	PYKYWPQLSTNGTAH	HLA-DRB1*08:01	660	674	6.39	Not predicted
2344	PYKYWPQLSTNGTAH	HLA-DRB1*13:01	660	674	9.09	>0.1
733	YKYWPQLSTNGTAHG	HLA-DRB1*11:01	661	675	2.97	>0.1
982	YKYWPQLSTNGTAHG	HLA-DRB1*04:01	661	675	3.96	>0.1
1592	YKYWPQLSTNGTAHG	HLA-DRB1*08:01	661	675	6.39	Not predicted

2345	YKYWPQLSTNGTAHG	HLA-DRB1*13:01	661	675	9.09	>0.1
1487	KYWPQLSTNGTAHGH	HLA-DRB1*04:01	662	676	5.96	>0.1
2094	YWPQLSTNGTAHGHP	HLA-DRB1*04:01	663	677	8.15	>0.1
1197	HGHPHEIILYYYELY	HLA-DRB1*15:01	674	688	4.75	>0.1
505	GHPHEIILYYYELYP	HLA-DRB1*15:01	675	689	2.25	>0.1
2400	GHPHEIILYYYELYP	HLA-DRB1*08:01	675	689	9.35	Not predicted
344	HPHEIILYYYELYPT	HLA-DRB1*15:01	676	690	1.43	>0.1
932	HPHEIILYYYELYPT	HLA-DRB1*08:01	676	690	3.86	Not predicted
1775	HPHEIILYYYELYPT	HLA-DRB1*13:01	676	690	6.91	>0.1
333	PHEIILYYYELYPTM	HLA-DRB1*15:01	677	691	1.37	>0.1

933	PHEIILYYYELYPTM	HLA-DRB1*08:01	677	691	3.86	Not predicted
1546	PHEIILYYYELYPTM	HLA-DRB1*04:01	677	691	6.24	>0.1
1776	PHEIILYYYELYPTM	HLA-DRB1*13:01	677	691	6.91	>0.1
334	HEIILYYYELYPTMT	HLA-DRB1*15:01	678	692	1.37	>0.1
624	HEIILYYYELYPTMT	HLA-DRB1*04:01	678	692	2.61	>0.1
934	HEIILYYYELYPTMT	HLA-DRB1*08:01	678	692	3.86	Not predicted
1008	HEIILYYYELYPTMT	HLA-DRB1*01:01	678	692	4.16	>0.1
1484	HEIILYYYELYPTMT	HLA-DRB1*07:01	678	692	5.91	>0.1
1777	HEIILYYYELYPTMT	HLA-DRB1*13:01	678	692	6.91	>0.1
77	EIILYYYELYPTMTA	HLA-DRB1*08:01	679	693	0.49	Not predicted

233	EIILYYYELYPTMTA	HLA-DRB1*11:01	679	693	1.08	>0.1
291	EIILYYYELYPTMTA	HLA-DRB1*04:01	679	693	1.21	>0.1
416	EIILYYYELYPTMTA	HLA-DRB1*15:01	679	693	1.75	0.000326
864	EIILYYYELYPTMTA	HLA-DRB1*01:01	679	693	3.49	>0.1
1563	EIILYYYELYPTMTA	HLA-DRB1*07:01	679	693	6.35	>0.1
1778	EIILYYYELYPTMTA	HLA-DRB1*13:01	679	693	6.91	>0.1
78	IILYYYELYPTMTAV	HLA-DRB1*08:01	680	694	0.49	Not predicted
234	IILYYYELYPTMTAV	HLA-DRB1*11:01	680	694	1.08	>0.1
260	IILYYYELYPTMTAV	HLA-DRB1*04:01	680	694	1.17	>0.1
627	IILYYYELYPTMTAV	HLA-DRB1*15:01	680	694	2.62	>0.1

795	IILYYYELYPTMTAV	HLA-DRB1*01:01	680	694	3.24	>0.1
1646	IILYYYELYPTMTAV	HLA-DRB1*07:01	680	694	6.56	>0.1
1779	IILYYYELYPTMTAV	HLA-DRB1*13:01	680	694	6.91	>0.1
79	ILYYYELYPTMTAVV	HLA-DRB1*08:01	681	695	0.49	Not predicted
235	ILYYYELYPTMTAVV	HLA-DRB1*11:01	681	695	1.08	0.0179
259	ILYYYELYPTMTAVV	HLA-DRB1*04:01	681	695	1.16	>0.1
663	ILYYYELYPTMTAVV	HLA-DRB1*01:01	681	695	2.74	>0.1
842	ILYYYELYPTMTAVV	HLA-DRB1*07:01	681	695	3.37	>0.1
1637	ILYYYELYPTMTAVV	HLA-DRB1*15:01	681	695	6.52	>0.1
1780	ILYYYELYPTMTAVV	HLA-DRB1*13:01	681	695	6.91	>0.1

80	LYYYELYPTMTAVVL	HLA-DRB1*08:01	682	696	0.49	Not predicted
236	LYYYELYPTMTAVVL	HLA-DRB1*11:01	682	696	1.08	>0.1
258	LYYYELYPTMTAVVL	HLA-DRB1*01:01	682	696	1.15	0.0252
300	LYYYELYPTMTAVVL	HLA-DRB1*04:01	682	696	1.3	>0.1
1004	LYYYELYPTMTAVVL	HLA-DRB1*07:01	682	696	4.14	>0.1
1781	LYYYELYPTMTAVVL	HLA-DRB1*13:01	682	696	6.91	>0.1
1882	LYYYELYPTMTAVVL	HLA-DRB1*15:01	682	696	7.34	>0.1
81	YYYELYPTMTAVVLS	HLA-DRB1*08:01	683	697	0.49	Not predicted
342	YYYELYPTMTAVVLS	HLA-DRB1*11:01	683	697	1.42	>0.1
379	YYYELYPTMTAVVLS	HLA-DRB1*04:01	683	697	1.57	>0.1

582	YYYELYPTMTAVVLS	HLA-DRB1*01:01	683	697	2.51	>0.1
1488	YYYELYPTMTAVVLS	HLA-DRB1*07:01	683	697	5.97	0.004
2185	YYYELYPTMTAVVLS	HLA-DRB1*13:01	683	697	8.49	>0.1
82	YYELYPTMTAVVLSV	HLA-DRB1*08:01	684	698	0.49	Not predicted
573	YYELYPTMTAVVLSV	HLA-DRB1*11:01	684	698	2.46	>0.1
739	YYELYPTMTAVVLSV	HLA-DRB1*04:01	684	698	3.05	>0.1
1035	YYELYPTMTAVVLSV	HLA-DRB1*01:01	684	698	4.22	>0.1
2075	YYELYPTMTAVVLSV	HLA-DRB1*07:01	684	698	8.01	>0.1
2186	YYELYPTMTAVVLSV	HLA-DRB1*13:01	684	698	8.49	>0.1
83	YELYPTMTAVVLSVA	HLA-DRB1*08:01	685	699	0.49	Not predicted

748	YELYPTMTAVVLSVA	HLA-DRB1*04:01	685	699	3.06	>0.1
992	YELYPTMTAVVLSVA	HLA-DRB1*11:01	685	699	4.03	>0.1
2187	YELYPTMTAVVLSVA	HLA-DRB1*13:01	685	699	8.49	>0.1
2360	YELYPTMTAVVLSVA	HLA-DRB1*01:01	685	699	9.19	>0.1
1119	ELYPTMTAVVLSVAS	HLA-DRB1*04:01	686	700	4.5	>0.1
2346	ELYPTMTAVVLSVAS	HLA-DRB1*13:01	686	700	9.09	>0.1
2469	ELYPTMTAVVLSVAS	HLA-DRB1*01:01	686	700	9.62	>0.1
1828	LYPTMTAVVLSVASF	HLA-DRB1*07:01	687	701	7.19	>0.1
1944	LYPTMTAVVLSVASF	HLA-DRB1*04:01	687	701	7.65	>0.1
2347	LYPTMTAVVLSVASF	HLA-DRB1*13:01	687	701	9.09	>0.1

84	YPTMTAVVLSVASFI	HLA-DRB1*03:01	688	702	0.51	>0.1
1041	YPTMTAVVLSVASFI	HLA-DRB1*13:01	688	702	4.25	>0.1
1536	YPTMTAVVLSVASFI	HLA-DRB1*04:01	688	702	6.18	>0.1
1537	YPTMTAVVLSVASFI	HLA-DRB1*07:01	688	702	6.18	>0.1
1941	YPTMTAVVLSVASFI	HLA-DRB1*15:01	688	702	7.63	>0.1
2437	YPTMTAVVLSVASFI	HLA-DRB1*11:01	688	702	9.53	>0.1
2470	YPTMTAVVLSVASFI	HLA-DRB1*01:01	688	702	9.62	>0.1
14	PTMTAVVLSVASFIL	HLA-DRB1*07:01	689	703	0.1	>0.1
85	PTMTAVVLSVASFIL	HLA-DRB1*03:01	689	703	0.51	>0.1
336	PTMTAVVLSVASFIL	HLA-DRB1*15:01	689	703	1.4	>0.1

1042	PTMTAVVLSVASFIL	HLA-DRB1*13:01	689	703	4.25	>0.1
1074	PTMTAVVLSVASFIL	HLA-DRB1*04:01	689	703	4.34	>0.1
2438	PTMTAVVLSVASFIL	HLA-DRB1*11:01	689	703	9.53	>0.1
9	TMTAVVLSVASFILL	HLA-DRB1*07:01	690	704	0.09	>0.1
86	TMTAVVLSVASFILL	HLA-DRB1*03:01	690	704	0.51	>0.1
337	TMTAVVLSVASFILL	HLA-DRB1*15:01	690	704	1.4	>0.1
1034	TMTAVVLSVASFILL	HLA-DRB1*04:01	690	704	4.2	>0.1
1043	TMTAVVLSVASFILL	HLA-DRB1*13:01	690	704	4.25	>0.1
2439	TMTAVVLSVASFILL	HLA-DRB1*11:01	690	704	9.53	>0.1
10	MTAVVLSVASFILLS	HLA-DRB1*07:01	691	705	0.09	>0.1

87	MTAVVLSVASFILLS	HLA-DRB1*03:01	691	705	0.51	>0.1
338	MTAVVLSVASFILLS	HLA-DRB1*15:01	691	705	1.4	>0.1
1044	MTAVVLSVASFILLS	HLA-DRB1*13:01	691	705	4.25	>0.1
1122	MTAVVLSVASFILLS	HLA-DRB1*04:01	691	705	4.51	>0.1
2440	MTAVVLSVASFILLS	HLA-DRB1*11:01	691	705	9.53	>0.1
11	TAVVLSVASFILLSM	HLA-DRB1*07:01	692	706	0.09	>0.1
88	TAVVLSVASFILLSM	HLA-DRB1*03:01	692	706	0.51	>0.1
118	TAVVLSVASFILLSM	HLA-DRB1*15:01	692	706	0.64	>0.1
642	TAVVLSVASFILLSM	HLA-DRB1*13:01	692	706	2.65	>0.1
759	TAVVLSVASFILLSM	HLA-DRB1*04:01	692	706	3.15	>0.1

1189	TAVVLSVASFILLSM	HLA-DRB1*11:01	692	706	4.7	>0.1
15	AVVLSVASFILLSMV	HLA-DRB1*07:01	693	707	0.14	>0.1
90	AVVLSVASFILLSMV	HLA-DRB1*03:01	693	707	0.53	>0.1
139	AVVLSVASFILLSMV	HLA-DRB1*15:01	693	707	0.76	>0.1
643	AVVLSVASFILLSMV	HLA-DRB1*13:01	693	707	2.65	>0.1
909	AVVLSVASFILLSMV	HLA-DRB1*04:01	693	707	3.69	>0.1
1313	AVVLSVASFILLSMV	HLA-DRB1*11:01	693	707	5.12	>0.1
54	VVLSVASFILLSMVG	HLA-DRB1*07:01	694	708	0.32	>0.1
91	VVLSVASFILLSMVG	HLA-DRB1*03:01	694	708	0.53	>0.1
513	VVLSVASFILLSMVG	HLA-DRB1*15:01	694	708	2.3	>0.1

644	VVLSVASFILLSMVG	HLA-DRB1*13:01	694	708	2.65	>0.1
1039	VVLSVASFILLSMVG	HLA-DRB1*04:01	694	708	4.23	>0.1
1190	VVLSVASFILLSMVG	HLA-DRB1*11:01	694	708	4.7	>0.1
89	VLSVASFILLSMVGV	HLA-DRB1*07:01	695	709	0.51	>0.1
305	VLSVASFILLSMVGV	HLA-DRB1*15:01	695	709	1.33	>0.1
645	VLSVASFILLSMVGV	HLA-DRB1*13:01	695	709	2.65	>0.1
1061	VLSVASFILLSMVGV	HLA-DRB1*11:01	695	709	4.33	>0.1
1091	VLSVASFILLSMVGV	HLA-DRB1*04:01	695	709	4.42	>0.1
1290	VLSVASFILLSMVGV	HLA-DRB1*01:01	695	709	5.03	>0.1
2445	VLSVASFILLSMVGV	HLA-DRB1*03:01	695	709	9.56	>0.1

2512	VLSVASFILLSMVGV	HLA-DRB1*08:01	695	709	9.89	Not predicted
171	LSVASFILLSMVGVA	HLA-DRB1*03:01	696	710	0.89	>0.1
456	LSVASFILLSMVGVA	HLA-DRB1*15:01	696	710	2.03	>0.1
646	LSVASFILLSMVGVA	HLA-DRB1*13:01	696	710	2.65	>0.1
875	LSVASFILLSMVGVA	HLA-DRB1*04:01	696	710	3.57	>0.1
925	LSVASFILLSMVGVA	HLA-DRB1*01:01	696	710	3.8	>0.1
988	LSVASFILLSMVGVA	HLA-DRB1*11:01	696	710	4.02	>0.1
1995	LSVASFILLSMVGVA	HLA-DRB1*08:01	696	710	7.82	Not predicted
50	SVASFILLSMVGVAV	HLA-DRB1*01:01	697	711	0.28	>0.1
172	SVASFILLSMVGVAV	HLA-DRB1*03:01	697	711	0.89	>0.1

339	SVASFILLSMVGVAV	HLA-DRB1*15:01	697	711	1.4	>0.1
647	SVASFILLSMVGVAV	HLA-DRB1*13:01	697	711	2.65	>0.1
876	SVASFILLSMVGVAV	HLA-DRB1*04:01	697	711	3.57	>0.1
989	SVASFILLSMVGVAV	HLA-DRB1*11:01	697	711	4.02	>0.1
1996	SVASFILLSMVGVAV	HLA-DRB1*08:01	697	711	7.82	Not predicted
51	VASFILLSMVGVAVG	HLA-DRB1*01:01	698	712	0.28	0.0069
173	VASFILLSMVGVAVG	HLA-DRB1*03:01	698	712	0.89	>0.1
363	VASFILLSMVGVAVG	HLA-DRB1*15:01	698	712	1.5	0.0165
648	VASFILLSMVGVAVG	HLA-DRB1*13:01	698	712	2.65	>0.1
816	VASFILLSMVGVAVG	HLA-DRB1*08:01	698	712	3.31	Not predicted

877	VASFILLSMVGVAVG	HLA-DRB1*04:01	698	712	3.57	>0.1
990	VASFILLSMVGVAVG	HLA-DRB1*11:01	698	712	4.02	>0.1
52	ASFILLSMVGVAVGM	HLA-DRB1*01:01	699	713	0.28	>0.1
174	ASFILLSMVGVAVGM	HLA-DRB1*03:01	699	713	0.89	>0.1
626	ASFILLSMVGVAVGM	HLA-DRB1*15:01	699	713	2.61	>0.1
817	ASFILLSMVGVAVGM	HLA-DRB1*08:01	699	713	3.31	Not predicted
878	ASFILLSMVGVAVGM	HLA-DRB1*04:01	699	713	3.57	>0.1
991	ASFILLSMVGVAVGM	HLA-DRB1*11:01	699	713	4.02	0.0153
1571	ASFILLSMVGVAVGM	HLA-DRB1*13:01	699	713	6.36	>0.1
102	SFILLSMVGVAVGMC	HLA-DRB1*01:01	700	714	0.6	>0.1

175	SFILLSMVGVAVGMC	HLA-DRB1*03:01	700	714	0.89	0.0134
237	SFILLSMVGVAVGMC	HLA-DRB1*11:01	700	714	1.08	>0.1
537	SFILLSMVGVAVGMC	HLA-DRB1*08:01	700	714	2.37	Not predicted
874	SFILLSMVGVAVGMC	HLA-DRB1*15:01	700	714	3.56	>0.1
879	SFILLSMVGVAVGMC	HLA-DRB1*04:01	700	714	3.57	>0.1
1572	SFILLSMVGVAVGMC	HLA-DRB1*13:01	700	714	6.36	>0.1
103	FILLSMVGVAVGMCM	HLA-DRB1*01:01	701	715	0.6	>0.1
238	FILLSMVGVAVGMCM	HLA-DRB1*11:01	701	715	1.08	>0.1
424	FILLSMVGVAVGMCM	HLA-DRB1*03:01	701	715	1.78	>0.1
538	FILLSMVGVAVGMCM	HLA-DRB1*08:01	701	715	2.37	Not predicted

880	FILLSMVGVAVGMCM	HLA-DRB1*04:01	701	715	3.57	>0.1
975	FILLSMVGVAVGMCM	HLA-DRB1*15:01	701	715	3.91	>0.1
1573	FILLSMVGVAVGMCM	HLA-DRB1*13:01	701	715	6.36	>0.1
434	ILLSMVGVAVGMCMC	HLA-DRB1*03:01	702	716	1.84	>0.1
539	ILLSMVGVAVGMCMC	HLA-DRB1*08:01	702	716	2.37	Not predicted
796	ILLSMVGVAVGMCMC	HLA-DRB1*01:01	702	716	3.24	>0.1
881	ILLSMVGVAVGMCMC	HLA-DRB1*04:01	702	716	3.57	>0.1
1450	ILLSMVGVAVGMCMC	HLA-DRB1*11:01	702	716	5.67	>0.1
1574	ILLSMVGVAVGMCMC	HLA-DRB1*13:01	702	716	6.36	>0.1
540	LLSMVGVAVGMCMCA	HLA-DRB1*08:01	703	717	2.37	Not predicted

914	LLSMVGVAVGMCMCA	HLA-DRB1*01:01	703	717	3.72	>0.1
1575	LLSMVGVAVGMCMCA	HLA-DRB1*13:01	703	717	6.36	>0.1
1801	LLSMVGVAVGMCMCA	HLA-DRB1*11:01	703	717	6.95	>0.1
2309	LLSMVGVAVGMCMCA	HLA-DRB1*04:01	703	717	9.08	>0.1
541	LSMVGVAVGMCMCAR	HLA-DRB1*08:01	704	718	2.37	Not predicted
1576	LSMVGVAVGMCMCAR	HLA-DRB1*13:01	704	718	6.36	>0.1
542	SMVGVAVGMCMCARR	HLA-DRB1*08:01	705	719	2.37	Not predicted
872	SMVGVAVGMCMCARR	HLA-DRB1*13:01	705	719	3.55	>0.1
543	MVGVAVGMCMCARRR	HLA-DRB1*08:01	706	720	2.37	Not predicted
873	MVGVAVGMCMCARRR	HLA-DRB1*13:01	706	720	3.55	>0.1

224	VGVAVGMCMCARRRC	HLA-DRB1*13:01	707	721	1.05	>0.1
1176	VGVAVGMCMCARRRC	HLA-DRB1*11:01	707	721	4.66	>0.1
1964	VGVAVGMCMCARRRC	HLA-DRB1*08:01	707	721	7.81	Not predicted
225	GVAVGMCMCARRRCI	HLA-DRB1*13:01	708	722	1.05	>0.1
1177	GVAVGMCMCARRRCI	HLA-DRB1*11:01	708	722	4.66	>0.1
1965	GVAVGMCMCARRRCI	HLA-DRB1*08:01	708	722	7.81	Not predicted
226	VAVGMCMCARRRCIT	HLA-DRB1*13:01	709	723	1.05	>0.1
818	VAVGMCMCARRRCIT	HLA-DRB1*08:01	709	723	3.31	Not predicted
1178	VAVGMCMCARRRCIT	HLA-DRB1*11:01	709	723	4.66	>0.1
2304	VAVGMCMCARRRCIT	HLA-DRB1*03:01	709	723	9.05	>0.1

227	AVGMCMCARRRCITP	HLA-DRB1*13:01	710	724	1.05	>0.1
819	AVGMCMCARRRCITP	HLA-DRB1*08:01	710	724	3.31	Not predicted
1179	AVGMCMCARRRCITP	HLA-DRB1*11:01	710	724	4.66	>0.1
228	VGMCMCARRRCITPY	HLA-DRB1*13:01	711	725	1.05	>0.1
820	VGMCMCARRRCITPY	HLA-DRB1*08:01	711	725	3.31	Not predicted
1180	VGMCMCARRRCITPY	HLA-DRB1*11:01	711	725	4.66	>0.1
229	GMCMCARRRCITPYE	HLA-DRB1*13:01	712	726	1.05	>0.1
821	GMCMCARRRCITPYE	HLA-DRB1*08:01	712	726	3.31	Not predicted
1181	GMCMCARRRCITPYE	HLA-DRB1*11:01	712	726	4.66	0.0366
230	MCMCARRRCITPYEL	HLA-DRB1*13:01	713	727	1.05	>0.1

822	MCMCARRRCITPYEL	HLA-DRB1*08:01	713	727	3.31	Not predicted
1308	MCMCARRRCITPYEL	HLA-DRB1*11:01	713	727	5.07	>0.1
823	CMCARRRCITPYELT	HLA-DRB1*08:01	714	728	3.31	Not predicted
824	MCARRRCITPYELTP	HLA-DRB1*08:01	715	729	3.31	Not predicted
169	RRCITPYELTPGATV	HLA-DRB1*07:01	719	733	0.88	>0.1
2520	RRCITPYELTPGATV	HLA-DRB1*01:01	719	733	9.9	>0.1
245	RCITPYELTPGATVP	HLA-DRB1*07:01	720	734	1.12	>0.1
257	CITPYELTPGATVPF	HLA-DRB1*07:01	721	735	1.14	>0.1
1623	CITPYELTPGATVPF	HLA-DRB1*01:01	721	735	6.42	>0.1
232	ITPYELTPGATVPFL	HLA-DRB1*07:01	722	736	1.08	>0.1

1462	ITPYELTPGATVPFL	HLA-DRB1*01:01	722	736	5.79	>0.1
391	TPYELTPGATVPFLL	HLA-DRB1*07:01	723	737	1.64	>0.1
1541	TPYELTPGATVPFLL	HLA-DRB1*01:01	723	737	6.21	>0.1
599	PYELTPGATVPFLLS	HLA-DRB1*07:01	724	738	2.57	>0.1
918	YELTPGATVPFLLSL	HLA-DRB1*07:01	725	739	3.73	>0.1
2252	LTPGATVPFLLSLIC	HLA-DRB1*11:01	727	741	8.86	>0.1
2370	TPGATVPFLLSLICC	HLA-DRB1*11:01	728	742	9.23	>0.1
651	PGATVPFLLSLICCI	HLA-DRB1*03:01	729	743	2.71	>0.1
1104	PGATVPFLLSLICCI	HLA-DRB1*08:01	729	743	4.47	Not predicted
1464	PGATVPFLLSLICCI	HLA-DRB1*11:01	729	743	5.8	>0.1

1612	PGATVPFLLSLICCI	HLA-DRB1*13:01	729	743	6.41	>0.1
652	GATVPFLLSLICCIR	HLA-DRB1*03:01	730	744	2.71	>0.1
1105	GATVPFLLSLICCIR	HLA-DRB1*08:01	730	744	4.47	Not predicted
1161	GATVPFLLSLICCIR	HLA-DRB1*13:01	730	744	4.62	>0.1
1369	GATVPFLLSLICCIR	HLA-DRB1*11:01	730	744	5.36	>0.1
2499	GATVPFLLSLICCIR	HLA-DRB1*15:01	730	744	9.79	>0.1
653	ATVPFLLSLICCIRT	HLA-DRB1*03:01	731	745	2.71	>0.1
861	ATVPFLLSLICCIRT	HLA-DRB1*11:01	731	745	3.43	>0.1
1106	ATVPFLLSLICCIRT	HLA-DRB1*08:01	731	745	4.47	Not predicted
1162	ATVPFLLSLICCIRT	HLA-DRB1*13:01	731	745	4.62	>0.1

2377	ATVPFLLSLICCIRT	HLA-DRB1*15:01	731	745	9.34	>0.1
654	TVPFLLSLICCIRTA	HLA-DRB1*03:01	732	746	2.71	>0.1
995	TVPFLLSLICCIRTA	HLA-DRB1*11:01	732	746	4.06	>0.1
1107	TVPFLLSLICCIRTA	HLA-DRB1*08:01	732	746	4.47	Not predicted
1163	TVPFLLSLICCIRTA	HLA-DRB1*13:01	732	746	4.62	>0.1
495	VPFLLSLICCIRTAK	HLA-DRB1*11:01	733	747	2.21	>0.1
655	VPFLLSLICCIRTAK	HLA-DRB1*03:01	733	747	2.71	>0.1
1108	VPFLLSLICCIRTAK	HLA-DRB1*08:01	733	747	4.47	Not predicted
1164	VPFLLSLICCIRTAK	HLA-DRB1*13:01	733	747	4.62	>0.1
556	PFLLSLICCIRTAKA	HLA-DRB1*11:01	734	748	2.41	>0.1

807	PFLLSLICCIRTAKA	HLA-DRB1*04:01	734	748	3.28	>0.1
979	PFLLSLICCIRTAKA	HLA-DRB1*03:01	734	748	3.95	>0.1
1109	PFLLSLICCIRTAKA	HLA-DRB1*08:01	734	748	4.47	Not predicted
1165	PFLLSLICCIRTAKA	HLA-DRB1*13:01	734	748	4.62	>0.1
2193	PFLLSLICCIRTAKA	HLA-DRB1*15:01	734	748	8.52	>0.1
557	FLLSLICCIRTAKAA	HLA-DRB1*11:01	735	749	2.41	>0.1
763	FLLSLICCIRTAKAA	HLA-DRB1*04:01	735	749	3.21	>0.1
1036	FLLSLICCIRTAKAA	HLA-DRB1*03:01	735	749	4.22	>0.1
1110	FLLSLICCIRTAKAA	HLA-DRB1*08:01	735	749	4.47	Not predicted
1166	FLLSLICCIRTAKAA	HLA-DRB1*13:01	735	749	4.62	>0.1

1577	FLLSLICCIRTAKAA	HLA-DRB1*15:01	735	749	6.38	>0.1
833	LLSLICCIRTAKAAT	HLA-DRB1*04:01	736	750	3.32	>0.1
1167	LLSLICCIRTAKAAT	HLA-DRB1*13:01	736	750	4.62	>0.1
1353	LLSLICCIRTAKAAT	HLA-DRB1*15:01	736	750	5.22	>0.1
1382	LLSLICCIRTAKAAT	HLA-DRB1*11:01	736	750	5.43	>0.1
292	LSLICCIRTAKAATY	HLA-DRB1*04:01	737	751	1.23	>0.1
778	LSLICCIRTAKAATY	HLA-DRB1*13:01	737	751	3.23	>0.1
1085	LSLICCIRTAKAATY	HLA-DRB1*03:01	737	751	4.37	>0.1
1315	LSLICCIRTAKAATY	HLA-DRB1*15:01	737	751	5.12	>0.1
1336	LSLICCIRTAKAATY	HLA-DRB1*08:01	737	751	5.17	Not predicted

1383	LSLICCIRTAKAATY	HLA-DRB1*11:01	737	751	5.43	0.0494
2415	LSLICCIRTAKAATY	HLA-DRB1*07:01	737	751	9.46	>0.1
414	SLICCIRTAKAATYQ	HLA-DRB1*04:01	738	752	1.74	>0.1
779	SLICCIRTAKAATYQ	HLA-DRB1*13:01	738	752	3.23	>0.1
1086	SLICCIRTAKAATYQ	HLA-DRB1*03:01	738	752	4.37	>0.1
1337	SLICCIRTAKAATYQ	HLA-DRB1*08:01	738	752	5.17	Not predicted
1384	SLICCIRTAKAATYQ	HLA-DRB1*11:01	738	752	5.43	>0.1
2416	SLICCIRTAKAATYQ	HLA-DRB1*07:01	738	752	9.46	>0.1
587	LICCIRTAKAATYQE	HLA-DRB1*04:01	739	753	2.53	>0.1
780	LICCIRTAKAATYQE	HLA-DRB1*13:01	739	753	3.23	>0.1

1087	LICCIRTAKAATYQE	HLA-DRB1*03:01	739	753	4.37	0.0938
1338	LICCIRTAKAATYQE	HLA-DRB1*08:01	739	753	5.17	Not predicted
1385	LICCIRTAKAATYQE	HLA-DRB1*11:01	739	753	5.43	>0.1
2417	LICCIRTAKAATYQE	HLA-DRB1*07:01	739	753	9.46	>0.1
671	ICCIRTAKAATYQEA	HLA-DRB1*04:01	740	754	2.78	>0.1
781	ICCIRTAKAATYQEA	HLA-DRB1*13:01	740	754	3.23	>0.1
1088	ICCIRTAKAATYQEA	HLA-DRB1*03:01	740	754	4.37	>0.1
1339	ICCIRTAKAATYQEA	HLA-DRB1*08:01	740	754	5.17	Not predicted
1386	ICCIRTAKAATYQEA	HLA-DRB1*11:01	740	754	5.43	>0.1
2418	ICCIRTAKAATYQEA	HLA-DRB1*07:01	740	754	9.46	>0.1

782	CCIRTAKAATYQEAA	HLA-DRB1*13:01	741	755	3.23	>0.1
1089	CCIRTAKAATYQEAA	HLA-DRB1*03:01	741	755	4.37	>0.1
1340	CCIRTAKAATYQEAA	HLA-DRB1*08:01	741	755	5.17	Not predicted
1924	CCIRTAKAATYQEAA	HLA-DRB1*11:01	741	755	7.59	>0.1
2069	CCIRTAKAATYQEAA	HLA-DRB1*04:01	741	755	7.96	>0.1
2419	CCIRTAKAATYQEAA	HLA-DRB1*07:01	741	755	9.46	>0.1
783	CIRTAKAATYQEAAV	HLA-DRB1*13:01	742	756	3.23	>0.1
1341	CIRTAKAATYQEAAV	HLA-DRB1*08:01	742	756	5.17	Not predicted
1544	CIRTAKAATYQEAAV	HLA-DRB1*03:01	742	756	6.24	>0.1
1958	CIRTAKAATYQEAAV	HLA-DRB1*11:01	742	756	7.77	>0.1

2070	CIRTAKAATYQEAAV	HLA-DRB1*04:01	742	756	7.96	>0.1
784	IRTAKAATYQEAAVY	HLA-DRB1*13:01	743	757	3.23	>0.1
1342	IRTAKAATYQEAAVY	HLA-DRB1*08:01	743	757	5.17	Not predicted
1526	IRTAKAATYQEAAVY	HLA-DRB1*03:01	743	757	6.09	>0.1
2071	IRTAKAATYQEAAVY	HLA-DRB1*04:01	743	757	7.96	>0.1
1686	EAAVYLWNEQQPLFW	HLA-DRB1*03:01	753	767	6.7	>0.1
1925	EAAVYLWNEQQPLFW	HLA-DRB1*11:01	753	767	7.59	>0.1
973	AAVYLWNEQQPLFWM	HLA-DRB1*03:01	754	768	3.9	0.018
970	AVYLWNEQQPLFWMQ	HLA-DRB1*03:01	755	769	3.88	>0.1
1520	VYLWNEQQPLFWMQA	HLA-DRB1*03:01	756	770	6.03	>0.1

2524	YLWNEQQPLFWMQAL	HLA-DRB1*03:01	757	771	9.93	>0.1
1613	WNEQQPLFWMQALIP	HLA-DRB1*13:01	759	773	6.41	>0.1
2206	WNEQQPLFWMQALIP	HLA-DRB1*04:01	759	773	8.59	>0.1
2527	WNEQQPLFWMQALIP	HLA-DRB1*11:01	759	773	9.98	>0.1
1459	NEQQPLFWMQALIPL	HLA-DRB1*11:01	760	774	5.7	>0.1
1614	NEQQPLFWMQALIPL	HLA-DRB1*13:01	760	774	6.41	>0.1
2247	NEQQPLFWMQALIPL	HLA-DRB1*04:01	760	774	8.84	>0.1
547	EQQPLFWMQALIPLA	HLA-DRB1*11:01	761	775	2.4	>0.1
1615	EQQPLFWMQALIPLA	HLA-DRB1*13:01	761	775	6.41	>0.1
1914	EQQPLFWMQALIPLA	HLA-DRB1*04:01	761	775	7.51	>0.1

2124	EQQPLFWMQALIPLA	HLA-DRB1*08:01	761	775	8.32	Not predicted
2463	EQQPLFWMQALIPLA	HLA-DRB1*03:01	761	775	9.61	>0.1
2501	EQQPLFWMQALIPLA	HLA-DRB1*01:01	761	775	9.82	>0.1
144	QQPLFWMQALIPLAA	HLA-DRB1*11:01	762	776	0.8	>0.1
673	QQPLFWMQALIPLAA	HLA-DRB1*04:01	762	776	2.79	>0.1
785	QQPLFWMQALIPLAA	HLA-DRB1*13:01	762	776	3.23	>0.1
797	QQPLFWMQALIPLAA	HLA-DRB1*01:01	762	776	3.24	>0.1
1593	QQPLFWMQALIPLAA	HLA-DRB1*08:01	762	776	6.39	Not predicted
1829	QQPLFWMQALIPLAA	HLA-DRB1*03:01	762	776	7.21	>0.1
134	**QPLFWMQALIPLAAL**	**HLA-DRB1*11:01**	**763**	**777**	0.72	**>0.1**

664	**QPLFWMQALIPLAAL**	**HLA-DRB1*01:01**	**763**	**777**	2.74	**>0.1**
674	**QPLFWMQALIPLAAL**	**HLA-DRB1*04:01**	**763**	**777**	2.79	**>0.1**
786	**QPLFWMQALIPLAAL**	**HLA-DRB1*13:01**	**763**	**777**	3.23	**>0.1**
1594	QPLFWMQALIPLAAL	HLA-DRB1*08:01	763	777	6.39	Not predicted
1830	**QPLFWMQALIPLAAL**	**HLA-DRB1*03:01**	**763**	**777**	7.21	**0.018**
135	PLFWMQALIPLAALI	HLA-DRB1*11:01	764	778	0.72	>0.1
396	PLFWMQALIPLAALI	HLA-DRB1*01:01	764	778	1.71	>0.1
675	PLFWMQALIPLAALI	HLA-DRB1*04:01	764	778	2.79	>0.1
787	PLFWMQALIPLAALI	HLA-DRB1*13:01	764	778	3.23	>0.1
1595	PLFWMQALIPLAALI	HLA-DRB1*08:01	764	778	6.39	Not predicted

1831	PLFWMQALIPLAALI	HLA-DRB1*03:01	764	778	7.21	>0.1
58	LFWMQALIPLAALIV	HLA-DRB1*11:01	765	779	0.43	>0.1
104	LFWMQALIPLAALIV	HLA-DRB1*01:01	765	779	0.6	0.00195
247	LFWMQALIPLAALIV	HLA-DRB1*15:01	765	779	1.13	>0.1
676	LFWMQALIPLAALIV	HLA-DRB1*04:01	765	779	2.79	>0.1
788	LFWMQALIPLAALIV	HLA-DRB1*13:01	765	779	3.23	>0.1
1596	LFWMQALIPLAALIV	HLA-DRB1*08:01	765	779	6.39	Not predicted
1832	LFWMQALIPLAALIV	HLA-DRB1*03:01	765	779	7.21	>0.1
57	FWMQALIPLAALIVL	HLA-DRB1*01:01	766	780	0.42	>0.1
59	FWMQALIPLAALIVL	HLA-DRB1*11:01	766	780	0.43	0.0265

248	FWMQALIPLAALIVL	HLA-DRB1*15:01	766	780	1.13	>0.1
677	FWMQALIPLAALIVL	HLA-DRB1*04:01	766	780	2.79	>0.1
789	FWMQALIPLAALIVL	HLA-DRB1*13:01	766	780	3.23	>0.1
1597	FWMQALIPLAALIVL	HLA-DRB1*08:01	766	780	6.39	Not predicted
2074	FWMQALIPLAALIVL	HLA-DRB1*03:01	766	780	7.99	>0.1
67	WMQALIPLAALIVLC	HLA-DRB1*11:01	767	781	0.44	>0.1
105	WMQALIPLAALIVLC	HLA-DRB1*01:01	767	781	0.6	>0.1
249	WMQALIPLAALIVLC	HLA-DRB1*15:01	767	781	1.13	>0.1
790	WMQALIPLAALIVLC	HLA-DRB1*13:01	767	781	3.23	>0.1
1031	WMQALIPLAALIVLC	HLA-DRB1*04:01	767	781	4.17	>0.1

1598	WMQALIPLAALIVLC	HLA-DRB1*08:01	767	781	6.39	Not predicted
2223	WMQALIPLAALIVLC	HLA-DRB1*03:01	767	781	8.79	>0.1
68	MQALIPLAALIVLCN	HLA-DRB1*11:01	768	782	0.44	>0.1
106	MQALIPLAALIVLCN	HLA-DRB1*01:01	768	782	0.6	>0.1
250	MQALIPLAALIVLCN	HLA-DRB1*15:01	768	782	1.13	>0.1
791	MQALIPLAALIVLCN	HLA-DRB1*13:01	768	782	3.23	>0.1
994	MQALIPLAALIVLCN	HLA-DRB1*04:01	768	782	4.05	>0.1
1511	MQALIPLAALIVLCN	HLA-DRB1*08:01	768	782	5.97	Not predicted
107	QALIPLAALIVLCNC	HLA-DRB1*01:01	769	783	0.6	>0.1
251	QALIPLAALIVLCNC	HLA-DRB1*15:01	769	783	1.13	>0.1

303	QALIPLAALIVLCNC	HLA-DRB1*11:01	769	783	1.33	>0.1
792	QALIPLAALIVLCNC	HLA-DRB1*13:01	769	783	3.23	>0.1
1419	QALIPLAALIVLCNC	HLA-DRB1*04:01	769	783	5.5	>0.1
1512	QALIPLAALIVLCNC	HLA-DRB1*08:01	769	783	5.97	Not predicted
372	ALIPLAALIVLCNCL	HLA-DRB1*11:01	770	784	1.53	>0.1
793	ALIPLAALIVLCNCL	HLA-DRB1*13:01	770	784	3.23	>0.1
838	ALIPLAALIVLCNCL	HLA-DRB1*15:01	770	784	3.35	>0.1
865	ALIPLAALIVLCNCL	HLA-DRB1*01:01	770	784	3.49	>0.1
1513	ALIPLAALIVLCNCL	HLA-DRB1*08:01	770	784	5.97	Not predicted
1910	ALIPLAALIVLCNCL	HLA-DRB1*04:01	770	784	7.49	>0.1

304	LIPLAALIVLCNCLR	HLA-DRB1*11:01	771	785	1.33	>0.1
794	LIPLAALIVLCNCLR	HLA-DRB1*13:01	771	785	3.23	>0.1
806	LIPLAALIVLCNCLR	HLA-DRB1*15:01	771	785	3.26	>0.1
1514	LIPLAALIVLCNCLR	HLA-DRB1*08:01	771	785	5.97	Not predicted
1631	LIPLAALIVLCNCLR	HLA-DRB1*04:01	771	785	6.47	>0.1
1937	LIPLAALIVLCNCLR	HLA-DRB1*03:01	771	785	7.6	>0.1
262	IPLAALIVLCNCLRL	HLA-DRB1*13:01	772	786	1.19	>0.1
293	IPLAALIVLCNCLRL	HLA-DRB1*11:01	772	786	1.23	>0.1
485	IPLAALIVLCNCLRL	HLA-DRB1*03:01	772	786	2.19	>0.1
882	IPLAALIVLCNCLRL	HLA-DRB1*08:01	772	786	3.58	Not predicted

1711	IPLAALIVLCNCLRL	HLA-DRB1*04:01	772	786	6.82	>0.1
1917	IPLAALIVLCNCLRL	HLA-DRB1*15:01	772	786	7.55	>0.1
263	PLAALIVLCNCLRLL	HLA-DRB1*13:01	773	787	1.19	>0.1
376	PLAALIVLCNCLRLL	HLA-DRB1*11:01	773	787	1.55	>0.1
486	PLAALIVLCNCLRLL	HLA-DRB1*03:01	773	787	2.19	>0.1
883	PLAALIVLCNCLRLL	HLA-DRB1*08:01	773	787	3.58	Not predicted
1702	PLAALIVLCNCLRLL	HLA-DRB1*15:01	773	787	6.74	>0.1
215	LAALIVLCNCLRLLP	HLA-DRB1*11:01	774	788	0.98	>0.1
264	LAALIVLCNCLRLLP	HLA-DRB1*13:01	774	788	1.19	>0.1
487	LAALIVLCNCLRLLP	HLA-DRB1*03:01	774	788	2.19	>0.1

884	LAALIVLCNCLRLLP	HLA-DRB1*08:01	774	788	3.58	Not predicted
1705	LAALIVLCNCLRLLP	HLA-DRB1*15:01	774	788	6.78	>0.1
216	AALIVLCNCLRLLPC	HLA-DRB1*11:01	775	789	0.98	>0.1
265	AALIVLCNCLRLLPC	HLA-DRB1*13:01	775	789	1.19	>0.1
488	AALIVLCNCLRLLPC	HLA-DRB1*03:01	775	789	2.19	>0.1
885	AALIVLCNCLRLLPC	HLA-DRB1*08:01	775	789	3.58	Not predicted
266	ALIVLCNCLRLLPCC	HLA-DRB1*13:01	776	790	1.19	>0.1
269	ALIVLCNCLRLLPCC	HLA-DRB1*11:01	776	790	1.2	>0.1
489	ALIVLCNCLRLLPCC	HLA-DRB1*03:01	776	790	2.19	0.0444
886	ALIVLCNCLRLLPCC	HLA-DRB1*08:01	776	790	3.58	Not predicted

267	LIVLCNCLRLLPCCC	HLA-DRB1*13:01	777	791	1.19	>0.1
301	LIVLCNCLRLLPCCC	HLA-DRB1*11:01	777	791	1.32	>0.1
597	LIVLCNCLRLLPCCC	HLA-DRB1*03:01	777	791	2.57	>0.1
887	LIVLCNCLRLLPCCC	HLA-DRB1*08:01	777	791	3.58	Not predicted
268	IVLCNCLRLLPCCCK	HLA-DRB1*13:01	778	792	1.19	>0.1
437	IVLCNCLRLLPCCCK	HLA-DRB1*11:01	778	792	1.87	>0.1
825	IVLCNCLRLLPCCCK	HLA-DRB1*08:01	778	792	3.31	Not predicted
1252	IVLCNCLRLLPCCCK	HLA-DRB1*03:01	778	792	4.88	>0.1
277	VLCNCLRLLPCCCKM	HLA-DRB1*13:01	779	793	1.2	>0.1
826	VLCNCLRLLPCCCKM	HLA-DRB1*08:01	779	793	3.31	Not predicted

1365	VLCNCLRLLPCCCKM	HLA-DRB1*11:01	779	793	5.25	0.0417
278	LCNCLRLLPCCCKML	HLA-DRB1*13:01	780	794	1.2	>0.1
827	LCNCLRLLPCCCKML	HLA-DRB1*08:01	780	794	3.31	Not predicted
1634	LCNCLRLLPCCCKML	HLA-DRB1*11:01	780	794	6.5	>0.1
279	CNCLRLLPCCCKMLT	HLA-DRB1*13:01	781	795	1.2	>0.1
828	CNCLRLLPCCCKMLT	HLA-DRB1*08:01	781	795	3.31	Not predicted
980	CNCLRLLPCCCKMLT	HLA-DRB1*11:01	781	795	3.95	>0.1
280	NCLRLLPCCCKMLTF	HLA-DRB1*13:01	782	796	1.2	>0.1
829	NCLRLLPCCCKMLTF	HLA-DRB1*08:01	782	796	3.31	Not predicted
961	NCLRLLPCCCKMLTF	HLA-DRB1*11:01	782	796	3.87	>0.1

281	CLRLLPCCCKMLTFL	HLA-DRB1*13:01	783	797	1.2	>0.1
830	CLRLLPCCCKMLTFL	HLA-DRB1*08:01	783	797	3.31	Not predicted
1260	CLRLLPCCCKMLTFL	HLA-DRB1*11:01	783	797	4.94	>0.1
282	LRLLPCCCKMLTFLA	HLA-DRB1*13:01	784	798	1.2	>0.1
831	LRLLPCCCKMLTFLA	HLA-DRB1*08:01	784	798	3.31	Not predicted
962	LRLLPCCCKMLTFLA	HLA-DRB1*11:01	784	798	3.87	>0.1
2295	RLLPCCCKMLTFLAV	HLA-DRB1*13:01	785	799	9.03	>0.1
2317	RLLPCCCKMLTFLAV	HLA-DRB1*11:01	785	799	9.09	>0.1
2296	LLPCCCKMLTFLAVL	HLA-DRB1*13:01	786	800	9.03	>0.1
2318	LLPCCCKMLTFLAVL	HLA-DRB1*11:01	786	800	9.09	>0.1

555	LPCCCKMLTFLAVLS	HLA-DRB1*13:01	787	801	2.4	>0.1
862	LPCCCKMLTFLAVLS	HLA-DRB1*15:01	787	801	3.43	>0.1
1542	LPCCCKMLTFLAVLS	HLA-DRB1*11:01	787	801	6.23	>0.1
2526	LPCCCKMLTFLAVLS	HLA-DRB1*04:01	787	801	9.97	>0.1
120	PCCCKMLTFLAVLSV	HLA-DRB1*11:01	788	802	0.7	>0.1
406	PCCCKMLTFLAVLSV	HLA-DRB1*13:01	788	802	1.72	>0.1
523	PCCCKMLTFLAVLSV	HLA-DRB1*15:01	788	802	2.37	>0.1
808	PCCCKMLTFLAVLSV	HLA-DRB1*04:01	788	802	3.3	>0.1
1706	PCCCKMLTFLAVLSV	HLA-DRB1*07:01	788	802	6.79	>0.1
1731	PCCCKMLTFLAVLSV	HLA-DRB1*08:01	788	802	6.87	Not predicted

121	CCCKMLTFLAVLSVG	HLA-DRB1*11:01	789	803	0.7	>0.1
407	CCCKMLTFLAVLSVG	HLA-DRB1*13:01	789	803	1.72	>0.1
567	CCCKMLTFLAVLSVG	HLA-DRB1*15:01	789	803	2.44	>0.1
809	CCCKMLTFLAVLSVG	HLA-DRB1*04:01	789	803	3.3	>0.1
1707	CCCKMLTFLAVLSVG	HLA-DRB1*07:01	789	803	6.79	>0.1
1732	CCCKMLTFLAVLSVG	HLA-DRB1*08:01	789	803	6.87	Not predicted
122	CCKMLTFLAVLSVGA	HLA-DRB1*11:01	790	804	0.7	>0.1
408	CCKMLTFLAVLSVGA	HLA-DRB1*13:01	790	804	1.72	>0.1
522	CCKMLTFLAVLSVGA	HLA-DRB1*01:01	790	804	2.37	0.0000264
524	CCKMLTFLAVLSVGA	HLA-DRB1*15:01	790	804	2.37	0.0302

810	CCKMLTFLAVLSVGA	HLA-DRB1*04:01	790	804	3.3	>0.1
1708	CCKMLTFLAVLSVGA	HLA-DRB1*07:01	790	804	6.79	>0.1
1733	CCKMLTFLAVLSVGA	HLA-DRB1*08:01	790	804	6.87	Not predicted
123	CKMLTFLAVLSVGAH	HLA-DRB1*11:01	791	805	0.7	>0.1
409	CKMLTFLAVLSVGAH	HLA-DRB1*13:01	791	805	1.72	>0.1
426	CKMLTFLAVLSVGAH	HLA-DRB1*01:01	791	805	1.81	>0.1
525	CKMLTFLAVLSVGAH	HLA-DRB1*15:01	791	805	2.37	>0.1
602	CKMLTFLAVLSVGAH	HLA-DRB1*08:01	791	805	2.58	Not predicted
811	CKMLTFLAVLSVGAH	HLA-DRB1*04:01	791	805	3.3	>0.1
1709	CKMLTFLAVLSVGAH	HLA-DRB1*07:01	791	805	6.79	>0.1

124	KMLTFLAVLSVGAHT	HLA-DRB1*11:01	792	806	0.7	0.00659
343	KMLTFLAVLSVGAHT	HLA-DRB1*01:01	792	806	1.43	>0.1
410	KMLTFLAVLSVGAHT	HLA-DRB1*13:01	792	806	1.72	>0.1
526	KMLTFLAVLSVGAHT	HLA-DRB1*15:01	792	806	2.37	>0.1
603	KMLTFLAVLSVGAHT	HLA-DRB1*08:01	792	806	2.58	Not predicted
812	KMLTFLAVLSVGAHT	HLA-DRB1*04:01	792	806	3.3	>0.1
1710	KMLTFLAVLSVGAHT	HLA-DRB1*07:01	792	806	6.79	>0.1
125	MLTFLAVLSVGAHTV	HLA-DRB1*11:01	793	807	0.7	>0.1
142	MLTFLAVLSVGAHTV	HLA-DRB1*01:01	793	807	0.79	>0.1
411	MLTFLAVLSVGAHTV	HLA-DRB1*13:01	793	807	1.72	>0.1

527	MLTFLAVLSVGAHTV	HLA-DRB1*15:01	793	807	2.37	>0.1
604	MLTFLAVLSVGAHTV	HLA-DRB1*08:01	793	807	2.58	Not predicted
769	MLTFLAVLSVGAHTV	HLA-DRB1*07:01	793	807	3.22	0.00475
813	MLTFLAVLSVGAHTV	HLA-DRB1*04:01	793	807	3.3	>0.1
126	LTFLAVLSVGAHTVS	HLA-DRB1*11:01	794	808	0.7	>0.1
295	LTFLAVLSVGAHTVS	HLA-DRB1*01:01	794	808	1.24	>0.1
412	LTFLAVLSVGAHTVS	HLA-DRB1*13:01	794	808	1.72	>0.1
605	LTFLAVLSVGAHTVS	HLA-DRB1*08:01	794	808	2.58	Not predicted
755	LTFLAVLSVGAHTVS	HLA-DRB1*15:01	794	808	3.11	>0.1
814	LTFLAVLSVGAHTVS	HLA-DRB1*04:01	794	808	3.3	>0.1

1125	LTFLAVLSVGAHTVS	HLA-DRB1*07:01	794	808	4.54	>0.1
508	TFLAVLSVGAHTVSA	HLA-DRB1*01:01	795	809	2.27	>0.1
606	TFLAVLSVGAHTVSA	HLA-DRB1*08:01	795	809	2.58	Not predicted
752	TFLAVLSVGAHTVSA	HLA-DRB1*11:01	795	809	3.1	>0.1
1092	TFLAVLSVGAHTVSA	HLA-DRB1*04:01	795	809	4.42	>0.1
1524	TFLAVLSVGAHTVSA	HLA-DRB1*15:01	795	809	6.07	>0.1
1633	TFLAVLSVGAHTVSA	HLA-DRB1*07:01	795	809	6.47	>0.1
509	FLAVLSVGAHTVSAY	HLA-DRB1*01:01	796	810	2.27	>0.1
607	FLAVLSVGAHTVSAY	HLA-DRB1*08:01	796	810	2.58	Not predicted
724	FLAVLSVGAHTVSAY	HLA-DRB1*13:01	796	810	2.93	>0.1

753	FLAVLSVGAHTVSAY	HLA-DRB1*11:01	796	810	3.1	>0.1
1093	FLAVLSVGAHTVSAY	HLA-DRB1*04:01	796	810	4.42	>0.1
1525	FLAVLSVGAHTVSAY	HLA-DRB1*15:01	796	810	6.07	>0.1
1653	FLAVLSVGAHTVSAY	HLA-DRB1*03:01	796	810	6.58	>0.1
2269	FLAVLSVGAHTVSAY	HLA-DRB1*07:01	796	810	8.96	>0.1
608	LAVLSVGAHTVSAYE	HLA-DRB1*08:01	797	811	2.58	Not predicted
725	LAVLSVGAHTVSAYE	HLA-DRB1*13:01	797	811	2.93	>0.1
1203	LAVLSVGAHTVSAYE	HLA-DRB1*01:01	797	811	4.77	>0.1
1654	LAVLSVGAHTVSAYE	HLA-DRB1*03:01	797	811	6.58	>0.1
1940	LAVLSVGAHTVSAYE	HLA-DRB1*11:01	797	811	7.62	>0.1

726	AVLSVGAHTVSAYEH	HLA-DRB1*13:01	798	812	2.93	>0.1
1701	AVLSVGAHTVSAYEH	HLA-DRB1*03:01	798	812	6.74	>0.1
727	VLSVGAHTVSAYEHV	HLA-DRB1*13:01	799	813	2.93	>0.1
1655	VLSVGAHTVSAYEHV	HLA-DRB1*03:01	799	813	6.58	>0.1
728	LSVGAHTVSAYEHVT	HLA-DRB1*13:01	800	814	2.93	>0.1
1698	LSVGAHTVSAYEHVT	HLA-DRB1*03:01	800	814	6.73	>0.1
729	SVGAHTVSAYEHVTV	HLA-DRB1*13:01	801	815	2.93	>0.1
730	VGAHTVSAYEHVTVI	HLA-DRB1*13:01	802	816	2.93	>0.1
1022	AHTVSAYEHVTVIPN	HLA-DRB1*08:01	804	818	4.16	Not predicted
1694	AHTVSAYEHVTVIPN	HLA-DRB1*11:01	804	818	6.71	>0.1

1023	HTVSAYEHVTVIPNT	HLA-DRB1*08:01	805	819	4.16	Not predicted
1669	HTVSAYEHVTVIPNT	HLA-DRB1*11:01	805	819	6.62	>0.1
1024	TVSAYEHVTVIPNTV	HLA-DRB1*08:01	806	820	4.16	Not predicted
1354	TVSAYEHVTVIPNTV	HLA-DRB1*11:01	806	820	5.23	>0.1
854	VSAYEHVTVIPNTVG	HLA-DRB1*11:01	807	821	3.42	>0.1
1025	VSAYEHVTVIPNTVG	HLA-DRB1*08:01	807	821	4.16	Not predicted
1676	VSAYEHVTVIPNTVG	HLA-DRB1*04:01	807	821	6.65	>0.1
855	SAYEHVTVIPNTVGV	HLA-DRB1*11:01	808	822	3.42	>0.1
1026	SAYEHVTVIPNTVGV	HLA-DRB1*08:01	808	822	4.16	Not predicted
1677	SAYEHVTVIPNTVGV	HLA-DRB1*04:01	808	822	6.65	>0.1

866	AYEHVTVIPNTVGVP	HLA-DRB1*11:01	809	823	3.49	>0.1
1027	AYEHVTVIPNTVGVP	HLA-DRB1*08:01	809	823	4.16	Not predicted
2480	AYEHVTVIPNTVGVP	HLA-DRB1*04:01	809	823	9.7	>0.1
867	YEHVTVIPNTVGVPY	HLA-DRB1*11:01	810	824	3.49	>0.1
1028	YEHVTVIPNTVGVPY	HLA-DRB1*08:01	810	824	4.16	Not predicted
2072	YEHVTVIPNTVGVPY	HLA-DRB1*03:01	810	824	7.97	>0.1
2502	YEHVTVIPNTVGVPY	HLA-DRB1*04:01	810	824	9.82	>0.1
1959	EHVTVIPNTVGVPYK	HLA-DRB1*03:01	811	825	7.78	>0.1
2319	EHVTVIPNTVGVPYK	HLA-DRB1*11:01	811	825	9.09	>0.1
1951	HVTVIPNTVGVPYKT	HLA-DRB1*03:01	812	826	7.7	>0.1

1912	VTVIPNTVGVPYKTL	HLA-DRB1*03:01	813	827	7.51	>0.1
**2106**	**TVIPNTVGVPYKTLV**	**HLA-DRB1*03:01**	**814**	**828**	**8.3**	**>0.1**
**1616**	**IPNTVGVPYKTLVNR**	**HLA-DRB1*13:01**	**816**	**830**	**6.41**	**>0.1**
**1617**	**PNTVGVPYKTLVNRP**	**HLA-DRB1*13:01**	**817**	**831**	**6.41**	**>0.1**
**1207**	**NTVGVPYKTLVNRPG**	**HLA-DRB1*04:01**	**818**	**832**	**4.78**	**>0.1**
**1618**	**NTVGVPYKTLVNRPG**	**HLA-DRB1*13:01**	**818**	**832**	**6.41**	**>0.1**
1734	NTVGVPYKTLVNRPG	HLA-DRB1*08:01	818	832	6.87	Not predicted
**1926**	**NTVGVPYKTLVNRPG**	**HLA-DRB1*11:01**	**818**	**832**	**7.59**	**>0.1**
**1208**	**TVGVPYKTLVNRPGY**	**HLA-DRB1*04:01**	**819**	**833**	**4.78**	**>0.1**
**1619**	**TVGVPYKTLVNRPGY**	**HLA-DRB1*13:01**	**819**	**833**	**6.41**	**>0.1**

1735	TVGVPYKTLVNRPGY	HLA-DRB1*08:01	819	833	6.87	Not predicted
**1927**	**TVGVPYKTLVNRPGY**	**HLA-DRB1*11:01**	**819**	**833**	**7.59**	**>0.1**
**910**	**VGVPYKTLVNRPGYS**	**HLA-DRB1*04:01**	**820**	**834**	**3.69**	**>0.1**
**1415**	**VGVPYKTLVNRPGYS**	**HLA-DRB1*11:01**	**820**	**834**	**5.46**	**>0.1**
**1620**	**VGVPYKTLVNRPGYS**	**HLA-DRB1*13:01**	**820**	**834**	**6.41**	**>0.1**
1736	VGVPYKTLVNRPGYS	HLA-DRB1*08:01	820	834	6.87	Not predicted
**2494**	**VGVPYKTLVNRPGYS**	**HLA-DRB1*03:01**	**820**	**834**	**9.73**	**>0.1**
578	GVPYKTLVNRPGYSP	HLA-DRB1*04:01	821	835	2.49	>0.1
871	GVPYKTLVNRPGYSP	HLA-DRB1*11:01	821	835	3.55	>0.1
1430	GVPYKTLVNRPGYSP	HLA-DRB1*08:01	821	835	5.55	Not predicted

1621	GVPYKTLVNRPGYSP	HLA-DRB1*13:01	821	835	6.41	>0.1
804	VPYKTLVNRPGYSPM	HLA-DRB1*04:01	822	836	3.26	>0.1
1075	VPYKTLVNRPGYSPM	HLA-DRB1*11:01	822	836	4.34	>0.1
1431	VPYKTLVNRPGYSPM	HLA-DRB1*08:01	822	836	5.55	Not predicted
1622	VPYKTLVNRPGYSPM	HLA-DRB1*13:01	822	836	6.41	>0.1
1193	PYKTLVNRPGYSPMV	HLA-DRB1*04:01	823	837	4.72	>0.1
1432	PYKTLVNRPGYSPMV	HLA-DRB1*08:01	823	837	5.55	Not predicted
1521	PYKTLVNRPGYSPMV	HLA-DRB1*11:01	823	837	6.04	0.0494
1782	PYKTLVNRPGYSPMV	HLA-DRB1*13:01	823	837	6.91	>0.1
1209	YKTLVNRPGYSPMVL	HLA-DRB1*04:01	824	838	4.78	>0.1

1433	YKTLVNRPGYSPMVL	HLA-DRB1*08:01	824	838	5.55	Not predicted
1783	YKTLVNRPGYSPMVL	HLA-DRB1*13:01	824	838	6.91	>0.1
1928	YKTLVNRPGYSPMVL	HLA-DRB1*11:01	824	838	7.59	>0.1
1434	KTLVNRPGYSPMVLE	HLA-DRB1*08:01	825	839	5.55	Not predicted
1784	KTLVNRPGYSPMVLE	HLA-DRB1*13:01	825	839	6.91	>0.1
1435	TLVNRPGYSPMVLEM	HLA-DRB1*08:01	826	840	5.55	Not predicted
1785	TLVNRPGYSPMVLEM	HLA-DRB1*13:01	826	840	6.91	>0.1
1436	LVNRPGYSPMVLEME	HLA-DRB1*08:01	827	841	5.55	Not predicted
1786	LVNRPGYSPMVLEME	HLA-DRB1*13:01	827	841	6.91	>0.1
2241	VNRPGYSPMVLEMEL	HLA-DRB1*08:01	828	842	8.83	Not predicted

2242	NRPGYSPMVLEMELL	HLA-DRB1*08:01	829	843	8.83	Not predicted
1168	**RPGYSPMVLEMELLS**	**HLA-DRB1*13:01**	**830**	**844**	**4.62**	**>0.1**
1687	**RPGYSPMVLEMELLS**	**HLA-DRB1*03:01**	**830**	**844**	**6.71**	**>0.1**
1981	**RPGYSPMVLEMELLS**	**HLA-DRB1*04:01**	**830**	**844**	**7.82**	**>0.1**
2243	RPGYSPMVLEMELLS	HLA-DRB1*08:01	830	844	8.83	Not predicted
1169	**PGYSPMVLEMELLSV**	**HLA-DRB1*13:01**	**831**	**845**	**4.62**	**>0.1**
1657	**PGYSPMVLEMELLSV**	**HLA-DRB1*04:01**	**831**	**845**	**6.59**	**>0.1**
1688	**PGYSPMVLEMELLSV**	**HLA-DRB1*03:01**	**831**	**845**	**6.71**	**>0.1**
2244	PGYSPMVLEMELLSV	HLA-DRB1*08:01	831	845	8.83	Not predicted
2409	**PGYSPMVLEMELLSV**	**HLA-DRB1*11:01**	**831**	**845**	**9.42**	**>0.1**

1170	**GYSPMVLEMELLSVT**	**HLA-DRB1*13:01**	**832**	**846**	**4.62**	**>0.1**
1689	**GYSPMVLEMELLSVT**	**HLA-DRB1*03:01**	**832**	**846**	**6.71**	**>0.1**
1956	**GYSPMVLEMELLSVT**	**HLA-DRB1*04:01**	**832**	**846**	**7.75**	**>0.1**
2245	GYSPMVLEMELLSVT	HLA-DRB1*08:01	832	846	8.83	Not predicted
1171	**YSPMVLEMELLSVTL**	**HLA-DRB1*13:01**	**833**	**847**	**4.62**	**>0.1**
1275	**YSPMVLEMELLSVTL**	**HLA-DRB1*04:01**	**833**	**847**	**5**	**>0.1**
1527	**YSPMVLEMELLSVTL**	**HLA-DRB1*11:01**	**833**	**847**	**6.1**	**>0.1**
1690	**YSPMVLEMELLSVTL**	**HLA-DRB1*03:01**	**833**	**847**	**6.71**	**>0.1**
2246	YSPMVLEMELLSVTL	HLA-DRB1*08:01	833	847	8.83	Not predicted
1172	**SPMVLEMELLSVTLE**	**HLA-DRB1*13:01**	**834**	**848**	**4.62**	**>0.1**

1309	**SPMVLEMELLSVTLE**	**HLA-DRB1*04:01**	**834**	**848**	**5.08**	**>0.1**
1439	**SPMVLEMELLSVTLE**	**HLA-DRB1*11:01**	**834**	**848**	**5.56**	**>0.1**
1691	SPMVLEMELLSVTLE	HLA-DRB1*03:01	834	848	6.71	0.0111
1737	SPMVLEMELLSVTLE	HLA-DRB1*08:01	834	848	6.87	Not predicted
1173	PMVLEMELLSVTLEP	HLA-DRB1*13:01	835	849	4.62	>0.1
1658	PMVLEMELLSVTLEP	HLA-DRB1*04:01	835	849	6.59	>0.1
1692	PMVLEMELLSVTLEP	HLA-DRB1*03:01	835	849	6.71	>0.1
1738	PMVLEMELLSVTLEP	HLA-DRB1*08:01	835	849	6.87	Not predicted
2228	PMVLEMELLSVTLEP	HLA-DRB1*11:01	835	849	8.82	>0.1
920	**MVLEMELLSVTLEPT**	**HLA-DRB1*04:01**	**836**	**850**	**3.75**	**>0.1**

**1174**	**MVLEMELLSVTLEPT**	**HLA-DRB1*13:01**	**836**	**850**	**4.62**	**>0.1**
**1693**	**MVLEMELLSVTLEPT**	**HLA-DRB1*03:01**	**836**	**850**	**6.71**	**>0.1**
1739	MVLEMELLSVTLEPT	HLA-DRB1*08:01	836	850	6.87	Not predicted
2218	MVLEMELLSVTLEPT	HLA-DRB1*11:01	836	850	8.75	>0.1
1740	VLEMELLSVTLEPTL	HLA-DRB1*08:01	837	851	6.87	Not predicted
1886	VLEMELLSVTLEPTL	HLA-DRB1*13:01	837	851	7.42	>0.1
2076	VLEMELLSVTLEPTL	HLA-DRB1*11:01	837	851	8.04	>0.1
2310	VLEMELLSVTLEPTL	HLA-DRB1*04:01	837	851	9.08	>0.1
2504	VLEMELLSVTLEPTL	HLA-DRB1*03:01	837	851	9.87	>0.1
1741	LEMELLSVTLEPTLS	HLA-DRB1*08:01	838	852	6.87	Not predicted

1887	LEMELLSVTLEPTLS	HLA-DRB1*13:01	838	852	7.42	>0.1
2311	LEMELLSVTLEPTLS	HLA-DRB1*04:01	838	852	9.08	>0.1
711	EMELLSVTLEPTLSL	HLA-DRB1*04:01	839	853	2.91	>0.1
1742	EMELLSVTLEPTLSL	HLA-DRB1*08:01	839	853	6.87	Not predicted
1888	EMELLSVTLEPTLSL	HLA-DRB1*13:01	839	853	7.42	>0.1
1942	EMELLSVTLEPTLSL	HLA-DRB1*03:01	839	853	7.64	>0.1
738	MELLSVTLEPTLSLD	HLA-DRB1*04:01	840	854	3.04	>0.1
1743	MELLSVTLEPTLSLD	HLA-DRB1*08:01	840	854	6.87	Not predicted
1889	MELLSVTLEPTLSLD	HLA-DRB1*13:01	840	854	7.42	>0.1
1943	MELLSVTLEPTLSLD	HLA-DRB1*03:01	840	854	7.64	>0.1

971	ELLSVTLEPTLSLDY	HLA-DRB1*03:01	841	855	3.88	>0.1
1129	ELLSVTLEPTLSLDY	HLA-DRB1*04:01	841	855	4.61	>0.1
2279	ELLSVTLEPTLSLDY	HLA-DRB1*07:01	841	855	8.99	>0.1
465	LLSVTLEPTLSLDYI	HLA-DRB1*03:01	842	856	2.14	>0.1
1251	LLSVTLEPTLSLDYI	HLA-DRB1*04:01	842	856	4.87	>0.1
1883	LLSVTLEPTLSLDYI	HLA-DRB1*07:01	842	856	7.38	>0.1
1037	LSVTLEPTLSLDYIT	HLA-DRB1*03:01	843	857	4.22	>0.1
1815	LSVTLEPTLSLDYIT	HLA-DRB1*04:01	843	857	7.07	>0.1
2356	LSVTLEPTLSLDYIT	HLA-DRB1*07:01	843	857	9.1	>0.1
1955	SVTLEPTLSLDYITC	HLA-DRB1*03:01	844	858	7.75	>0.1

453	VTLEPTLSLDYITCE	HLA-DRB1*03:01	845	859	1.99	>0.1
546	TLEPTLSLDYITCEY	HLA-DRB1*03:01	846	860	2.4	>0.1
521	LEPTLSLDYITCEYK	HLA-DRB1*03:01	847	861	2.35	>0.1
575	EPTLSLDYITCEYKT	HLA-DRB1*03:01	848	862	2.48	0.0831
584	PTLSLDYITCEYKTV	HLA-DRB1*03:01	849	863	2.51	>0.1
896	TLSLDYITCEYKTVI	HLA-DRB1*03:01	850	864	3.65	>0.1
923	LSLDYITCEYKTVIP	HLA-DRB1*03:01	851	865	3.78	>0.1
1489	DYITCEYKTVIPSPY	HLA-DRB1*07:01	854	868	5.97	>0.1
1997	DYITCEYKTVIPSPY	HLA-DRB1*08:01	854	868	7.82	Not predicted
757	YITCEYKTVIPSPYV	HLA-DRB1*07:01	855	869	3.12	>0.1

1998	YITCEYKTVIPSPYV	HLA-DRB1*08:01	855	869	7.82	Not predicted
976	ITCEYKTVIPSPYVK	HLA-DRB1*07:01	856	870	3.93	>0.1
1999	ITCEYKTVIPSPYVK	HLA-DRB1*08:01	856	870	7.82	Not predicted
1130	TCEYKTVIPSPYVKC	HLA-DRB1*07:01	857	871	4.61	>0.1
2000	TCEYKTVIPSPYVKC	HLA-DRB1*08:01	857	871	7.82	Not predicted
2201	TCEYKTVIPSPYVKC	HLA-DRB1*15:01	857	871	8.57	>0.1
1523	CEYKTVIPSPYVKCC	HLA-DRB1*07:01	858	872	6.07	>0.1
2001	CEYKTVIPSPYVKCC	HLA-DRB1*08:01	858	872	7.82	Not predicted
2202	CEYKTVIPSPYVKCC	HLA-DRB1*15:01	858	872	8.57	>0.1
1822	EYKTVIPSPYVKCCG	HLA-DRB1*07:01	859	873	7.12	>0.1

2002	EYKTVIPSPYVKCCG	HLA-DRB1*08:01	859	873	7.82	Not predicted
2203	EYKTVIPSPYVKCCG	HLA-DRB1*15:01	859	873	8.57	>0.1
2003	YKTVIPSPYVKCCGT	HLA-DRB1*08:01	860	874	7.82	Not predicted
2362	YKTVIPSPYVKCCGT	HLA-DRB1*07:01	860	874	9.19	>0.1
2513	TVIPSPYVKCCGTAE	HLA-DRB1*08:01	862	876	9.89	Not predicted
1401	VIPSPYVKCCGTAEC	HLA-DRB1*13:01	863	877	5.45	>0.1
1627	VIPSPYVKCCGTAEC	HLA-DRB1*11:01	863	877	6.44	>0.1
1744	VIPSPYVKCCGTAEC	HLA-DRB1*08:01	863	877	6.87	Not predicted
1402	IPSPYVKCCGTAECK	HLA-DRB1*13:01	864	878	5.45	>0.1
1529	IPSPYVKCCGTAECK	HLA-DRB1*11:01	864	878	6.14	>0.1

1745	IPSPYVKCCGTAECK	HLA-DRB1*08:01	864	878	6.87	Not predicted
1403	PSPYVKCCGTAECKD	HLA-DRB1*13:01	865	879	5.45	>0.1
1553	PSPYVKCCGTAECKD	HLA-DRB1*11:01	865	879	6.27	>0.1
1746	PSPYVKCCGTAECKD	HLA-DRB1*08:01	865	879	6.87	Not predicted
1404	SPYVKCCGTAECKDK	HLA-DRB1*13:01	866	880	5.45	>0.1
1532	SPYVKCCGTAECKDK	HLA-DRB1*11:01	866	880	6.16	>0.1
1747	SPYVKCCGTAECKDK	HLA-DRB1*08:01	866	880	6.87	Not predicted
1405	PYVKCCGTAECKDKS	HLA-DRB1*13:01	867	881	5.45	>0.1
1748	PYVKCCGTAECKDKS	HLA-DRB1*08:01	867	881	6.87	Not predicted
1406	YVKCCGTAECKDKSL	HLA-DRB1*13:01	868	882	5.45	>0.1

1749	YVKCCGTAECKDKSL	HLA-DRB1*08:01	868	882	6.87	Not predicted
1407	VKCCGTAECKDKSLP	HLA-DRB1*13:01	869	883	5.45	>0.1
1750	VKCCGTAECKDKSLP	HLA-DRB1*08:01	869	883	6.87	Not predicted
2145	CKVFTGVYPFMWGGA	HLA-DRB1*15:01	887	901	8.35	>0.1
1952	KVFTGVYPFMWGGAY	HLA-DRB1*15:01	888	902	7.71	>0.1
2125	KVFTGVYPFMWGGAY	HLA-DRB1*08:01	888	902	8.32	Not predicted
2126	VFTGVYPFMWGGAYC	HLA-DRB1*08:01	889	903	8.32	Not predicted
2127	FTGVYPFMWGGAYCF	HLA-DRB1*08:01	890	904	8.32	Not predicted
2408	FTGVYPFMWGGAYCF	HLA-DRB1*07:01	890	904	9.39	>0.1
2128	TGVYPFMWGGAYCFC	HLA-DRB1*08:01	891	905	8.32	Not predicted

1966	GVYPFMWGGAYCFCD	HLA-DRB1*08:01	892	906	7.81	Not predicted
1967	VYPFMWGGAYCFCDT	HLA-DRB1*08:01	893	907	7.81	Not predicted
1968	YPFMWGGAYCFCDTE	HLA-DRB1*08:01	894	908	7.81	Not predicted
1969	PFMWGGAYCFCDTEN	HLA-DRB1*08:01	895	909	7.81	Not predicted
1970	FMWGGAYCFCDTENT	HLA-DRB1*08:01	896	910	7.81	Not predicted
1971	MWGGAYCFCDTENTQ	HLA-DRB1*08:01	897	911	7.81	Not predicted
974	WGGAYCFCDTENTQL	HLA-DRB1*07:01	898	912	3.9	>0.1
1972	WGGAYCFCDTENTQL	HLA-DRB1*08:01	898	912	7.81	Not predicted
2087	WGGAYCFCDTENTQL	HLA-DRB1*04:01	898	912	8.06	>0.1
1265	GGAYCFCDTENTQLS	HLA-DRB1*07:01	899	913	4.96	>0.1

1811	GGAYCFCDTENTQLS	HLA-DRB1*04:01	899	913	6.98	>0.1
1839	GAYCFCDTENTQLSE	HLA-DRB1*07:01	900	914	7.31	>0.1
2021	GAYCFCDTENTQLSE	HLA-DRB1*04:01	900	914	7.92	>0.1
2427	AYCFCDTENTQLSEA	HLA-DRB1*04:01	901	915	9.52	>0.1
1855	ESCKTEFASAYRAHT	HLA-DRB1*08:01	921	935	7.32	Not predicted
2217	ESCKTEFASAYRAHT	HLA-DRB1*11:01	921	935	8.73	>0.1
1351	SCKTEFASAYRAHTA	HLA-DRB1*11:01	922	936	5.21	>0.1
1856	SCKTEFASAYRAHTA	HLA-DRB1*08:01	922	936	7.32	Not predicted
679	CKTEFASAYRAHTAS	HLA-DRB1*11:01	923	937	2.81	>0.1
1857	CKTEFASAYRAHTAS	HLA-DRB1*08:01	923	937	7.32	Not predicted

349	KTEFASAYRAHTASA	HLA-DRB1*11:01	924	938	1.46	0.0734
1858	KTEFASAYRAHTASA	HLA-DRB1*08:01	924	938	7.32	Not predicted
162	TEFASAYRAHTASAS	HLA-DRB1*04:01	925	939	0.84	>0.1
468	TEFASAYRAHTASAS	HLA-DRB1*13:01	925	939	2.16	>0.1
516	TEFASAYRAHTASAS	HLA-DRB1*11:01	925	939	2.31	>0.1
1517	TEFASAYRAHTASAS	HLA-DRB1*01:01	925	939	6	>0.1
1859	TEFASAYRAHTASAS	HLA-DRB1*08:01	925	939	7.32	Not predicted
2212	TEFASAYRAHTASAS	HLA-DRB1*07:01	925	939	8.67	>0.1
2305	TEFASAYRAHTASAS	HLA-DRB1*03:01	925	939	9.05	>0.1
168	**EFASAYRAHTASASA**	**HLA-DRB1*04:01**	**926**	**940**	**0.88**	**>0.1**

469	**EFASAYRAHTASASA**	**HLA-DRB1*13:01**	**926**	**940**	**2.16**	**>0.1**
839	**EFASAYRAHTASASA**	**HLA-DRB1*01:01**	**926**	**940**	**3.36**	**>0.1**
844	**EFASAYRAHTASASA**	**HLA-DRB1*11:01**	**926**	**940**	**3.37**	**>0.1**
1860	EFASAYRAHTASASA	HLA-DRB1*08:01	926	940	7.32	Not predicted
2089	**EFASAYRAHTASASA**	**HLA-DRB1*07:01**	**926**	**940**	**8.08**	**>0.1**
163	**FASAYRAHTASASAK**	**HLA-DRB1*04:01**	**927**	**941**	**0.84**	**>0.1**
385	**FASAYRAHTASASAK**	**HLA-DRB1*01:01**	**927**	**941**	**1.62**	**>0.1**
470	**FASAYRAHTASASAK**	**HLA-DRB1*13:01**	**927**	**941**	**2.16**	**>0.1**
1062	**FASAYRAHTASASAK**	**HLA-DRB1*11:01**	**927**	**941**	**4.33**	**>0.1**
1861	FASAYRAHTASASAK	HLA-DRB1*08:01	927	941	7.32	Not predicted

2093	**FASAYRAHTASASAK**	**HLA-DRB1*07:01**	**927**	**941**	**8.14**	**>0.1**
92	**ASAYRAHTASASAKL**	**HLA-DRB1*01:01**	**928**	**942**	**0.54**	**>0.1**
117	**ASAYRAHTASASAKL**	**HLA-DRB1*04:01**	**928**	**942**	**0.64**	**>0.1**
213	**ASAYRAHTASASAKL**	**HLA-DRB1*07:01**	**928**	**942**	**0.95**	**>0.1**
471	**ASAYRAHTASASAKL**	**HLA-DRB1*13:01**	**928**	**942**	**2.16**	**>0.1**
1063	**ASAYRAHTASASAKL**	**HLA-DRB1*11:01**	**928**	**942**	**4.33**	**>0.1**
2129	ASAYRAHTASASAKL	HLA-DRB1*08:01	928	942	8.32	Not predicted
166	**SAYRAHTASASAKLR**	**HLA-DRB1*04:01**	**929**	**943**	**0.87**	**>0.1**
197	**SAYRAHTASASAKLR**	**HLA-DRB1*07:01**	**929**	**943**	**0.94**	**>0.1**
296	**SAYRAHTASASAKLR**	**HLA-DRB1*01:01**	**929**	**943**	**1.24**	**>0.1**

472	**SAYRAHTASASAKLR**	**HLA-DRB1*13:01**	**929**	**943**	**2.16**	**>0.1**
1064	**SAYRAHTASASAKLR**	**HLA-DRB1*11:01**	**929**	**943**	**4.33**	**>0.1**
2130	SAYRAHTASASAKLR	HLA-DRB1*08:01	929	943	8.32	Not predicted
2280	**SAYRAHTASASAKLR**	**HLA-DRB1*03:01**	**929**	**943**	**9**	**>0.1**
170	**AYRAHTASASAKLRV**	**HLA-DRB1*07:01**	**930**	**944**	**0.88**	**>0.1**
473	**AYRAHTASASAKLRV**	**HLA-DRB1*13:01**	**930**	**944**	**2.16**	**>0.1**
591	**AYRAHTASASAKLRV**	**HLA-DRB1*01:01**	**930**	**944**	**2.55**	**>0.1**
625	**AYRAHTASASAKLRV**	**HLA-DRB1*04:01**	**930**	**944**	**2.61**	**>0.1**
1311	**AYRAHTASASAKLRV**	**HLA-DRB1*11:01**	**930**	**944**	**5.11**	**>0.1**
1533	**AYRAHTASASAKLRV**	**HLA-DRB1*03:01**	**930**	**944**	**6.17**	**>0.1**

2131	AYRAHTASASAKLRV	HLA-DRB1*08:01	930	944	8.32	Not predicted
143	YRAHTASASAKLRVL	HLA-DRB1*07:01	931	945	0.8	>0.1
474	YRAHTASASAKLRVL	HLA-DRB1*13:01	931	945	2.16	>0.1
815	YRAHTASASAKLRVL	HLA-DRB1*04:01	931	945	3.31	>0.1
1094	YRAHTASASAKLRVL	HLA-DRB1*11:01	931	945	4.45	>0.1
1463	YRAHTASASAKLRVL	HLA-DRB1*03:01	931	945	5.79	>0.1
2132	YRAHTASASAKLRVL	HLA-DRB1*08:01	931	945	8.32	Not predicted
145	RAHTASASAKLRVLY	HLA-DRB1*07:01	932	946	0.81	>0.1
658	AHTASASAKLRVLYQ	HLA-DRB1*07:01	933	947	2.71	>0.1
672	HTASASAKLRVLYQG	HLA-DRB1*07:01	934	948	2.78	>0.1

609	ASASAKLRVLYQGNN	HLA-DRB1*08:01	936	950	2.58	Not predicted
1387	ASASAKLRVLYQGNN	HLA-DRB1*11:01	936	950	5.43	>0.1
2095	ASASAKLRVLYQGNN	HLA-DRB1*03:01	936	950	8.18	>0.1
610	SASAKLRVLYQGNNI	HLA-DRB1*08:01	937	951	2.58	Not predicted
1388	SASAKLRVLYQGNNI	HLA-DRB1*11:01	937	951	5.43	>0.1
611	ASAKLRVLYQGNNIT	HLA-DRB1*08:01	938	952	2.58	Not predicted
1310	ASAKLRVLYQGNNIT	HLA-DRB1*15:01	938	952	5.1	>0.1
1421	ASAKLRVLYQGNNIT	HLA-DRB1*11:01	938	952	5.55	>0.1
1442	ASAKLRVLYQGNNIT	HLA-DRB1*04:01	938	952	5.58	>0.1
2048	ASAKLRVLYQGNNIT	HLA-DRB1*13:01	938	952	7.95	>0.1

574	SAKLRVLYQGNNITV	HLA-DRB1*04:01	939	953	2.47	>0.1
612	SAKLRVLYQGNNITV	HLA-DRB1*08:01	939	953	2.58	Not predicted
698	SAKLRVLYQGNNITV	HLA-DRB1*15:01	939	953	2.87	>0.1
1389	SAKLRVLYQGNNITV	HLA-DRB1*11:01	939	953	5.43	>0.1
2049	SAKLRVLYQGNNITV	HLA-DRB1*13:01	939	953	7.95	>0.1
483	AKLRVLYQGNNITVA	HLA-DRB1*04:01	940	954	2.18	>0.1
528	AKLRVLYQGNNITVA	HLA-DRB1*15:01	940	954	2.37	>0.1
613	AKLRVLYQGNNITVA	HLA-DRB1*08:01	940	954	2.58	Not predicted
1372	AKLRVLYQGNNITVA	HLA-DRB1*11:01	940	954	5.39	>0.1
2050	AKLRVLYQGNNITVA	HLA-DRB1*13:01	940	954	7.95	>0.1

466	KLRVLYQGNNITVAA	HLA-DRB1*04:01	941	955	2.14	>0.1
529	KLRVLYQGNNITVAA	HLA-DRB1*15:01	941	955	2.37	0.00793
614	KLRVLYQGNNITVAA	HLA-DRB1*08:01	941	955	2.58	Not predicted
2051	KLRVLYQGNNITVAA	HLA-DRB1*13:01	941	955	7.95	>0.1
2253	KLRVLYQGNNITVAA	HLA-DRB1*11:01	941	955	8.86	>0.1
484	LRVLYQGNNITVAAY	HLA-DRB1*04:01	942	956	2.18	>0.1
615	LRVLYQGNNITVAAY	HLA-DRB1*08:01	942	956	2.58	Not predicted
702	LRVLYQGNNITVAAY	HLA-DRB1*15:01	942	956	2.89	>0.1
2052	LRVLYQGNNITVAAY	HLA-DRB1*13:01	942	956	7.95	>0.1
2270	LRVLYQGNNITVAAY	HLA-DRB1*11:01	942	956	8.96	>0.1

754	RVLYQGNNITVAAYA	HLA-DRB1*04:01	943	957	3.11	>0.1
931	RVLYQGNNITVAAYA	HLA-DRB1*15:01	943	957	3.86	>0.1
2053	RVLYQGNNITVAAYA	HLA-DRB1*13:01	943	957	7.95	>0.1
1465	VLYQGNNITVAAYAN	HLA-DRB1*04:01	944	958	5.81	>0.1
2054	VLYQGNNITVAAYAN	HLA-DRB1*13:01	944	958	7.95	>0.1
2447	LYQGNNITVAAYANG	HLA-DRB1*04:01	945	959	9.59	>0.1
1241	QGNNITVAAYANGDH	HLA-DRB1*08:01	947	961	4.81	Not predicted
1242	GNNITVAAYANGDHA	HLA-DRB1*08:01	948	962	4.81	Not predicted
1243	NNITVAAYANGDHAV	HLA-DRB1*08:01	949	963	4.81	Not predicted
1244	NITVAAYANGDHAVT	HLA-DRB1*08:01	950	964	4.81	Not predicted

1245	ITVAAYANGDHAVTV	HLA-DRB1*08:01	951	965	4.81	Not predicted
1246	TVAAYANGDHAVTVK	HLA-DRB1*08:01	952	966	4.81	Not predicted
1247	VAAYANGDHAVTVKD	HLA-DRB1*08:01	953	967	4.81	Not predicted
1408	ANGDHAVTVKDAKFI	HLA-DRB1*13:01	957	971	5.45	>0.1
1862	ANGDHAVTVKDAKFI	HLA-DRB1*08:01	957	971	7.32	Not predicted
1409	NGDHAVTVKDAKFIV	HLA-DRB1*13:01	958	972	5.45	>0.1
1814	NGDHAVTVKDAKFIV	HLA-DRB1*03:01	958	972	7.05	>0.1
1863	NGDHAVTVKDAKFIV	HLA-DRB1*08:01	958	972	7.32	Not predicted
1410	GDHAVTVKDAKFIVG	HLA-DRB1*13:01	959	973	5.45	>0.1
1530	GDHAVTVKDAKFIVG	HLA-DRB1*03:01	959	973	6.16	>0.1

1864	GDHAVTVKDAKFIVG	HLA-DRB1*08:01	959	973	7.32	Not predicted
1411	DHAVTVKDAKFIVGP	HLA-DRB1*13:01	960	974	5.45	>0.1
1420	DHAVTVKDAKFIVGP	HLA-DRB1*03:01	960	974	5.52	>0.1
1865	DHAVTVKDAKFIVGP	HLA-DRB1*08:01	960	974	7.32	Not predicted
1194	HAVTVKDAKFIVGPM	HLA-DRB1*03:01	961	975	4.74	0.00749
1412	HAVTVKDAKFIVGPM	HLA-DRB1*13:01	961	975	5.45	>0.1
1866	HAVTVKDAKFIVGPM	HLA-DRB1*08:01	961	975	7.32	Not predicted
1413	AVTVKDAKFIVGPMS	HLA-DRB1*13:01	962	976	5.45	>0.1
1867	AVTVKDAKFIVGPMS	HLA-DRB1*08:01	962	976	7.32	Not predicted
2441	AVTVKDAKFIVGPMS	HLA-DRB1*03:01	962	976	9.54	>0.1

1414	VTVKDAKFIVGPMSS	HLA-DRB1*13:01	963	977	5.45	>0.1
1868	VTVKDAKFIVGPMSS	HLA-DRB1*08:01	963	977	7.32	Not predicted
1678	KDAKFIVGPMSSAWT	HLA-DRB1*04:01	966	980	6.65	>0.1
1802	KDAKFIVGPMSSAWT	HLA-DRB1*15:01	966	980	6.95	>0.1
1973	KDAKFIVGPMSSAWT	HLA-DRB1*08:01	966	980	7.81	Not predicted
1679	DAKFIVGPMSSAWTP	HLA-DRB1*04:01	967	981	6.65	>0.1
1803	DAKFIVGPMSSAWTP	HLA-DRB1*15:01	967	981	6.95	>0.1
1974	DAKFIVGPMSSAWTP	HLA-DRB1*08:01	967	981	7.81	Not predicted
1680	AKFIVGPMSSAWTPF	HLA-DRB1*04:01	968	982	6.65	>0.1
1804	AKFIVGPMSSAWTPF	HLA-DRB1*15:01	968	982	6.95	>0.1

1975	AKFIVGPMSSAWTPF	HLA-DRB1*08:01	968	982	7.81	Not predicted
1681	KFIVGPMSSAWTPFD	HLA-DRB1*04:01	969	983	6.65	>0.1
1826	KFIVGPMSSAWTPFD	HLA-DRB1*15:01	969	983	7.16	0.0384
1976	KFIVGPMSSAWTPFD	HLA-DRB1*08:01	969	983	7.81	Not predicted
1836	FIVGPMSSAWTPFDN	HLA-DRB1*04:01	970	984	7.27	>0.1
1977	FIVGPMSSAWTPFDN	HLA-DRB1*08:01	970	984	7.81	Not predicted
2471	FIVGPMSSAWTPFDN	HLA-DRB1*15:01	970	984	9.62	>0.1
1978	IVGPMSSAWTPFDNK	HLA-DRB1*08:01	971	985	7.81	Not predicted
1979	VGPMSSAWTPFDNKI	HLA-DRB1*08:01	972	986	7.81	Not predicted
2229	SSAWTPFDNKIVVYK	HLA-DRB1*08:01	976	990	8.82	Not predicted

2230	SAWTPFDNKIVVYKG	HLA-DRB1*08:01	977	991	8.82	Not predicted
2231	AWTPFDNKIVVYKGD	HLA-DRB1*08:01	978	992	8.82	Not predicted
2232	WTPFDNKIVVYKGDV	HLA-DRB1*08:01	979	993	8.82	Not predicted
580	TPFDNKIVVYKGDVY	HLA-DRB1*15:01	980	994	2.5	>0.1
1869	TPFDNKIVVYKGDVY	HLA-DRB1*08:01	980	994	7.32	Not predicted
586	PFDNKIVVYKGDVYN	HLA-DRB1*15:01	981	995	2.52	>0.1
1343	PFDNKIVVYKGDVYN	HLA-DRB1*08:01	981	995	5.17	Not predicted
544	FDNKIVVYKGDVYNM	HLA-DRB1*15:01	982	996	2.38	>0.1
1344	FDNKIVVYKGDVYNM	HLA-DRB1*08:01	982	996	5.17	Not predicted
1557	FDNKIVVYKGDVYNM	HLA-DRB1*03:01	982	996	6.32	>0.1

1890	FDNKIVVYKGDVYNM	HLA-DRB1*13:01	982	996	7.42	>0.1
568	DNKIVVYKGDVYNMD	HLA-DRB1*15:01	983	997	2.44	0.0327
1256	DNKIVVYKGDVYNMD	HLA-DRB1*03:01	983	997	4.91	>0.1
1345	DNKIVVYKGDVYNMD	HLA-DRB1*08:01	983	997	5.17	Not predicted
1891	DNKIVVYKGDVYNMD	HLA-DRB1*13:01	983	997	7.42	>0.1
2162	DNKIVVYKGDVYNMD	HLA-DRB1*04:01	983	997	8.45	>0.1
576	NKIVVYKGDVYNMDY	HLA-DRB1*15:01	984	998	2.48	>0.1
1250	NKIVVYKGDVYNMDY	HLA-DRB1*03:01	984	998	4.87	>0.1
1346	NKIVVYKGDVYNMDY	HLA-DRB1*08:01	984	998	5.17	Not predicted
1892	NKIVVYKGDVYNMDY	HLA-DRB1*13:01	984	998	7.42	>0.1

2163	NKIVVYKGDVYNMDY	HLA-DRB1*04:01	984	998	8.45	>0.1
929	KIVVYKGDVYNMDYP	HLA-DRB1*15:01	985	999	3.84	>0.1
1347	KIVVYKGDVYNMDYP	HLA-DRB1*08:01	985	999	5.17	Not predicted
1893	KIVVYKGDVYNMDYP	HLA-DRB1*13:01	985	999	7.42	>0.1
2024	KIVVYKGDVYNMDYP	HLA-DRB1*03:01	985	999	7.94	0.00915
2164	KIVVYKGDVYNMDYP	HLA-DRB1*04:01	985	999	8.45	>0.1
1348	IVVYKGDVYNMDYPP	HLA-DRB1*08:01	986	1000	5.17	Not predicted
1543	IVVYKGDVYNMDYPP	HLA-DRB1*15:01	986	1000	6.23	>0.1
1894	IVVYKGDVYNMDYPP	HLA-DRB1*13:01	986	1000	7.42	>0.1
2165	IVVYKGDVYNMDYPP	HLA-DRB1*04:01	986	1000	8.45	>0.1

2262	IVVYKGDVYNMDYPP	HLA-DRB1*03:01	986	1000	8.95	>0.1
1349	VVYKGDVYNMDYPPF	HLA-DRB1*08:01	987	1001	5.17	Not predicted
1895	VVYKGDVYNMDYPPF	HLA-DRB1*13:01	987	1001	7.42	>0.1
2481	VVYKGDVYNMDYPPF	HLA-DRB1*04:01	987	1001	9.7	>0.1
665	VYKGDVYNMDYPPFG	HLA-DRB1*03:01	988	1002	2.75	>0.1
1683	VYKGDVYNMDYPPFG	HLA-DRB1*04:01	988	1002	6.66	>0.1
1870	VYKGDVYNMDYPPFG	HLA-DRB1*08:01	988	1002	7.32	Not predicted
1896	VYKGDVYNMDYPPFG	HLA-DRB1*13:01	988	1002	7.42	>0.1
661	YKGDVYNMDYPPFGA	HLA-DRB1*03:01	989	1003	2.73	>0.1
1696	YKGDVYNMDYPPFGA	HLA-DRB1*04:01	989	1003	6.72	>0.1

1871	YKGDVYNMDYPPFGA	HLA-DRB1*08:01	989	1003	7.32	Not predicted
2348	YKGDVYNMDYPPFGA	HLA-DRB1*13:01	989	1003	9.09	>0.1
722	KGDVYNMDYPPFGAG	HLA-DRB1*03:01	990	1004	2.93	>0.1
1872	KGDVYNMDYPPFGAG	HLA-DRB1*08:01	990	1004	7.32	Not predicted
1881	KGDVYNMDYPPFGAG	HLA-DRB1*04:01	990	1004	7.33	>0.1
2349	KGDVYNMDYPPFGAG	HLA-DRB1*13:01	990	1004	9.09	>0.1
697	GDVYNMDYPPFGAGR	HLA-DRB1*03:01	991	1005	2.87	>0.1
1827	GDVYNMDYPPFGAGR	HLA-DRB1*04:01	991	1005	7.18	>0.1
1873	GDVYNMDYPPFGAGR	HLA-DRB1*08:01	991	1005	7.32	Not predicted
2350	GDVYNMDYPPFGAGR	HLA-DRB1*13:01	991	1005	9.09	>0.1

701	DVYNMDYPPFGAGRP	HLA-DRB1*03:01	992	1006	2.89	>0.1
1874	DVYNMDYPPFGAGRP	HLA-DRB1*08:01	992	1006	7.32	Not predicted
2219	DVYNMDYPPFGAGRP	HLA-DRB1*04:01	992	1006	8.76	>0.1
2351	DVYNMDYPPFGAGRP	HLA-DRB1*13:01	992	1006	9.09	>0.1
1875	VYNMDYPPFGAGRPG	HLA-DRB1*08:01	993	1007	7.32	Not predicted
2352	VYNMDYPPFGAGRPG	HLA-DRB1*13:01	993	1007	9.09	>0.1
1876	YNMDYPPFGAGRPGQ	HLA-DRB1*08:01	994	1008	7.32	Not predicted
2464	NMDYPPFGAGRPGQF	HLA-DRB1*03:01	995	1009	9.61	>0.1
2465	MDYPPFGAGRPGQFG	HLA-DRB1*03:01	996	1010	9.61	>0.1
2466	DYPPFGAGRPGQFGD	HLA-DRB1*03:01	997	1011	9.61	>0.1

2467	YPPFGAGRPGQFGDI	HLA-DRB1*03:01	998	1012	9.61	>0.1
**2468**	**PPFGAGRPGQFGDIQ**	**HLA-DRB1*03:01**	**999**	**1013**	**9.61**	**>0.1**
740	PGQFGDIQSRTPESE	HLA-DRB1*08:01	1006	1020	3.05	Not predicted
741	GQFGDIQSRTPESED	HLA-DRB1*08:01	1007	1021	3.05	Not predicted
742	QFGDIQSRTPESEDV	HLA-DRB1*08:01	1008	1022	3.05	Not predicted
743	FGDIQSRTPESEDVY	HLA-DRB1*08:01	1009	1023	3.05	Not predicted
744	GDIQSRTPESEDVYA	HLA-DRB1*08:01	1010	1024	3.05	Not predicted
745	DIQSRTPESEDVYAN	HLA-DRB1*08:01	1011	1025	3.05	Not predicted
746	IQSRTPESEDVYANT	HLA-DRB1*08:01	1012	1026	3.05	Not predicted
2079	PESEDVYANTQLVLQ	HLA-DRB1*11:01	1017	1031	8.05	>0.1

2080	ESEDVYANTQLVLQR	HLA-DRB1*11:01	1018	1032	8.05	>0.1
2413	ESEDVYANTQLVLQR	HLA-DRB1*03:01	1018	1032	9.46	>0.1
2081	SEDVYANTQLVLQRP	HLA-DRB1*11:01	1019	1033	8.05	>0.1
2442	SEDVYANTQLVLQRP	HLA-DRB1*03:01	1019	1033	9.54	0.0865
2082	EDVYANTQLVLQRPS	HLA-DRB1*11:01	1020	1034	8.05	>0.1
2083	DVYANTQLVLQRPSA	HLA-DRB1*11:01	1021	1035	8.05	>0.1
154	VYANTQLVLQRPSAG	HLA-DRB1*13:01	1022	1036	0.82	>0.1
747	VYANTQLVLQRPSAG	HLA-DRB1*03:01	1022	1036	3.06	>0.1
869	VYANTQLVLQRPSAG	HLA-DRB1*04:01	1022	1036	3.51	>0.1
2084	VYANTQLVLQRPSAG	HLA-DRB1*11:01	1022	1036	8.05	>0.1

2133	VYANTQLVLQRPSAG	HLA-DRB1*08:01	1022	1036	8.32	Not predicted
155	YANTQLVLQRPSAGT	HLA-DRB1*13:01	1023	1037	0.82	>0.1
799	YANTQLVLQRPSAGT	HLA-DRB1*04:01	1023	1037	3.25	>0.1
888	YANTQLVLQRPSAGT	HLA-DRB1*08:01	1023	1037	3.58	Not predicted
1073	YANTQLVLQRPSAGT	HLA-DRB1*03:01	1023	1037	4.34	>0.1
2214	YANTQLVLQRPSAGT	HLA-DRB1*11:01	1023	1037	8.7	>0.1
156	ANTQLVLQRPSAGTV	HLA-DRB1*13:01	1024	1038	0.82	>0.1
732	ANTQLVLQRPSAGTV	HLA-DRB1*04:01	1024	1038	2.96	>0.1
889	ANTQLVLQRPSAGTV	HLA-DRB1*08:01	1024	1038	3.58	Not predicted
1001	ANTQLVLQRPSAGTV	HLA-DRB1*03:01	1024	1038	4.12	>0.1

1954	ANTQLVLQRPSAGTV	HLA-DRB1*11:01	1024	1038	7.73	>0.1
2009	ANTQLVLQRPSAGTV	HLA-DRB1*01:01	1024	1038	7.88	>0.1
157	NTQLVLQRPSAGTVH	HLA-DRB1*13:01	1025	1039	0.82	>0.1
735	NTQLVLQRPSAGTVH	HLA-DRB1*04:01	1025	1039	3	>0.1
890	NTQLVLQRPSAGTVH	HLA-DRB1*08:01	1025	1039	3.58	Not predicted
1002	NTQLVLQRPSAGTVH	HLA-DRB1*03:01	1025	1039	4.12	>0.1
2090	NTQLVLQRPSAGTVH	HLA-DRB1*01:01	1025	1039	8.09	>0.1
2194	NTQLVLQRPSAGTVH	HLA-DRB1*11:01	1025	1039	8.53	>0.1
158	TQLVLQRPSAGTVHV	HLA-DRB1*13:01	1026	1040	0.82	>0.1
758	TQLVLQRPSAGTVHV	HLA-DRB1*04:01	1026	1040	3.13	>0.1

891	TQLVLQRPSAGTVHV	HLA-DRB1*08:01	1026	1040	3.58	Not predicted
1007	TQLVLQRPSAGTVHV	HLA-DRB1*03:01	1026	1040	4.15	>0.1
2254	TQLVLQRPSAGTVHV	HLA-DRB1*01:01	1026	1040	8.87	>0.1
2320	TQLVLQRPSAGTVHV	HLA-DRB1*11:01	1026	1040	9.09	>0.1
159	QLVLQRPSAGTVHVP	HLA-DRB1*13:01	1027	1041	0.82	>0.1
892	QLVLQRPSAGTVHVP	HLA-DRB1*08:01	1027	1041	3.58	Not predicted
1916	QLVLQRPSAGTVHVP	HLA-DRB1*04:01	1027	1041	7.54	>0.1
2321	QLVLQRPSAGTVHVP	HLA-DRB1*11:01	1027	1041	9.09	>0.1
160	LVLQRPSAGTVHVPY	HLA-DRB1*13:01	1028	1042	0.82	>0.1
893	LVLQRPSAGTVHVPY	HLA-DRB1*08:01	1028	1042	3.58	Not predicted

2251	LVLQRPSAGTVHVPY	HLA-DRB1*04:01	1028	1042	8.86	>0.1
894	VLQRPSAGTVHVPYS	HLA-DRB1*08:01	1029	1043	3.58	Not predicted
963	SQAPSGFKYWLKERG	HLA-DRB1*13:01	1043	1057	3.87	>0.1
1175	SQAPSGFKYWLKERG	HLA-DRB1*11:01	1043	1057	4.63	>0.1
1515	SQAPSGFKYWLKERG	HLA-DRB1*08:01	1043	1057	5.97	Not predicted
588	QAPSGFKYWLKERGA	HLA-DRB1*11:01	1044	1058	2.53	>0.1
964	QAPSGFKYWLKERGA	HLA-DRB1*13:01	1044	1058	3.87	>0.1
1516	QAPSGFKYWLKERGA	HLA-DRB1*08:01	1044	1058	5.97	Not predicted
32	APSGFKYWLKERGAS	HLA-DRB1*08:01	1045	1059	0.26	Not predicted
335	APSGFKYWLKERGAS	HLA-DRB1*11:01	1045	1059	1.4	>0.1

965	APSGFKYWLKERGAS	HLA-DRB1*13:01	1045	1059	3.87	>0.1
33	PSGFKYWLKERGASL	HLA-DRB1*08:01	1046	1060	0.26	Not predicted
138	PSGFKYWLKERGASL	HLA-DRB1*11:01	1046	1060	0.74	>0.1
966	PSGFKYWLKERGASL	HLA-DRB1*13:01	1046	1060	3.87	>0.1
1277	PSGFKYWLKERGASL	HLA-DRB1*07:01	1046	1060	5.02	0.0401
2312	PSGFKYWLKERGASL	HLA-DRB1*04:01	1046	1060	9.08	>0.1
2412	PSGFKYWLKERGASL	HLA-DRB1*01:01	1046	1060	9.45	>0.1
34	SGFKYWLKERGASLQ	HLA-DRB1*08:01	1047	1061	0.26	Not predicted
294	SGFKYWLKERGASLQ	HLA-DRB1*11:01	1047	1061	1.23	>0.1
967	SGFKYWLKERGASLQ	HLA-DRB1*13:01	1047	1061	3.87	>0.1

1278	SGFKYWLKERGASLQ	HLA-DRB1*07:01	1047	1061	5.02	>0.1
2091	SGFKYWLKERGASLQ	HLA-DRB1*01:01	1047	1061	8.09	>0.1
2313	SGFKYWLKERGASLQ	HLA-DRB1*04:01	1047	1061	9.08	>0.1
35	GFKYWLKERGASLQH	HLA-DRB1*08:01	1048	1062	0.26	Not predicted
435	GFKYWLKERGASLQH	HLA-DRB1*11:01	1048	1062	1.86	>0.1
968	GFKYWLKERGASLQH	HLA-DRB1*13:01	1048	1062	3.87	>0.1
1279	GFKYWLKERGASLQH	HLA-DRB1*07:01	1048	1062	5.02	>0.1
2104	GFKYWLKERGASLQH	HLA-DRB1*01:01	1048	1062	8.28	0.0599
2314	GFKYWLKERGASLQH	HLA-DRB1*04:01	1048	1062	9.08	>0.1
36	FKYWLKERGASLQHT	HLA-DRB1*08:01	1049	1063	0.26	Not predicted

628	FKYWLKERGASLQHT	HLA-DRB1*11:01	1049	1063	2.63	0.00621
969	FKYWLKERGASLQHT	HLA-DRB1*13:01	1049	1063	3.87	>0.1
1280	FKYWLKERGASLQHT	HLA-DRB1*07:01	1049	1063	5.02	>0.1
2307	FKYWLKERGASLQHT	HLA-DRB1*01:01	1049	1063	9.06	>0.1
2315	FKYWLKERGASLQHT	HLA-DRB1*04:01	1049	1063	9.08	>0.1
37	KYWLKERGASLQHTA	HLA-DRB1*08:01	1050	1064	0.26	Not predicted
805	KYWLKERGASLQHTA	HLA-DRB1*11:01	1050	1064	3.26	>0.1
1281	KYWLKERGASLQHTA	HLA-DRB1*07:01	1050	1064	5.02	>0.1
2188	KYWLKERGASLQHTA	HLA-DRB1*13:01	1050	1064	8.49	>0.1
2308	KYWLKERGASLQHTA	HLA-DRB1*01:01	1050	1064	9.06	>0.1

2316	KYWLKERGASLQHTA	HLA-DRB1*04:01	1050	1064	9.08	>0.1
38	YWLKERGASLQHTAP	HLA-DRB1*08:01	1051	1065	0.26	Not predicted
2103	YWLKERGASLQHTAP	HLA-DRB1*11:01	1051	1065	8.27	>0.1
2189	YWLKERGASLQHTAP	HLA-DRB1*13:01	1051	1065	8.49	>0.1
1437	WLKERGASLQHTAPF	HLA-DRB1*08:01	1052	1066	5.55	Not predicted
1438	LKERGASLQHTAPFG	HLA-DRB1*08:01	1053	1067	5.55	Not predicted
2055	KERGASLQHTAPFGC	HLA-DRB1*13:01	1054	1068	7.95	>0.1
2056	ERGASLQHTAPFGCQ	HLA-DRB1*13:01	1055	1069	7.95	>0.1
2057	RGASLQHTAPFGCQI	HLA-DRB1*13:01	1056	1070	7.95	>0.1
2058	GASLQHTAPFGCQIA	HLA-DRB1*13:01	1057	1071	7.95	>0.1

2059	ASLQHTAPFGCQIAT	HLA-DRB1*13:01	1058	1072	7.95	>0.1
2060	SLQHTAPFGCQIATN	HLA-DRB1*13:01	1059	1073	7.95	>0.1
2061	LQHTAPFGCQIATNP	HLA-DRB1*13:01	1060	1074	7.95	>0.1
2248	LQHTAPFGCQIATNP	HLA-DRB1*04:01	1060	1074	8.84	>0.1
2505	QHTAPFGCQIATNPV	HLA-DRB1*04:01	1061	1075	9.89	>0.1
2506	HTAPFGCQIATNPVR	HLA-DRB1*04:01	1062	1076	9.89	>0.1
2507	TAPFGCQIATNPVRA	HLA-DRB1*04:01	1063	1077	9.89	>0.1
205	APFGCQIATNPVRAM	HLA-DRB1*13:01	1064	1078	0.94	>0.1
2272	APFGCQIATNPVRAM	HLA-DRB1*03:01	1064	1078	8.98	>0.1
2508	APFGCQIATNPVRAM	HLA-DRB1*04:01	1064	1078	9.89	>0.1

206	PFGCQIATNPVRAMN	HLA-DRB1*13:01	1065	1079	0.94	>0.1
2273	PFGCQIATNPVRAMN	HLA-DRB1*03:01	1065	1079	8.98	>0.1
2509	PFGCQIATNPVRAMN	HLA-DRB1*04:01	1065	1079	9.89	>0.1
207	FGCQIATNPVRAMNC	HLA-DRB1*13:01	1066	1080	0.94	>0.1
2274	FGCQIATNPVRAMNC	HLA-DRB1*03:01	1066	1080	8.98	>0.1
2510	FGCQIATNPVRAMNC	HLA-DRB1*04:01	1066	1080	9.89	>0.1
208	GCQIATNPVRAMNCA	HLA-DRB1*13:01	1067	1081	0.94	>0.1
2275	GCQIATNPVRAMNCA	HLA-DRB1*03:01	1067	1081	8.98	>0.1
209	CQIATNPVRAMNCAV	HLA-DRB1*13:01	1068	1082	0.94	>0.1
2276	CQIATNPVRAMNCAV	HLA-DRB1*03:01	1068	1082	8.98	>0.1

210	QIATNPVRAMNCAVG	HLA-DRB1*13:01	1069	1083	0.94	>0.1
306	QIATNPVRAMNCAVG	HLA-DRB1*08:01	1069	1083	1.33	Not predicted
211	IATNPVRAMNCAVGN	HLA-DRB1*13:01	1070	1084	0.94	>0.1
307	IATNPVRAMNCAVGN	HLA-DRB1*08:01	1070	1084	1.33	Not predicted
283	ATNPVRAMNCAVGNM	HLA-DRB1*13:01	1071	1085	1.2	>0.1
308	ATNPVRAMNCAVGNM	HLA-DRB1*08:01	1071	1085	1.33	Not predicted
284	TNPVRAMNCAVGNMP	HLA-DRB1*13:01	1072	1086	1.2	>0.1
309	TNPVRAMNCAVGNMP	HLA-DRB1*08:01	1072	1086	1.33	Not predicted
285	NPVRAMNCAVGNMPI	HLA-DRB1*13:01	1073	1087	1.2	>0.1
310	NPVRAMNCAVGNMPI	HLA-DRB1*08:01	1073	1087	1.33	Not predicted

286	PVRAMNCAVGNMPIS	HLA-DRB1*13:01	1074	1088	1.2	>0.1
311	PVRAMNCAVGNMPIS	HLA-DRB1*08:01	1074	1088	1.33	Not predicted
287	VRAMNCAVGNMPISI	HLA-DRB1*13:01	1075	1089	1.2	>0.1
312	VRAMNCAVGNMPISI	HLA-DRB1*08:01	1075	1089	1.33	Not predicted
935	RAMNCAVGNMPISID	HLA-DRB1*08:01	1076	1090	3.86	Not predicted
936	AMNCAVGNMPISIDI	HLA-DRB1*08:01	1077	1091	3.86	Not predicted
937	MNCAVGNMPISIDIP	HLA-DRB1*08:01	1078	1092	3.86	Not predicted
938	NCAVGNMPISIDIPD	HLA-DRB1*08:01	1079	1093	3.86	Not predicted
939	CAVGNMPISIDIPDA	HLA-DRB1*08:01	1080	1094	3.86	Not predicted
418	AVGNMPISIDIPDAA	HLA-DRB1*03:01	1081	1095	1.77	>0.1

940	AVGNMPISIDIPDAA	HLA-DRB1*08:01	1081	1095	3.86	Not predicted
419	VGNMPISIDIPDAAF	HLA-DRB1*03:01	1082	1096	1.77	>0.1
941	VGNMPISIDIPDAAF	HLA-DRB1*08:01	1082	1096	3.86	Not predicted
348	GNMPISIDIPDAAFT	HLA-DRB1*03:01	1083	1097	1.46	>0.1
190	NMPISIDIPDAAFTR	HLA-DRB1*03:01	1084	1098	0.91	>0.1
387	MPISIDIPDAAFTRV	HLA-DRB1*03:01	1085	1099	1.63	>0.1
420	PISIDIPDAAFTRVV	HLA-DRB1*03:01	1086	1100	1.77	0.0732
421	ISIDIPDAAFTRVVD	HLA-DRB1*03:01	1087	1101	1.77	>0.1
394	DIPDAAFTRVVDAPS	HLA-DRB1*04:01	1090	1104	1.68	>0.1
380	IPDAAFTRVVDAPSL	HLA-DRB1*04:01	1091	1105	1.57	>0.1

373	PDAAFTRVVDAPSLT	HLA-DRB1*04:01	1092	1106	1.54	>0.1
374	DAAFTRVVDAPSLTD	HLA-DRB1*04:01	1093	1107	1.54	>0.1
2401	DAAFTRVVDAPSLTD	HLA-DRB1*08:01	1093	1107	9.35	Not predicted
375	AAFTRVVDAPSLTDM	HLA-DRB1*04:01	1094	1108	1.54	>0.1
2402	AAFTRVVDAPSLTDM	HLA-DRB1*08:01	1094	1108	9.35	Not predicted
1216	AFTRVVDAPSLTDMS	HLA-DRB1*04:01	1095	1109	4.8	>0.1
2403	AFTRVVDAPSLTDMS	HLA-DRB1*08:01	1095	1109	9.35	Not predicted
1263	FTRVVDAPSLTDMSC	HLA-DRB1*04:01	1096	1110	4.96	>0.1
2404	FTRVVDAPSLTDMSC	HLA-DRB1*08:01	1096	1110	9.35	Not predicted
2405	TRVVDAPSLTDMSCE	HLA-DRB1*08:01	1097	1111	9.35	Not predicted

2406	RVVDAPSLTDMSCEV	HLA-DRB1*08:01	1098	1112	9.35	Not predicted
2407	VVDAPSLTDMSCEVS	HLA-DRB1*08:01	1099	1113	9.35	Not predicted
1751	CTHSSDFGGVAIIKY	HLA-DRB1*08:01	1115	1129	6.87	Not predicted
1752	THSSDFGGVAIIKYA	HLA-DRB1*08:01	1116	1130	6.87	Not predicted
2528	THSSDFGGVAIIKYA	HLA-DRB1*11:01	1116	1130	9.99	>0.1
630	HSSDFGGVAIIKYAA	HLA-DRB1*11:01	1117	1131	2.65	>0.1
1753	HSSDFGGVAIIKYAA	HLA-DRB1*08:01	1117	1131	6.87	Not predicted
631	SSDFGGVAIIKYAAS	HLA-DRB1*11:01	1118	1132	2.65	>0.1
1754	SSDFGGVAIIKYAAS	HLA-DRB1*08:01	1118	1132	6.87	Not predicted
2387	SSDFGGVAIIKYAAS	HLA-DRB1*04:01	1118	1132	9.35	>0.1

632	SDFGGVAIIKYAASK	HLA-DRB1*11:01	1119	1133	2.65	>0.1
1755	SDFGGVAIIKYAASK	HLA-DRB1*08:01	1119	1133	6.87	Not predicted
2374	SDFGGVAIIKYAASK	HLA-DRB1*04:01	1119	1133	9.32	>0.1
2476	SDFGGVAIIKYAASK	HLA-DRB1*15:01	1119	1133	9.65	>0.1
633	DFGGVAIIKYAASKK	HLA-DRB1*11:01	1120	1134	2.65	>0.1
649	DFGGVAIIKYAASKK	HLA-DRB1*15:01	1120	1134	2.66	>0.1
1682	DFGGVAIIKYAASKK	HLA-DRB1*04:01	1120	1134	6.65	>0.1
1756	DFGGVAIIKYAASKK	HLA-DRB1*08:01	1120	1134	6.87	Not predicted
2140	DFGGVAIIKYAASKK	HLA-DRB1*03:01	1120	1134	8.34	>0.1
462	FGGVAIIKYAASKKG	HLA-DRB1*15:01	1121	1135	2.09	>0.1

634	FGGVAIIKYAASKKG	HLA-DRB1*11:01	1121	1135	2.65	>0.1
1045	FGGVAIIKYAASKKG	HLA-DRB1*13:01	1121	1135	4.25	>0.1
1547	FGGVAIIKYAASKKG	HLA-DRB1*04:01	1121	1135	6.24	>0.1
1757	FGGVAIIKYAASKKG	HLA-DRB1*08:01	1121	1135	6.87	Not predicted
2141	FGGVAIIKYAASKKG	HLA-DRB1*03:01	1121	1135	8.34	>0.1
2365	FGGVAIIKYAASKKG	HLA-DRB1*07:01	1121	1135	9.21	>0.1
460	GGVAIIKYAASKKGK	HLA-DRB1*15:01	1122	1136	2.07	>0.1
1046	GGVAIIKYAASKKGK	HLA-DRB1*13:01	1122	1136	4.25	>0.1
1390	GGVAIIKYAASKKGK	HLA-DRB1*11:01	1122	1136	5.43	>0.1
1548	GGVAIIKYAASKKGK	HLA-DRB1*04:01	1122	1136	6.24	>0.1

2142	GGVAIIKYAASKKGK	HLA-DRB1*03:01	1122	1136	8.34	>0.1
2366	GGVAIIKYAASKKGK	HLA-DRB1*07:01	1122	1136	9.21	>0.1
2514	GGVAIIKYAASKKGK	HLA-DRB1*08:01	1122	1136	9.89	Not predicted
623	GVAIIKYAASKKGKC	HLA-DRB1*15:01	1123	1137	2.6	>0.1
1047	GVAIIKYAASKKGKC	HLA-DRB1*13:01	1123	1137	4.25	>0.1
1391	GVAIIKYAASKKGKC	HLA-DRB1*11:01	1123	1137	5.43	0.0241
1549	GVAIIKYAASKKGKC	HLA-DRB1*04:01	1123	1137	6.24	>0.1
2143	GVAIIKYAASKKGKC	HLA-DRB1*03:01	1123	1137	8.34	>0.1
2367	GVAIIKYAASKKGKC	HLA-DRB1*07:01	1123	1137	9.21	>0.1
2515	GVAIIKYAASKKGKC	HLA-DRB1*08:01	1123	1137	9.89	Not predicted

659	VAIIKYAASKKGKCA	HLA-DRB1*15:01	1124	1138	2.71	0.00491
1048	VAIIKYAASKKGKCA	HLA-DRB1*13:01	1124	1138	4.25	>0.1
1392	VAIIKYAASKKGKCA	HLA-DRB1*11:01	1124	1138	5.43	>0.1
1550	VAIIKYAASKKGKCA	HLA-DRB1*04:01	1124	1138	6.24	>0.1
2144	VAIIKYAASKKGKCA	HLA-DRB1*03:01	1124	1138	8.34	0.0239
2368	VAIIKYAASKKGKCA	HLA-DRB1*07:01	1124	1138	9.21	0.0252
2516	VAIIKYAASKKGKCA	HLA-DRB1*08:01	1124	1138	9.89	Not predicted
1049	AIIKYAASKKGKCAV	HLA-DRB1*13:01	1125	1139	4.25	>0.1
1551	AIIKYAASKKGKCAV	HLA-DRB1*04:01	1125	1139	6.24	>0.1
1561	AIIKYAASKKGKCAV	HLA-DRB1*15:01	1125	1139	6.34	>0.1

1929	AIIKYAASKKGKCAV	HLA-DRB1*11:01	1125	1139	7.59	>0.1
2517	AIIKYAASKKGKCAV	HLA-DRB1*08:01	1125	1139	9.89	Not predicted
1050	IIKYAASKKGKCAVH	HLA-DRB1*13:01	1126	1140	4.25	>0.1
1645	IIKYAASKKGKCAVH	HLA-DRB1*15:01	1126	1140	6.54	>0.1
1930	IIKYAASKKGKCAVH	HLA-DRB1*11:01	1126	1140	7.59	>0.1
2518	IIKYAASKKGKCAVH	HLA-DRB1*08:01	1126	1140	9.89	Not predicted
1051	IKYAASKKGKCAVHS	HLA-DRB1*13:01	1127	1141	4.25	>0.1
1931	IKYAASKKGKCAVHS	HLA-DRB1*11:01	1127	1141	7.59	>0.1
2519	IKYAASKKGKCAVHS	HLA-DRB1*08:01	1127	1141	9.89	Not predicted
1932	KYAASKKGKCAVHSM	HLA-DRB1*11:01	1128	1142	7.59	>0.1

1933	YAASKKGKCAVHSMT	HLA-DRB1*11:01	1129	1143	7.59	>0.1
1599	SKKGKCAVHSMTNAV	HLA-DRB1*07:01	1132	1146	6.4	>0.1
1210	KKGKCAVHSMTNAVT	HLA-DRB1*04:01	1133	1147	4.78	>0.1
1835	KKGKCAVHSMTNAVT	HLA-DRB1*07:01	1133	1147	7.26	>0.1
2148	KKGKCAVHSMTNAVT	HLA-DRB1*15:01	1133	1147	8.37	>0.1
2384	KKGKCAVHSMTNAVT	HLA-DRB1*08:01	1133	1147	9.34	Not predicted
2472	KKGKCAVHSMTNAVT	HLA-DRB1*01:01	1133	1147	9.63	>0.1
734	KGKCAVHSMTNAVTI	HLA-DRB1*07:01	1134	1148	2.99	>0.1
1211	KGKCAVHSMTNAVTI	HLA-DRB1*04:01	1134	1148	4.78	>0.1
2006	KGKCAVHSMTNAVTI	HLA-DRB1*15:01	1134	1148	7.84	>0.1

2210	KGKCAVHSMTNAVTI	HLA-DRB1*01:01	1134	1148	8.67	>0.1
2385	KGKCAVHSMTNAVTI	HLA-DRB1*08:01	1134	1148	9.34	Not predicted
1032	GKCAVHSMTNAVTIR	HLA-DRB1*07:01	1135	1149	4.17	>0.1
1192	GKCAVHSMTNAVTIR	HLA-DRB1*01:01	1135	1149	4.72	>0.1
1212	GKCAVHSMTNAVTIR	HLA-DRB1*04:01	1135	1149	4.78	>0.1
1939	GKCAVHSMTNAVTIR	HLA-DRB1*15:01	1135	1149	7.61	>0.1
2386	GKCAVHSMTNAVTIR	HLA-DRB1*08:01	1135	1149	9.34	Not predicted
863	KCAVHSMTNAVTIRE	HLA-DRB1*01:01	1136	1150	3.45	0.0381
1213	KCAVHSMTNAVTIRE	HLA-DRB1*04:01	1136	1150	4.78	>0.1
1445	KCAVHSMTNAVTIRE	HLA-DRB1*07:01	1136	1150	5.61	0.00867

1877	KCAVHSMTNAVTIRE	HLA-DRB1*08:01	1136	1150	7.32	Not predicted
1953	KCAVHSMTNAVTIRE	HLA-DRB1*15:01	1136	1150	7.71	0.0464
1214	CAVHSMTNAVTIREA	HLA-DRB1*04:01	1137	1151	4.78	>0.1
1638	CAVHSMTNAVTIREA	HLA-DRB1*01:01	1137	1151	6.53	>0.1
1878	CAVHSMTNAVTIREA	HLA-DRB1*08:01	1137	1151	7.32	Not predicted
2225	CAVHSMTNAVTIREA	HLA-DRB1*15:01	1137	1151	8.81	>0.1
1248	**AVHSMTNAVTIREAE**	**HLA-DRB1*04:01**	**1138**	**1152**	**4.85**	**>0.1**
1879	AVHSMTNAVTIREAE	HLA-DRB1*08:01	1138	1152	7.32	Not predicted
2357	**AVHSMTNAVTIREAE**	**HLA-DRB1*01:01**	**1138**	**1152**	**9.13**	**>0.1**
1366	**VHSMTNAVTIREAEI**	**HLA-DRB1*04:01**	**1139**	**1153**	**5.27**	**>0.1**

1880	VHSMTNAVTIREAEI	HLA-DRB1*08:01	1139	1153	7.32	Not predicted
942	HSMTNAVTIREAEIE	HLA-DRB1*08:01	1140	1154	3.86	Not predicted
943	SMTNAVTIREAEIEV	HLA-DRB1*08:01	1141	1155	3.86	Not predicted
944	MTNAVTIREAEIEVE	HLA-DRB1*08:01	1142	1156	3.86	Not predicted
945	TNAVTIREAEIEVEG	HLA-DRB1*08:01	1143	1157	3.86	Not predicted
946	NAVTIREAEIEVEGN	HLA-DRB1*08:01	1144	1158	3.86	Not predicted
947	AVTIREAEIEVEGNS	HLA-DRB1*08:01	1145	1159	3.86	Not predicted
948	VTIREAEIEVEGNSQ	HLA-DRB1*08:01	1146	1160	3.86	Not predicted
2487	REAEIEVEGNSQLQI	HLA-DRB1*13:01	1149	1163	9.71	>0.1
2488	EAEIEVEGNSQLQIS	HLA-DRB1*13:01	1150	1164	9.71	>0.1

2489	AEIEVEGNSQLQISF	HLA-DRB1*13:01	1151	1165	9.71	>0.1
2490	EIEVEGNSQLQISFS	HLA-DRB1*13:01	1152	1166	9.71	>0.1
2491	IEVEGNSQLQISFST	HLA-DRB1*13:01	1153	1167	9.71	>0.1
2492	EVEGNSQLQISFSTA	HLA-DRB1*13:01	1154	1168	9.71	>0.1
136	VEGNSQLQISFSTAL	HLA-DRB1*07:01	1155	1169	0.73	>0.1
558	VEGNSQLQISFSTAL	HLA-DRB1*03:01	1155	1169	2.42	>0.1
837	VEGNSQLQISFSTAL	HLA-DRB1*04:01	1155	1169	3.35	>0.1
1934	VEGNSQLQISFSTAL	HLA-DRB1*11:01	1155	1169	7.59	>0.1
1950	VEGNSQLQISFSTAL	HLA-DRB1*15:01	1155	1169	7.67	>0.1
2473	VEGNSQLQISFSTAL	HLA-DRB1*01:01	1155	1169	9.63	>0.1

2493	VEGNSQLQISFSTAL	HLA-DRB1*13:01	1155	1169	9.71	>0.1
137	EGNSQLQISFSTALA	HLA-DRB1*07:01	1156	1170	0.74	>0.1
464	EGNSQLQISFSTALA	HLA-DRB1*04:01	1156	1170	2.13	>0.1
559	EGNSQLQISFSTALA	HLA-DRB1*03:01	1156	1170	2.42	>0.1
1552	EGNSQLQISFSTALA	HLA-DRB1*15:01	1156	1170	6.24	>0.1
2249	EGNSQLQISFSTALA	HLA-DRB1*01:01	1156	1170	8.86	>0.1
164	GNSQLQISFSTALAS	HLA-DRB1*07:01	1157	1171	0.85	>0.1
358	GNSQLQISFSTALAS	HLA-DRB1*04:01	1157	1171	1.49	>0.1
560	GNSQLQISFSTALAS	HLA-DRB1*03:01	1157	1171	2.42	>0.1
1065	GNSQLQISFSTALAS	HLA-DRB1*11:01	1157	1171	4.33	>0.1

1518	GNSQLQISFSTALAS	HLA-DRB1*15:01	1157	1171	6	>0.1
2062	GNSQLQISFSTALAS	HLA-DRB1*13:01	1157	1171	7.95	>0.1
2250	GNSQLQISFSTALAS	HLA-DRB1*01:01	1157	1171	8.86	>0.1
231	NSQLQISFSTALASA	HLA-DRB1*07:01	1158	1172	1.06	>0.1
359	NSQLQISFSTALASA	HLA-DRB1*04:01	1158	1172	1.49	>0.1
561	NSQLQISFSTALASA	HLA-DRB1*03:01	1158	1172	2.42	0.0797
1066	NSQLQISFSTALASA	HLA-DRB1*11:01	1158	1172	4.33	>0.1
1674	NSQLQISFSTALASA	HLA-DRB1*15:01	1158	1172	6.64	0.0302
2063	NSQLQISFSTALASA	HLA-DRB1*13:01	1158	1172	7.95	>0.1
2147	NSQLQISFSTALASA	HLA-DRB1*01:01	1158	1172	8.36	0.0304

345	SQLQISFSTALASAE	HLA-DRB1*07:01	1159	1173	1.45	>0.1
360	SQLQISFSTALASAE	HLA-DRB1*04:01	1159	1173	1.49	>0.1
562	SQLQISFSTALASAE	HLA-DRB1*03:01	1159	1173	2.42	>0.1
1067	SQLQISFSTALASAE	HLA-DRB1*11:01	1159	1173	4.33	>0.1
1447	SQLQISFSTALASAE	HLA-DRB1*01:01	1159	1173	5.63	>0.1
1703	SQLQISFSTALASAE	HLA-DRB1*15:01	1159	1173	6.76	>0.1
2064	SQLQISFSTALASAE	HLA-DRB1*13:01	1159	1173	7.95	>0.1
361	QLQISFSTALASAEF	HLA-DRB1*04:01	1160	1174	1.49	>0.1
448	QLQISFSTALASAEF	HLA-DRB1*07:01	1160	1174	1.98	0.00823
563	QLQISFSTALASAEF	HLA-DRB1*03:01	1160	1174	2.42	>0.1

1068	QLQISFSTALASAEF	HLA-DRB1*11:01	1160	1174	4.33	>0.1
1624	QLQISFSTALASAEF	HLA-DRB1*01:01	1160	1174	6.42	>0.1
2065	QLQISFSTALASAEF	HLA-DRB1*13:01	1160	1174	7.95	>0.1
415	LQISFSTALASAEFR	HLA-DRB1*04:01	1161	1175	1.74	>0.1
564	LQISFSTALASAEFR	HLA-DRB1*03:01	1161	1175	2.42	>0.1
723	LQISFSTALASAEFR	HLA-DRB1*07:01	1161	1175	2.93	>0.1
1069	LQISFSTALASAEFR	HLA-DRB1*11:01	1161	1175	4.33	0.0265
1625	LQISFSTALASAEFR	HLA-DRB1*01:01	1161	1175	6.42	>0.1
2066	LQISFSTALASAEFR	HLA-DRB1*13:01	1161	1175	7.95	>0.1
678	QISFSTALASAEFRV	HLA-DRB1*04:01	1162	1176	2.79	>0.1

1070	QISFSTALASAEFRV	HLA-DRB1*11:01	1162	1176	4.33	>0.1
1440	QISFSTALASAEFRV	HLA-DRB1*03:01	1162	1176	5.58	>0.1
1626	QISFSTALASAEFRV	HLA-DRB1*01:01	1162	1176	6.42	>0.1
2067	QISFSTALASAEFRV	HLA-DRB1*13:01	1162	1176	7.95	>0.1
750	ISFSTALASAEFRVQ	HLA-DRB1*04:01	1163	1177	3.07	>0.1
1071	ISFSTALASAEFRVQ	HLA-DRB1*11:01	1163	1177	4.33	>0.1
1443	ISFSTALASAEFRVQ	HLA-DRB1*03:01	1163	1177	5.61	>0.1
1675	ISFSTALASAEFRVQ	HLA-DRB1*01:01	1163	1177	6.65	>0.1
2068	ISFSTALASAEFRVQ	HLA-DRB1*13:01	1163	1177	7.95	>0.1
949	ALASAEFRVQVCSTQ	HLA-DRB1*08:01	1168	1182	3.86	Not predicted

1370	ALASAEFRVQVCSTQ	HLA-DRB1*04:01	1168	1182	5.38	>0.1
2353	ALASAEFRVQVCSTQ	HLA-DRB1*13:01	1168	1182	9.09	>0.1
950	LASAEFRVQVCSTQV	HLA-DRB1*08:01	1169	1183	3.86	Not predicted
1254	LASAEFRVQVCSTQV	HLA-DRB1*04:01	1169	1183	4.89	>0.1
2191	LASAEFRVQVCSTQV	HLA-DRB1*07:01	1169	1183	8.51	>0.1
2354	LASAEFRVQVCSTQV	HLA-DRB1*13:01	1169	1183	9.09	>0.1
951	ASAEFRVQVCSTQVH	HLA-DRB1*08:01	1170	1184	3.86	Not predicted
1120	ASAEFRVQVCSTQVH	HLA-DRB1*04:01	1170	1184	4.5	>0.1
1787	ASAEFRVQVCSTQVH	HLA-DRB1*13:01	1170	1184	6.91	>0.1
2425	ASAEFRVQVCSTQVH	HLA-DRB1*11:01	1170	1184	9.49	>0.1

2482	ASAEFRVQVCSTQVH	HLA-DRB1*07:01	1170	1184	9.71	>0.1
952	SAEFRVQVCSTQVHC	HLA-DRB1*08:01	1171	1185	3.86	Not predicted
1132	SAEFRVQVCSTQVHC	HLA-DRB1*04:01	1171	1185	4.62	>0.1
1788	SAEFRVQVCSTQVHC	HLA-DRB1*13:01	1171	1185	6.91	>0.1
2483	SAEFRVQVCSTQVHC	HLA-DRB1*11:01	1171	1185	9.71	>0.1
953	AEFRVQVCSTQVHCA	HLA-DRB1*08:01	1172	1186	3.86	Not predicted
1199	AEFRVQVCSTQVHCA	HLA-DRB1*04:01	1172	1186	4.76	>0.1
1789	AEFRVQVCSTQVHCA	HLA-DRB1*13:01	1172	1186	6.91	>0.1
2522	AEFRVQVCSTQVHCA	HLA-DRB1*11:01	1172	1186	9.92	>0.1
954	EFRVQVCSTQVHCAA	HLA-DRB1*08:01	1173	1187	3.86	Not predicted

1790	EFRVQVCSTQVHCAA	HLA-DRB1*13:01	1173	1187	6.91	>0.1
955	FRVQVCSTQVHCAAE	HLA-DRB1*08:01	1174	1188	3.86	Not predicted
1791	FRVQVCSTQVHCAAE	HLA-DRB1*13:01	1174	1188	6.91	>0.1
1029	RVQVCSTQVHCAAEC	HLA-DRB1*08:01	1175	1189	4.16	Not predicted
1792	RVQVCSTQVHCAAEC	HLA-DRB1*13:01	1175	1189	6.91	>0.1
1030	VQVCSTQVHCAAECH	HLA-DRB1*08:01	1176	1190	4.16	Not predicted
1793	VQVCSTQVHCAAECH	HLA-DRB1*13:01	1176	1190	6.91	>0.1
382	HPPKDHIVNYPASHT	HLA-DRB1*15:01	1190	1204	1.57	>0.1
616	HPPKDHIVNYPASHT	HLA-DRB1*08:01	1190	1204	2.58	Not predicted
1794	HPPKDHIVNYPASHT	HLA-DRB1*13:01	1190	1204	6.91	>0.1

377	PPKDHIVNYPASHTT	HLA-DRB1*15:01	1191	1205	1.55	>0.1
617	PPKDHIVNYPASHTT	HLA-DRB1*08:01	1191	1205	2.58	Not predicted
1795	PPKDHIVNYPASHTT	HLA-DRB1*13:01	1191	1205	6.91	>0.1
340	PKDHIVNYPASHTTL	HLA-DRB1*15:01	1192	1206	1.4	0.043
618	PKDHIVNYPASHTTL	HLA-DRB1*08:01	1192	1206	2.58	Not predicted
1796	PKDHIVNYPASHTTL	HLA-DRB1*13:01	1192	1206	6.91	>0.1
347	KDHIVNYPASHTTLG	HLA-DRB1*15:01	1193	1207	1.45	>0.1
619	KDHIVNYPASHTTLG	HLA-DRB1*08:01	1193	1207	2.58	Not predicted
1096	KDHIVNYPASHTTLG	HLA-DRB1*04:01	1193	1207	4.47	>0.1
1797	KDHIVNYPASHTTLG	HLA-DRB1*13:01	1193	1207	6.91	>0.1

384	DHIVNYPASHTTLGV	HLA-DRB1*15:01	1194	1208	1.61	>0.1
620	DHIVNYPASHTTLGV	HLA-DRB1*08:01	1194	1208	2.58	Not predicted
1126	DHIVNYPASHTTLGV	HLA-DRB1*04:01	1194	1208	4.55	>0.1
1798	DHIVNYPASHTTLGV	HLA-DRB1*13:01	1194	1208	6.91	>0.1
621	HIVNYPASHTTLGVQ	HLA-DRB1*08:01	1195	1209	2.58	Not predicted
1072	HIVNYPASHTTLGVQ	HLA-DRB1*15:01	1195	1209	4.33	>0.1
1799	HIVNYPASHTTLGVQ	HLA-DRB1*13:01	1195	1209	6.91	>0.1
1947	HIVNYPASHTTLGVQ	HLA-DRB1*04:01	1195	1209	7.66	>0.1
622	IVNYPASHTTLGVQD	HLA-DRB1*08:01	1196	1210	2.58	Not predicted
1800	IVNYPASHTTLGVQD	HLA-DRB1*13:01	1196	1210	6.91	>0.1

1812	IVNYPASHTTLGVQD	HLA-DRB1*15:01	1196	1210	7	>0.1
2166	IVNYPASHTTLGVQD	HLA-DRB1*04:01	1196	1210	8.48	>0.1
2220	SHTTLGVQDISATAM	HLA-DRB1*04:01	1202	1216	8.76	>0.1
1560	HTTLGVQDISATAMS	HLA-DRB1*04:01	1203	1217	6.34	>0.1
1393	TTLGVQDISATAMSW	HLA-DRB1*04:01	1204	1218	5.45	>0.1
1446	TLGVQDISATAMSWV	HLA-DRB1*04:01	1205	1219	5.62	>0.1
2226	LGVQDISATAMSWVQ	HLA-DRB1*04:01	1206	1220	8.82	>0.1
1935	DISATAMSWVQKITG	HLA-DRB1*11:01	1210	1224	7.59	>0.1
1936	ISATAMSWVQKITGG	HLA-DRB1*11:01	1211	1225	7.59	>0.1
39	SATAMSWVQKITGGV	HLA-DRB1*08:01	1212	1226	0.26	Not predicted

856	SATAMSWVQKITGGV	HLA-DRB1*11:01	1212	1226	3.42	>0.1
1897	SATAMSWVQKITGGV	HLA-DRB1*13:01	1212	1226	7.42	>0.1
40	ATAMSWVQKITGGVG	HLA-DRB1*08:01	1213	1227	0.26	Not predicted
857	ATAMSWVQKITGGVG	HLA-DRB1*11:01	1213	1227	3.42	>0.1
1898	ATAMSWVQKITGGVG	HLA-DRB1*13:01	1213	1227	7.42	>0.1
41	TAMSWVQKITGGVGL	HLA-DRB1*08:01	1214	1228	0.26	Not predicted
858	TAMSWVQKITGGVGL	HLA-DRB1*11:01	1214	1228	3.42	>0.1
1899	TAMSWVQKITGGVGL	HLA-DRB1*13:01	1214	1228	7.42	>0.1
42	AMSWVQKITGGVGLV	HLA-DRB1*08:01	1215	1229	0.26	Not predicted
1316	AMSWVQKITGGVGLV	HLA-DRB1*11:01	1215	1229	5.13	>0.1

1900	AMSWVQKITGGVGLV	HLA-DRB1*13:01	1215	1229	7.42	>0.1
43	MSWVQKITGGVGLVV	HLA-DRB1*08:01	1216	1230	0.26	Not predicted
1282	MSWVQKITGGVGLVV	HLA-DRB1*11:01	1216	1230	5.02	>0.1
1901	MSWVQKITGGVGLVV	HLA-DRB1*13:01	1216	1230	7.42	>0.1
44	SWVQKITGGVGLVVA	HLA-DRB1*08:01	1217	1231	0.26	Not predicted
1902	SWVQKITGGVGLVVA	HLA-DRB1*13:01	1217	1231	7.42	>0.1
45	WVQKITGGVGLVVAV	HLA-DRB1*08:01	1218	1232	0.26	Not predicted
1903	WVQKITGGVGLVVAV	HLA-DRB1*13:01	1218	1232	7.42	>0.1
2359	QKITGGVGLVVAVAA	HLA-DRB1*11:01	1220	1234	9.16	>0.1
16	ITGGVGLVVAVAALI	HLA-DRB1*07:01	1222	1236	0.14	>0.1

365	ITGGVGLVVAVAALI	HLA-DRB1*13:01	1222	1236	1.52	>0.1
427	ITGGVGLVVAVAALI	HLA-DRB1*01:01	1222	1236	1.81	>0.1
496	ITGGVGLVVAVAALI	HLA-DRB1*03:01	1222	1236	2.22	>0.1
859	ITGGVGLVVAVAALI	HLA-DRB1*11:01	1222	1236	3.42	>0.1
956	ITGGVGLVVAVAALI	HLA-DRB1*04:01	1222	1236	3.87	>0.1
1111	ITGGVGLVVAVAALI	HLA-DRB1*08:01	1222	1236	4.47	Not predicted
1834	ITGGVGLVVAVAALI	HLA-DRB1*15:01	1222	1236	7.22	>0.1
12	TGGVGLVVAVAALIL	HLA-DRB1*07:01	1223	1237	0.09	>0.1
140	TGGVGLVVAVAALIL	HLA-DRB1*01:01	1223	1237	0.77	>0.1
366	TGGVGLVVAVAALIL	HLA-DRB1*13:01	1223	1237	1.52	>0.1

428	TGGVGLVVAVAALIL	HLA-DRB1*11:01	1223	1237	1.82	>0.1
497	TGGVGLVVAVAALIL	HLA-DRB1*03:01	1223	1237	2.22	>0.1
581	TGGVGLVVAVAALIL	HLA-DRB1*15:01	1223	1237	2.5	>0.1
957	TGGVGLVVAVAALIL	HLA-DRB1*04:01	1223	1237	3.87	>0.1
1112	TGGVGLVVAVAALIL	HLA-DRB1*08:01	1223	1237	4.47	Not predicted
8	GGVGLVVAVAALILI	HLA-DRB1*07:01	1224	1238	0.08	>0.1
141	GGVGLVVAVAALILI	HLA-DRB1*01:01	1224	1238	0.77	>0.1
367	GGVGLVVAVAALILI	HLA-DRB1*13:01	1224	1238	1.52	>0.1
429	GGVGLVVAVAALILI	HLA-DRB1*11:01	1224	1238	1.82	>0.1
498	GGVGLVVAVAALILI	HLA-DRB1*03:01	1224	1238	2.22	>0.1

518	GGVGLVVAVAALILI	HLA-DRB1*15:01	1224	1238	2.32	>0.1
958	GGVGLVVAVAALILI	HLA-DRB1*04:01	1224	1238	3.87	>0.1
1113	GGVGLVVAVAALILI	HLA-DRB1*08:01	1224	1238	4.47	Not predicted
6	**GVGLVVAVAALILIV**	**HLA-DRB1*07:01**	**1225**	**1239**	**0.04**	**>0.1**
108	**GVGLVVAVAALILIV**	**HLA-DRB1*01:01**	**1225**	**1239**	**0.6**	**>0.1**
368	**GVGLVVAVAALILIV**	**HLA-DRB1*13:01**	**1225**	**1239**	**1.52**	**>0.1**
430	**GVGLVVAVAALILIV**	**HLA-DRB1*11:01**	**1225**	**1239**	**1.82**	**>0.1**
475	**GVGLVVAVAALILIV**	**HLA-DRB1*15:01**	**1225**	**1239**	**2.17**	**>0.1**
499	**GVGLVVAVAALILIV**	**HLA-DRB1*03:01**	**1225**	**1239**	**2.22**	**>0.1**
959	**GVGLVVAVAALILIV**	**HLA-DRB1*04:01**	**1225**	**1239**	**3.87**	**>0.1**

1114	GVGLVVAVAALILIV	HLA-DRB1*08:01	1225	1239	4.47	Not predicted
7	VGLVVAVAALILIVV	HLA-DRB1*07:01	1226	1240	0.04	>0.1
109	VGLVVAVAALILIVV	HLA-DRB1*01:01	1226	1240	0.6	>0.1
369	VGLVVAVAALILIVV	HLA-DRB1*13:01	1226	1240	1.52	>0.1
386	VGLVVAVAALILIVV	HLA-DRB1*15:01	1226	1240	1.62	>0.1
431	VGLVVAVAALILIVV	HLA-DRB1*11:01	1226	1240	1.82	>0.1
500	VGLVVAVAALILIVV	HLA-DRB1*03:01	1226	1240	2.22	>0.1
960	VGLVVAVAALILIVV	HLA-DRB1*04:01	1226	1240	3.87	>0.1
1115	VGLVVAVAALILIVV	HLA-DRB1*08:01	1226	1240	4.47	Not predicted
13	GLVVAVAALILIVVL	HLA-DRB1*07:01	1227	1241	0.09	>0.1

331	GLVVAVAALILIVVL	HLA-DRB1*01:01	1227	1241	1.36	>0.1
370	GLVVAVAALILIVVL	HLA-DRB1*13:01	1227	1241	1.52	>0.1
432	GLVVAVAALILIVVL	HLA-DRB1*11:01	1227	1241	1.82	>0.1
439	GLVVAVAALILIVVL	HLA-DRB1*15:01	1227	1241	1.91	>0.1
501	GLVVAVAALILIVVL	HLA-DRB1*03:01	1227	1241	2.22	>0.1
1116	GLVVAVAALILIVVL	HLA-DRB1*08:01	1227	1241	4.47	Not predicted
1813	GLVVAVAALILIVVL	HLA-DRB1*04:01	1227	1241	7.02	>0.1
371	LVVAVAALILIVVLC	HLA-DRB1*13:01	1228	1242	1.52	>0.1
459	LVVAVAALILIVVLC	HLA-DRB1*11:01	1228	1242	2.06	>0.1
502	LVVAVAALILIVVLC	HLA-DRB1*03:01	1228	1242	2.22	>0.1

915	LVVAVAALILIVVLC	HLA-DRB1*01:01	1228	1242	3.72	>0.1
1005	LVVAVAALILIVVLC	HLA-DRB1*07:01	1228	1242	4.14	>0.1
1117	LVVAVAALILIVVLC	HLA-DRB1*08:01	1228	1242	4.47	Not predicted
1257	LVVAVAALILIVVLC	HLA-DRB1*15:01	1228	1242	4.91	>0.1
2260	LVVAVAALILIVVLC	HLA-DRB1*04:01	1228	1242	8.92	>0.1
167	VVAVAALILIVVLCV	HLA-DRB1*11:01	1229	1243	0.87	>0.1
288	VVAVAALILIVVLCV	HLA-DRB1*13:01	1229	1243	1.2	>0.1
425	VVAVAALILIVVLCV	HLA-DRB1*15:01	1229	1243	1.79	>0.1
656	VVAVAALILIVVLCV	HLA-DRB1*03:01	1229	1243	2.71	>0.1
832	VVAVAALILIVVLCV	HLA-DRB1*08:01	1229	1243	3.31	Not predicted

2005	VVAVAALILIVVLCV	HLA-DRB1*04:01	1229	1243	7.84	>0.1
192	VAVAALILIVVLCVS	HLA-DRB1*11:01	1230	1244	0.92	>0.1
289	VAVAALILIVVLCVS	HLA-DRB1*13:01	1230	1244	1.2	>0.1
388	VAVAALILIVVLCVS	HLA-DRB1*08:01	1230	1244	1.63	Not predicted
461	VAVAALILIVVLCVS	HLA-DRB1*15:01	1230	1244	2.07	>0.1
751	VAVAALILIVVLCVS	HLA-DRB1*03:01	1230	1244	3.1	>0.1
2150	VAVAALILIVVLCVS	HLA-DRB1*04:01	1230	1244	8.38	>0.1
246	AVAALILIVVLCVSF	HLA-DRB1*11:01	1231	1245	1.12	>0.1
290	AVAALILIVVLCVSF	HLA-DRB1*13:01	1231	1245	1.2	>0.1
389	AVAALILIVVLCVSF	HLA-DRB1*08:01	1231	1245	1.63	Not predicted

463	AVAALILIVVLCVSF	HLA-DRB1*15:01	1231	1245	2.11	>0.1
589	AVAALILIVVLCVSF	HLA-DRB1*03:01	1231	1245	2.54	>0.1
2097	AVAALILIVVLCVSF	HLA-DRB1*04:01	1231	1245	8.21	>0.1
46	VAALILIVVLCVSFS	HLA-DRB1*13:01	1232	1246	0.27	>0.1
76	VAALILIVVLCVSFS	HLA-DRB1*11:01	1232	1246	0.47	>0.1
313	VAALILIVVLCVSFS	HLA-DRB1*08:01	1232	1246	1.33	Not predicted
458	VAALILIVVLCVSFS	HLA-DRB1*15:01	1232	1246	2.05	>0.1
585	VAALILIVVLCVSFS	HLA-DRB1*03:01	1232	1246	2.52	>0.1
695	VAALILIVVLCVSFS	HLA-DRB1*04:01	1232	1246	2.83	>0.1
47	AALILIVVLCVSFSR	HLA-DRB1*13:01	1233	1247	0.27	>0.1

56	AALILIVVLCVSFSR	HLA-DRB1*11:01	1233	1247	0.4	0.0319
314	AALILIVVLCVSFSR	HLA-DRB1*08:01	1233	1247	1.33	Not predicted
417	AALILIVVLCVSFSR	HLA-DRB1*03:01	1233	1247	1.76	>0.1
422	AALILIVVLCVSFSR	HLA-DRB1*15:01	1233	1247	1.77	0.00879
657	AALILIVVLCVSFSR	HLA-DRB1*04:01	1233	1247	2.71	>0.1
48	ALILIVVLCVSFSRH	HLA-DRB1*13:01	1234	1248	0.27	>0.1
94	ALILIVVLCVSFSRH	HLA-DRB1*11:01	1234	1248	0.54	>0.1
315	ALILIVVLCVSFSRH	HLA-DRB1*08:01	1234	1248	1.33	Not predicted
650	ALILIVVLCVSFSRH	HLA-DRB1*03:01	1234	1248	2.69	>0.1
720	ALILIVVLCVSFSRH	HLA-DRB1*04:01	1234	1248	2.92	>0.1

911	ALILIVVLCVSFSRH	HLA-DRB1*15:01	1234	1248	3.7	>0.1
